# Tailoring the properties of (catalytically)-active inclusion bodies

**DOI:** 10.1186/s12934-019-1081-5

**Published:** 2019-02-07

**Authors:** V. D. Jäger, R. Kloss, A. Grünberger, S. Seide, D. Hahn, T. Karmainski, M. Piqueray, J. Embruch, S. Longerich, U. Mackfeld, K.-E. Jaeger, W. Wiechert, M. Pohl, U. Krauss

**Affiliations:** 1 Institut für Molekulare Enzymtechnologie, Heinrich-Heine-Universität Düsseldorf, Forschungszentrum Jülich, 52425 Jülich, Germany; 2 IBG-1: Biotechnology, Forschungszentrum Jülich GmbH, 52425 Jülich, Germany; 3 Multiscale Bioengineering, Bielefeld University, Universitätsstraße 25, 33615 Bielefeld, Germany; 4 Bioeconomy Science Center (BioSC), c/o, Forschungszentrum Jülich, 52425 Jülich, Germany

**Keywords:** Immobilization, Biocatalysis, Inclusion bodies, Protein aggregates, Protein engineering, Structure–function relations, Enzymes

## Abstract

**Background:**

Immobilization is an appropriate tool to ease the handling and recycling of enzymes in biocatalytic processes and to increase their stability. Most of the established immobilization methods require case-to-case optimization, which is laborious and time-consuming. Often, (chromatographic) enzyme purification is required and stable immobilization usually includes additional cross-linking or adsorption steps. We have previously shown in a few case studies that the molecular biological fusion of an aggregation-inducing tag to a target protein induces the intracellular formation of protein aggregates, so called inclusion bodies (IBs), which to a certain degree retain their (catalytic) function. This enables the combination of protein production and immobilization in one step. Hence, those biologically-produced immobilizates were named catalytically-active inclusion bodies (CatIBs) or, in case of proteins without catalytic activity, functional IBs (FIBs). While this strategy has been proven successful, the efficiency, the potential for optimization and important CatIB/FIB properties like yield, activity and morphology have not been investigated systematically.

**Results:**

We here evaluated a CatIB/FIB toolbox of different enzymes and proteins. Different optimization strategies, like linker deletion, C- versus N-terminal fusion and the fusion of alternative aggregation-inducing tags were evaluated. The obtained CatIBs/FIBs varied with respect to formation efficiency, yield, composition and residual activity, which could be correlated to differences in their morphology; as revealed by (electron) microscopy. Last but not least, we demonstrate that the CatIB/FIB formation efficiency appears to be correlated to the solvent-accessible hydrophobic surface area of the target protein, providing a structure-based rationale for our strategy and opening up the possibility to predict its efficiency for any given target protein.

**Conclusion:**

We here provide evidence for the general applicability, predictability and flexibility of the CatIB/FIB immobilization strategy, highlighting the application potential of CatIB-based enzyme immobilizates for synthetic chemistry, biocatalysis and industry.

**Electronic supplementary material:**

The online version of this article (10.1186/s12934-019-1081-5) contains supplementary material, which is available to authorized users.

## Background

For sustainable application, enzyme preparations have to face several requirements, such as long-term stability under process conditions and the possibility of recycling [1]. In order to stabilize enzymes, e.g. towards organic solvents or harsh reaction conditions, immobilization is often the preferred strategy, for which a variety of methods are available [2–5]. Enzymes can be bound onto a carrier material by non-covalent adsorption with the risk of enzyme leakage, or by covalent binding, which mostly requires chemical modification using crosslinking agents. An example are cross-linked enzyme aggregates (CLEAs) [6], which do not require any carrier material and stabilize precipitated enzyme aggregates using glutaraldehyde as a crosslinking agent. Another method is encapsulation of the biocatalyst in polymeric matrices, e.g. in a highly porous sol–gel [7]. All of these methods, however, need case-to-case optimization, since at present no general-purpose strategy for immobilization is available. Moreover, most of the presented immobilization methods require previous (chromatographic) purification of the biocatalyst, which may raise production costs enormously and thus hampers industrial application [8].

We and others have previously shown that the molecular biological fusion of coiled-coil domains [9–11], small artificial peptides [12–15] and aggregation-prone proteins and domains [16–22] to a target protein, induces the intracellular formation of protein aggregates, so called inclusion bodies (IBs) [23], which, in contrast to the long held view of IBs as inactive intracellular waste deposits [24], can to a certain degree retain their function or, in case of enzymes, their catalytic activity (reviewed recently in [2, 11]. This strategy enables the combination of protein production and immobilization, resulting in (in situ) biologically-produced immobilizates, which we coined catalytically-active IBs (CatIBs) [9–11] or in case of proteins without catalytic activity, functional IBs (FIBs) [25]. Like IBs, CatIBs/FIBs contain predominantly the recombinant target protein [26]. Furthermore, they can be produced fast and cost-efficiently, because any previous purification and subsequent cross-linking steps are dispensable. These properties render the resulting particles beneficial for the application in synthetic chemistry, biocatalysis [9, 16, 27], and biomedicine [28–30].

In contrast to most of the above-mentioned strategies that employed artificial peptides or aggregation-prone proteins, our recently presented strategy relies on the fusion of a naturally-occurring coiled-coil domain for the targeted production of CatIBs/FIBs [9–11]. In these studies the tetrameric coiled-coil domain of the cell-surface protein tetrabrachion (tetramerization domain of tetrabrachion; TDoT) from *Staphylothermus marinus* [31] was fused to a variety of different target enzymes with different complexity: the lipase A from *Bacillus subtilis* (*Bs*LA), a hydroxynitrile lyase from *Arabidopsis thaliana* (*At*HNL), the thiamine-diphosphate (ThDP)-dependent enzyme MenD (2-succinyl-5-enol-pyruvyl-6-hydroxy-3-cyclohexene-1-carboxylate synthase) from *E.* *coli* (*Ec*MenD), and the pyridoxal 5′-phosphate (PLP)-dependent lysine decarboxylase from *E.* *coli* (*Ec*LDC), as well as the yellow fluorescent protein (YFP) [9–11]. Thus, we already demonstrated that the fusion strategy is applicable to a broad spectrum of enzymes as well as fluorescent proteins of the GFP family. In these recent studies, the application of CatIBs in biocatalysis was addressed in more detail, e.g. it could be demonstrated that *At*HNL–CatIBs revealed a higher stability at acidic pH values compared to the soluble enzyme, and could be recycled several times for the production of chiral cyanohydrins in a mono-phasic micro-aqueous reaction system consisting of the buffer-saturated organic solvent, methyl *tert*-butyl ether (MTBE) [9]. CatIBs of the constitutive l-lysine decarboxylase of *E.* *coli* were employed for the efficient biocatalytic production of 1,5-diaminopentane (trivial name: cadaverine) [10]. Moreover, very recently we employed the CatIB strategy for the coimmobilization of two enzymes, namely a benzaldehyde lyase from *Pseudomonas fluorescens* (*Pf*BAL) and an alcohol dehydrogenase from *Ralstonia* sp. (*R*ADH), to facilitate the realization of an integrated enzymatic two-step cascade for the production of (1*R*,2*R*)-1-phenylpropane-1,2-diol, a building block of the calcium channel blocker diltiazem [25]. The resulting *Pf*BAL/*R*ADH Co-CatIBs showed improved stability in the cascade reaction as compared to the soluble enzymes [25]. Improved stability, compared to soluble, purified *Pf*BAL, was also demonstrated for the isolated *Pf*BAL-CatIBs, while additionally it could be shown that, depending on the employed coiled-coil domain, CatIBs can be tailored for the application in different reaction systems [32]. For example, the use of the 3HAMP coiled coil, which was derived from the oxygen sensor protein Aer2 from *Pseudomonas aeruginosa*, as aggregation-inducing tag, resulted in CatIBs that were better suited for the use in biphasic aqueous-organic reaction systems, e.g. with cyclopentyl methyl ether (CPME) as organic phase [32]. In contrast, TDoT-*Pf*BAL CatIBs appeared to be better suited for the use in monophasic buffer/dimethyl sulfoxide (DMSO) mixtures [32]. While demonstrating the application potential of CatIBs, these studies did not fully address differences in aggregation (CatIB/FIB formation) efficiency and characteristics, such as the overall activity compared to the soluble purified enzyme, the composition of the particles, the final yield, or their morphology. Moreover, the flexibility of the approach, i.e. in terms of optimization potential by fusion–protein redesign, and the structural basis for CatIB/FIB formation remain unaddressed.

To fill this gap, in the present contribution we applied our fusion strategy to different fluorescent reporter proteins and various differently complex enzymes and empirically analyzed the properties of already established CatIBs. Using this strategy, for three out of seven target proteins, CatIB/FIB formation was successful, revealing variable CatIB/FIB formation efficiency. Based on this initial success, we set out to evaluate the optimization potential for our strategy by generating redesigned fusion constructs by (i) deleting intradomain linkers, (ii) considering C-terminal instead of N-terminal TDoT fusion and (iii) by employing an alternative coiled-coil domain as aggregation-inducing tag. Employing those simple genetic optimization steps, all of the target proteins that initially failed to produce active aggregates or only did so inefficiently, could successfully be produced as CatIBs/FIBs. Using this wealth of different CatIBs/FIBs, we systematically characterized biotechnologically-relevant properties like residual activities compared to the soluble purified enzyme, yield, particle composition, and morphology. Interestingly, (electron) microscopic studies revealed differences in particle/immobilizate morphology, which could be correlated to different CatIB/FIB properties such as activity retention, yield and composition. Last but not least, we show evidence that aggregation (CatIB/FIB formation) efficiency appears to be correlated to the solvent-accessible hydrophobic surface area of the target enzyme, providing a structure-based rationale for our strategy and opening up the possibility to predict its efficacy for any given target protein.

## Results and discussion

### The toolbox strategy

As outlined in the introduction, our previously presented immobilization strategy relies on the molecular biological fusion of a tetrameric coiled-coil domain to a target enzyme, which induces the formation of catalytically-active inclusion bodies (CatIBs) that in case of non-catalytically-active target proteins, such as fluorescent proteins (FPs), are called functional inclusion bodies (FIBs). In its physiological context, this coiled-coil domain forms a strong superhelix [33, 34] and induces the formation of CatIBs/FIBs by a currently unknown mechanism [11]. The initial gene-fusion-containing expression plasmid was constructed from separate modules so that every part could be easily exchanged or deleted (Additional file 1: Figure S5, A). In all previous constructs the fusion protein contained an N-terminal hexahistidine (His_6_) tag, followed by the TDoT domain fused N-terminally to the target enzyme, via a linker region consisting of a flexible (GGGS)_3_-motif and a protease Factor Xa cleavage site. In contrast to our initial study [9], the starting vector used in this study did not possess the coding sequence for an N-terminal His_6_ tag, as also described recently [10]. To rule out any effect of His_6_-tag removal on the aggregation behaviour, quantified here as the efficiency of CatIB/FIB formation, we compared FIB formation for a TDoT-L-YFP construct with and without N-terminal His_6_-tag (Additional file 1: Figure S1). CatIB/FIB formation efficiency is hereby defined as the activity, or in case of FPs, fluorescence, of the insoluble IB-containing pellet fraction (P) relative to the activity/fluorescence of the crude cell extract (CCE, set to 100%). For both constructs, similar fluorescence was detected in the insoluble IB-containing fraction of the corresponding lysates, suggesting that the His_6_ tag has no influence on the aggregation inducing behaviour of the TDoT domain (Additional file 1: Figure S1). Therefore, to simplify the previous vector design, all further constructs were generated without His_6_ tag.

To further validate the broad applicability of our CatIB/FIB strategy, we here employed simple FPs, for easy detection and microscopic observation of FIB formation, and generated CatIBs of various differently complex target enzymes to enable catalytic characterization. As target FPs we selected a monomeric version of the enhanced yellow fluorescent protein [35, 36] (YFP; 27.1 kDa) (for details regarding the employed YFP version see “Methods”) and mCherry (26.7. kDa), a monomeric red fluorescent protein [37]. As target enzymes, two alcohol dehydrogenases (*R*ADH from *Ralstonia* sp. and *Lb*ADH from *Lactobacillus brevis*) and two ThDP-dependent enzymes [benzoylformate decarboxylase from *Pseudomonas putida* (*Pp*BFD) and benzaldehyde lyase from *Pseudomonas fluorescence* (*Pf*BAL)] were added to the CatIB toolbox. *R*ADH and *Lb*ADH are NADPH-dependent tetrameric enzymes with a subunit size of about 27 kDa [38–40]. *R*ADH requires Ca^2+^-ions for its stability [41], whereas Mg^2+^-ions are important for *Lb*ADH to maintain its structural integrity and catalytic activity [42]. *Pf*BAL [43] and *Pp*BFD [44–47] are thiamine-diphosphate (ThDP) and Mg^2+^-ion dependent tetrameric enzymes with a subunit size of 60 kDa (*Pf*BAL) and 56 kDa (*Pp*BFD). CatIBs of *Pf*BAL as well as *R*ADH have recently been described [25]. For *Pp*BFD, we used the variant L476Q with enhanced carboligation activity [48]. The by far biggest enzyme tested as CatIBs is *Ec*LDC, the constitutive lysine decarboxylase from *Escherichia coli* [49]. This pyridoxal 5′-phosphate (PLP)-dependent enzyme forms a decamer that comprises five dimers with a subunit size of 80.6 kDa. The biocatalytic application of *Ec*LDC-CatIBs was recently demonstrated [10].

### Formation of CatIBs/FIBs by N-terminal TDoT fusion

**Fig. 1:**
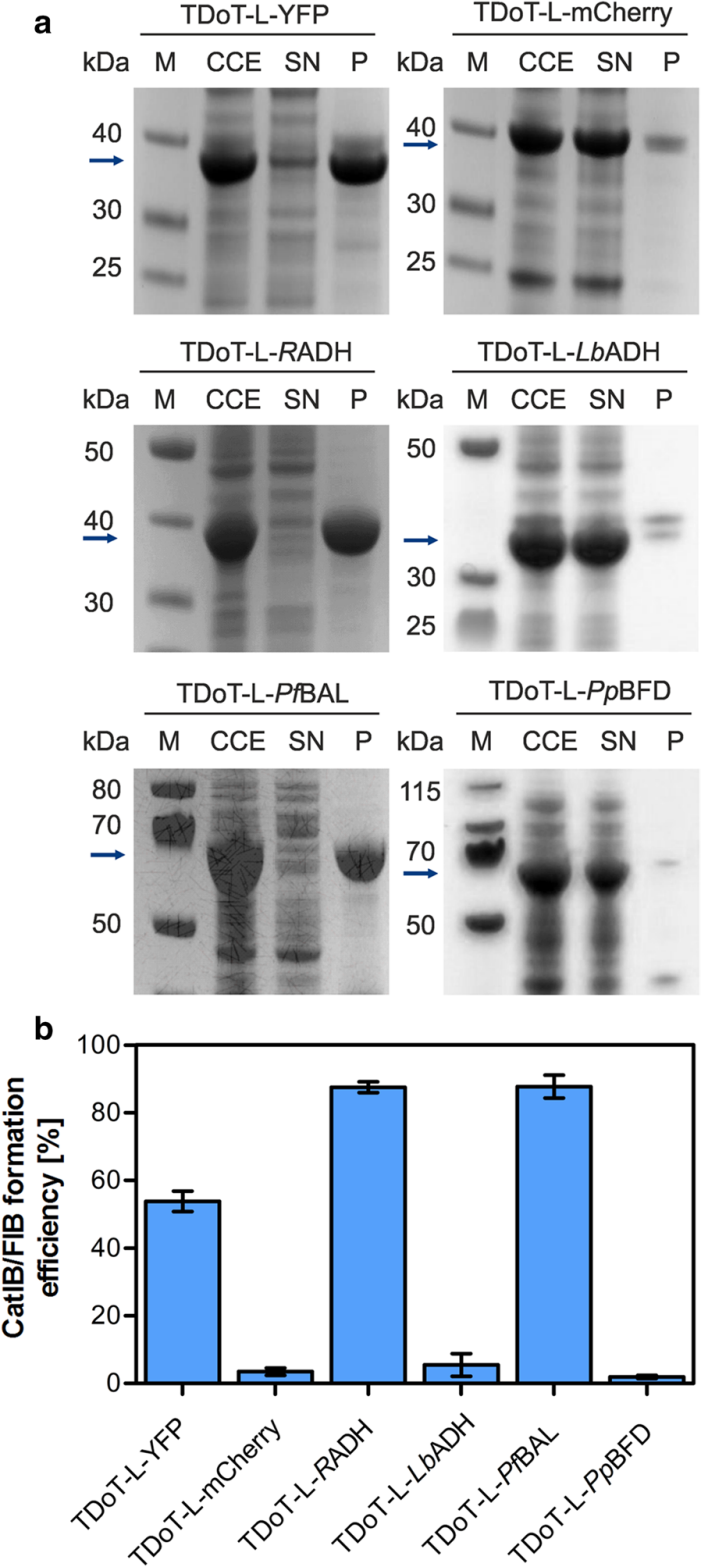
Evaluation of the CatIB/FIB strategy by **a** SDS-PAGE analysis of CatIB/FIB-production and **b** CatIB/FIB formation efficiency for TDoT-L-YFP (Data taken from [25]), TDoT-L-mCherry, TDoT-L-*R*ADH (Data taken from [25]), TDoT-L-*Lb*ADH, TDoT-L-*Pf*BAL (Data taken from [25]), and TDoT-L-*Pp*BFD. After cell disruption, the crude cell extract (CCE) was separated by centrifugation into the soluble protein containing supernatant (SN) and the insoluble IB-containing pellet (P) fractions. **a** SDS-PAGE analysis of the respective protein/enzyme fractions: CCE, SN, and P. The molecular mass of the respective fusion proteins is indicated by arrows (TDoT-L-YFP: 34.6 kDa, TDoT-L-mCherry: 34.3 kDa, TDoT-L-*R*ADH: 34.3 kDa, TDoT-L-*Lb*ADH: 34.3 kDa, TDoT-L-*Pf*BAL: 66.5 kDa, TDoT-L-*Pp*BFD: 65.3 kDa). The protein content in the SN was measured using the Bradford method [87]. **b** CatIB/FIB formation efficiency quantified as the activity/fluorescence in P fractions expressed relative to the activity/fluorescence of the CCE (set to 100%). The complete datasets illustrating the distribution of activity/fluorescence in the CCE, SN and P fractions can be found in Additional file 1: Figure S2. Note: the P fraction was washed once with water and centrifuged again before the activity/fluorescence measurement. The initial rate activities of the ADHs were measured by reduction of 1-phenylethanol (TDoT-L-*Lb*ADH) or cyclohexanone (TDoT-L-*R*ADH) under the consumption of NADPH (Additional file 1: Figure S13a and b). Initial rate activities of the TDoT-L-*Pf*BAL CatIBs and the TDoT-L-*Pp*BFD CatIBs were measured by following the carboligation of 3,5-dimethoxybenzaldehyde (DMBA) to the respective benzoin or by following the decarboxylation of benzoylformate to benzaldehyde (Additional file 1: Figure S13c and d). Error bars correspond to the standard deviation of the mean derived from at least three biological replicates

Our previous design concept for immobilization of additional target proteins was validated by fusing the TDoT-domain N-terminally to the above described target proteins following the fusion strategy depicted in Additional file 1: Figure S5a. CatIBs/FIBs were produced and purified using a standardized protocol [9, 10]. This protocol included standardized expression of the gene fusions in *E.* *coli* BL21(DE3), cell disruption and fractionation of the resulting crude cell extract (CCE) by centrifugation to separate the soluble protein containing fraction (supernatant, SN) from the insoluble, CatIB/FIB-containing fraction (pellet, P). To remove any eventually present soluble protein from the IB pellet, the pellet was resuspended in water, and subsequently centrifuged to separate again the supernatant from the CatIB/FIB containing pellet. All fractions were analyzed by SDS-PAGE (Fig. 1a) and CatIB/FIB formation efficiency was quantified as the activity, or in case of FPs the fluorescence of the once washed IB-containing pellet faction (P) relative to the activity/fluorescence of the CCE (set to 100%) (Fig. 1b). For clarity, only the relative activity in the washed CatIB/FIB-containing pellet fraction is shown in Fig. 1b. The complete datasets illustrating the distribution of activity/fluorescence in the CCE, SN and P fractions can be found in Additional file 1: Figure S2).

Whereas for both TDoT-L-*R*ADH and TDoT-L-*Pf*BAL > 80% of the CCE activity was found in the pellet fraction, only 40% of the YFP fluorescence was detected in the pellet, indicating that, our strategy works less efficient for YFP (Fig. 1b). For TDoT-L-mCherry, TDoT-L-*Lb*ADH, and TDoT-L-*Pp*BFD, this effect was even more pronounced, as for these fusions barely any activity/fluorescence could be detected in the IB-containing pellet fraction (Fig. 1b), whereas the majority of the CCE activity was present in the soluble (SN) fraction (Additional file 1: Figure S2). The same overall trend was also seen in the corresponding SDS-PAGE analyses. The TDoT-L-*Ec*LDC fusion formed large amounts of insoluble aggregates (Additional file 1: Figure S6), which, however, did barely possess any detectable activity (k_cat_ = 6.2*10^−7^ s^−1^). In conclusion, our previously presented fusion strategy, relying on the N-terminal fusion of the TDoT coiled-coil domain, was successful for three out of seven of the tested target proteins/enzymes. To evaluate the potential for optimization, we modified our initial strategy generating redesigned fusion constructs by (i) deleting intradomain linkers, (ii) considering C-terminal instead of N-terminal TDoT fusion and (iii) by employing an alternative coiled-coil domain as aggregation inducing tag.

### Concepts to improve the CatIB/FIB formation efficiency

#### Deletion of the linker region

**Fig. 2:**
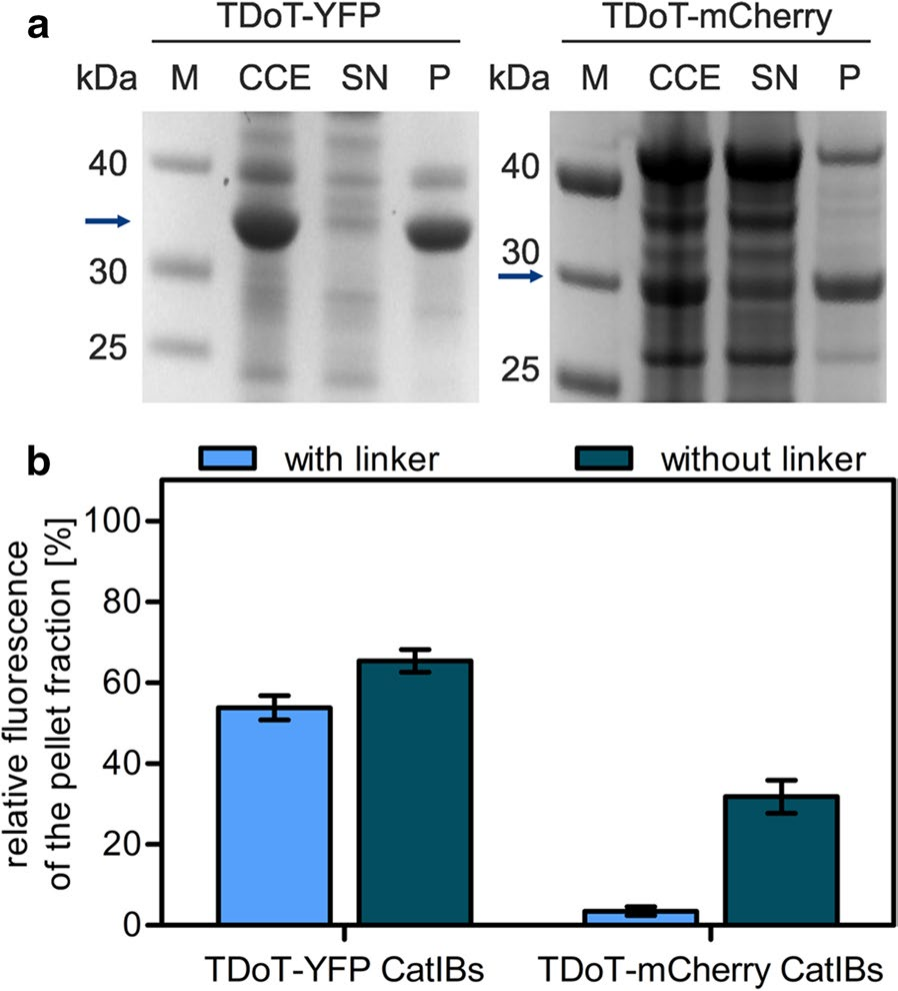
Optimization of the CatIB strategy by excision of the linker region. CatIB formation was evaluated by **a** SDS-PAGE analysis and **b** FIB formation efficiency for TDoT-YFP and TDoT-mCherry (Data taken from [25]) without linker (dark blue bars) compared to TDoT-L-YFP (Data taken from [25]) and TDoT-L-mCherry with linker (light blue bars). After cell disruption, the crude cell extract (CCE) was separated by centrifugation into the soluble protein containing supernatant (SN) and the insoluble FIB-containing pellet (P) fraction. Sample preparation for SDS-PAGE analysis and determination of the FIB formation efficiency was carried out as described in Fig. 1. **a** SDS-PAGE analysis of the respective protein fractions: CCE, SN, and P. The molecular mass of the target fusion proteins is indicated by arrows (TDoT-YFP: 33.1 kDa, TDoT-mCherry: 32.7 kDa). **b** FIB formation efficiency quantified as the fluorescence in P fractions expressed relative to the fluorescence of the CCE (set to 100%). The complete datasets illustrating the distribution of fluorescence in the CCE, SN and P fractions can be found in Additional file 1: Figure S3. Error bars correspond to the standard deviation of the mean derived from at least three biological replicates

From previous studies it is known that the linker employed for fusion protein design can have a large impact on fusion protein functionality [50, 51]. Therefore, as a first optimization approach, the influence of deleting the linker polypeptide that in our fusion proteins connect the TDoT coiled-coil domain with the target enzyme/protein, was exemplarily tested for the TDoT-L-mCherry fusion protein, which almost exclusively remained in the supernatant (SN) after cell disruption (96.8%) (Additional file 1: Figure S2a) and barely any fluorescence was detectable in the insoluble FIB-containing pellet (Fig. 1b). Additionally, the same optimization strategy was tested for TDoT-L-YFP, for which only 40% of the total fluorescence of the CCE was found in the pellet fraction (Fig. 1b and Additional file 1: Figure S2a). Therefore, the fusion variants TDoT-YFP and TDoT-mCherry were generated, which lack the (GGGS)_3_ linker motif as well as the Factor Xa cleavage site (Additional file 1: Figure S5a). Deletion of the linker resulted in about 10% increased fluorescence in the FIB-containing pellet fraction (P) of TDoT-YFP. The improvement was more pronounced for TDoT-mCherry. Here, the fluorescence in the pellet fraction increased by almost 30% (Fig. 2), which is also apparent from the corresponding SDS-PAGE analysis (Fig. 2a; compare to Fig. 1a; TDoT-L-mCherry).

This improvement of the FIB-formation efficiency might hereby be related to a higher rigidity of the fusion protein, due to deletion of the linker. In conclusion, linker deletion appears to be one suitable strategy to improve the CatIB/FIB formation efficiency for difficult target proteins.

#### C-terminal TDoT-domain fusion

When designing N-terminal or C-terminal fusion proteins of multimeric proteins, it is instrumental to consider steric constrains imposed by the quaternary structure, i.e. with regard to the location of the termini. We therefore analyzed the structures of all our multimeric target proteins (*R*ADH, *Lb*ADH, *Pf*BAL, *Pp*BFD and *Ec*LDC) for the accessibility of the N- and C-terminus (Additional file 1: Figure S4). For *R*ADH, *Lb*ADH, *Pf*BAL and *Pp*BFD, the N-termini are localized at the protein surface facing outwards and should thus be accessible for TDoT fusion (Additional file 1: Figure S4a–d) without impacting the formation of the multimer. Thus, C-terminal TDoT fusion was not considered in these cases. In contrast, in *Ec*LDC, the N-termini are buried within the decameric structure of the *Ec*LDC multimer, whereas the C-terminus is located at the protein surface [52] (Additional file 1: Figure S4e). Therefore, N-terminal fusion of the TDoT tag appears not to be feasible for *Ec*LDC, which is corroborated by the observation that the resulting TDoT-L-*Ec*LDC CatIBs, although formed in large amounts, showed barely any activity (k_cat_ = 6.2*10^−7^ s^−1^; vide infra, Table 1). At least dimerization of *Ec*LDC is necessary to form the active site [52, 53]. Thus, it is likely that the N-terminal fusion of the TDoT domain impairs the formation of a correctly folded active site. To improve activity and potentially the CatIB-formation efficiency, we modified our initial TDoT-L-*Ec*LDC construct (Additional file 1: Figure S5a) by shifting the TDoT domain from the N-terminus to the C-terminus of *Ec*LDC (Additional file 1: Figure S5b), resulting in *Ec*LDC-L-TDoT. SDS-PAGE analysis of the resulting *Ec*LDC-L-TDoT CatIBs revealed, similar to the N-terminal fusion, large amounts of protein in the insoluble IB-containing pellet fraction (Additional file 1: Figure S6). However, in contrast to the N-terminal fusion, the activity of the final *Ec*LDC-L-TDoT-CatIB lyophilizate was increased by six orders of magnitude (k_cat_ = 0.71 s^−1^). In conclusion, for target proteins for which structural information is available, the position and accessibility of the N- and C-termini should be considered when generating TDoT fusion proteins to induce CatIB formation, whereas for target proteins with unknown structure both, N-and C-terminal fusions may be considered.

**Table 1:** Characteristics of CatIBs/FIBs All constructs were characterized regarding CatIB/FIB formation efficiency, quantified as the relative activity of the insoluble CatIB/FIB-containing pellet fraction compared to the crude cell extract (set to 100%), the initial rate activity (k_cat_; µmol_Product_ s^−1^, per subunit) of the lyophilized CatIB preparation, activity retention compared to the soluble enzyme, the relative protein and lipid content based on the initial weight of the lyophilizate and the yield of CatIBs obtained from 100 g wet *E. coli* cells

Construct	CatIB/FIB formation efficiency [%]	Activity k_cat_ [s^−1^]	Residual activity [%]^d^	Rel. protein content lyophilizate [%]	Yield $\frac{{g_{lyophilizate} }}{{100 g_{cells} }}$ g	Lipid content [%]
Constructs showing robust CatIB/FIB formation efficiency						
TDoT fusions						
TDoT-L-YFP	53.8 ± 7.4 (6)^b^	na	na	70.0 ± 5.3 (4)	4.9 ± 0.6 (3)	nd
TDoT-YFP	65.4 ± 4.9 (3)	na	na	69.2 ± 6.8 (2)	5.5 (1)	nd
TDoT-mCherry	31.8 ± 8.2 (4)^b^	na	na	85.7 ± 8.3 (2)	3.2 (1)	nd
TDoT-L-*Bs*LA^a^	114.1 ± 3.1 (1)	nd	nd	79 (1)	8.6 (1)	nd
TDoT-L-*At*HNL^a^	76.4 ± 3.5 (1)	4.3 ± 0.2 (1)	11.1	85 (1)	7.3 (1)	nd
TDoT-L-*Ec*MenD^a^	90.3 ± 0.2 (1)	nd	nd	93 (1)	12.2 (1)	nd
TDoT-L-*R*ADH	87.5 ± 3.2 (4)^b^	0.054 ± 0.008 (3)^b^	2.0^b^	84.6 ± 3.9 (3)^b^	9.7 ± 1.7 (4)^b^	14.3 ± 0.3 (1)
TDoT-L-*Pf*BAL	87.7 ± 6.8 (4)^b^	0.77 ± 0.12 (4)^b,c^	1.0^b,c^	71.9 ± 4.5 (4)^b,c^	8.8 ± 1.0 (8)^b^	16.4 ± 1.0 (1)^c^
TDoT-*Ec*LDC	nd	6.2*10^−7^ (1)	nd	nd	nd	nd
*Ec*LDC-L-TDoT	nd	0.71 (1)^e^	nd	67.9 ± 5.9 (3)^e^	12.4 ± 3.0 (3)	12.9 ± 3.2 (1)
3HAMP fusions						
3HAMP-L-*R*ADH	75.4 ± 3.7 (4)	0.33 ± 0.02 (3)	12.0	50.9 ± 7.6 (3)	3.8 ± 0.5 (3)	30.6 ± 8.3 (1)
3HAMP-L-*Pf*BAL	75.8 ± 8.0 (5)	13.9 ± 2.9 (3)^c^	18.1^c^	33.8 ± 5.2 (3)^c^	3.3 ± 0.5 (4)	30.1 ± 4.7 (1)^c^
3HAMP-L-*Lb*ADH	67.0 ± 21.7 (3)	0.60 ± 0.20 (3)	1.0	54.6 ± 8.0 (3)	8.1 ± 1.3 (3)	34.7 ± 1.36 (1)
3HAMP-L-*Pp*BFD	61.3 ± 35.4 (3)	23.4 ± 6.1 (4)	10.3	35.5 ± 6.7 (4)	6.6 ± 1.4 (3)	27.9 ± 3.7 (1)
*Ec*LDC-L-3HAMP	nd	0.80 (1)	nd	56.5 ± 6.5 (2)	7.5 ± 6.5 (4)	17.7 ± 0.6 (1)
Constructs showing low CatIB/FIB formation efficiency						
TDoT-L-mCherry	3.5 ± 1.9 (3)	na	na	15.8 ± 0.5 (1)	2.8 (1)	nd
TDoT-L-*Lb*ADH	5.4 ± 5.9 (3)	3.63 ± 0.90 (3)	5.8	43.4 ± 5.5 (3)	2.5 ± 0.4 (3)	25.2 ± 0.73 (1)
TDoT-L-*Pp*BFD	1.2 ± 0.6 (3)	9.2 ± 4.7 (4)	4.1	26.9 ± 4.1 (4)	1.6 ± 0.7 (3)	19.1 ± 0.8 (1)
3HAMP-L-YFP	6.3 ± 3.2 (4)	na	na	49.0 ± 5.7 (3)	5.4 ± 1.0 (3)	nd
3HAMP-L-mCherry	5.5 ± 0.2 (5)	na	na	36.4 ± 4.1 (4)	3.0 ± 0.9 (4)	nd

^a^Data taken from [9]

^b^Data taken from [25]

^c^Data taken from [32]

^d^Residual activity (k_ca_, µmol product, per subunit) relative to the activity of the corresponding soluble purified enzyme: (*R*ADH: k_cat_ = 2.76 ± 0.04 s^−1^; *Pf*BAL: k_cat_ = 76.7 ± 2.3 s^−1^; *Lb*ADH: k_cat_ = 62.2 ± 6.7 s^−1^; *Pp*BFD: k_cat_ = 226 ± 40 s^−1^)

^e^Data given in or derived from [10]. Numbers in brackets refer to the numbers of the biological replicates that were used to obtain error estimates. na: not applicable; nd: not determined

#### Fusion to a different coiled-coil domain

**Fig. 3:**
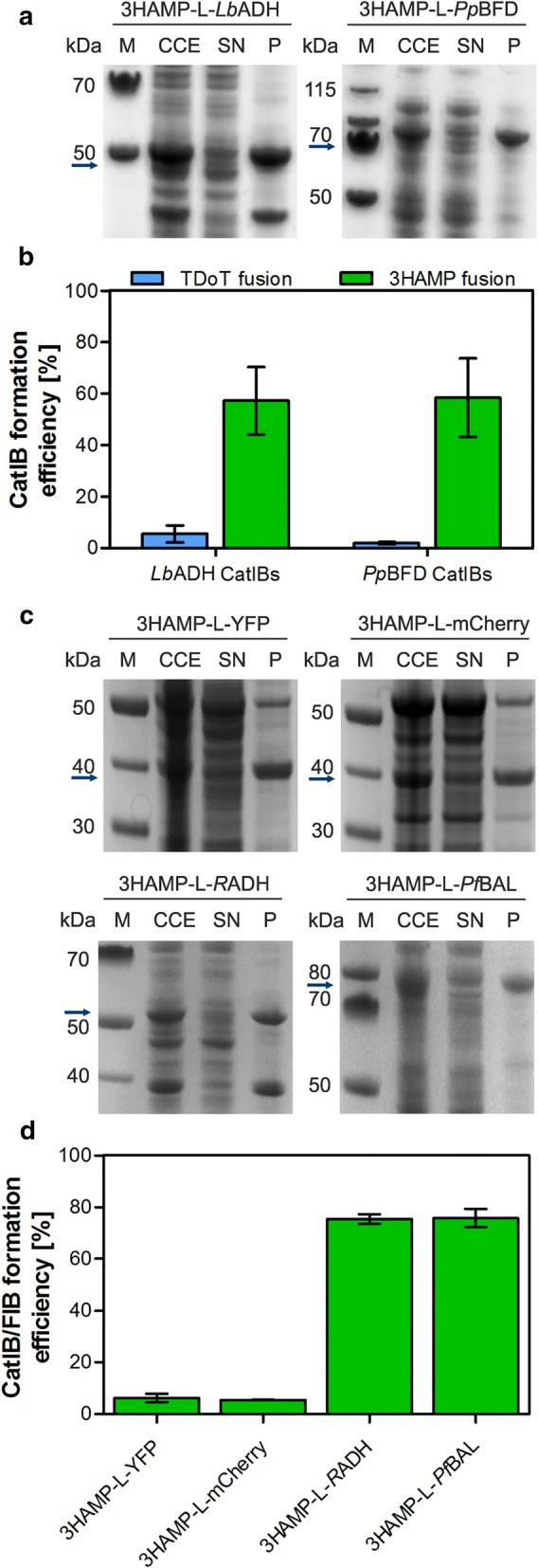
Optimization of the CatIB strategy by variation of coiled-coil domains using 3HAMP instead of TDoT fusions. CatIB formation was evaluated by **a** SDS-PAGE analysis and **b** CatIB formation efficiency for 3HAMP-L-*Lb*ADH- and 3HAMP-L-*Pp*BFD (green bars) compared to TDoT-L-*Lb*ADH and TDoT-L-*Pp*BFD (blue bars). Panels c) and d) contain the equivalent data for 3HAMP-L-YFP, 3HAMP-L-mCherry, 3HAMP-L-*Pf*BAL and 3HAMP-L-*R*ADH. After cell disruption, the crude cell extract (CCE) was separated by centrifugation into the soluble protein containing supernatant (SN) and the insoluble IB containing pellet (P) fraction. Sample preparation for SDS-PAGE analysis and the determination of the CatIB/FIB formation efficiency was carried out as described in Fig. 1. **a**, **c** SDS-PAGE analysis of the respective protein fractions: CCE, SN, and P. The molecular mass of the target fusion proteins is indicated by arrows (3HAMP-L-*Lb*ADH: 47.1 kDa; 3HAMP-L-*Pp*BFD: 77.0 kDa, 3HAMP-L-YFP: 47.4 kDa, 3HAMP-L-mCherry: 47.1 kDa, 3HAMP-L-*R*ADH: 47.1 kDa and 3HAMP-L-*Pf*BAL: 79.3 kDa). **b**, **d** CatIB/FIB formation efficiency determined as described in Fig. 1. The complete datasets illustrating the distribution of activity in the CCE, SN and P fractions can be found in Additional file 1: Figure S7. Initial rate activities were measured as described in Fig. 1

To improve the CatIB/FIB formation efficiency, the exchange of the TDoT-domain by another coiled-coil domain was considered as further optimization option. As an alternative to TDoT, the 3HAMP-domain [HAMP: histidine kinases, adenylyl cyclases, methyl-accepting chemotaxis proteins (MCPs), and phosphatases], which is part of the soluble oxygen sensor Aer2 of *P. aeruginosa* [54], was selected. The 3HAMP domain was chosen because of its larger size (172 amino acids) compared to the rather short TDoT coiled-coil domain (52 amino acids), with the rationale in mind that for larger target proteins larger coiled-coils might be needed to facilitate efficient CatIB/FIB formation. Therefore, as the next logical optimization step, we generated fusion proteins for *Lb*ADH and *Pp*BFD, which instead of TDoT were fused to the 3HAMP domain. As in case of our initial fusion strategy and in light of the above described structure analyses (Additional file 1: Figure S4; see chapter “*C*-*terminal TDoT*-*domain fusion*”), the 3HAMP domain was fused to the N-terminus of the respective target enzyme, resulting in the constructs 3HAMP-L-*Lb*ADH and 3HAMP-L-*Pp*BFD. Interestingly, for both target enzymes N-terminal 3HAMP-fusion drastically increased the CatIB-formation efficiency, as evidenced by both SDS-PAGE analysis (Fig. 3a; compare to TDoT-L-*Lb*ADH and TDoT-L-*Pp*BFD in Fig. 1a) and activity measurements of the CatIB-containing pellet fraction after fractionation of the corresponding crude cell extracts (Fig. 3b). Compared to the corresponding TDoT fusions (see also Fig. 1b), the CatIB-formation efficiency was increased 12- and 51-fold for 3HAMP-L-*Lb*ADH and 3HAMP-L-*Pp*BFD, respectively.

Prompted by these results, we also generated 3HAMP fusions of the remaining target proteins and quantified CatIB formation efficiency (Fig. 3c, d). While for YFP and mCherry the FIB formation efficiency was low, i.e. compared to the corresponding best performing TDoT construct (Fig. 2), clear CatIB formation was observed for 3HAMP-L-*Pf*BAL and 3HAMP-L-*R*ADH (Fig. 3c, d). In conclusion, the 3HAMP domain apparently can replace TDoT as a tag to induce CatIB/FIB formation and appears to be a valid alternative for difficult targets, for which the TDoT fusion approach fails.

#### Comparative characterization of TDoT and 3HAMP CatIBs/FIBs

As shown above, we were able to successfully produce CatIBs/FIBs for all of the seven tested target enzymes/proteins by optimizing our initial TDoT fusion strategy. To elucidate potential differences between CatIBs/FIBs produced by TDoT and 3HAMP fusion, we characterized all obtained CatIBs and FIBs with regard to yield (g_lyophilizate_ per 100 g wet *E.* *coli* cells), composition (relative protein and lipid content), specific activity (k_cat_), and residual activity compared to the respective soluble purified target enzymes, where possible. The corresponding data is summarized in Table 1 (see also Additional file 1: Figure S8 for details). Some of this data has been presented before, e.g. as part of CatIB application studies [10, 25, 32]. For comparison we also included the respective values (if available) from our first CatIB study, in which we demonstrated CatIB formation by TDoT fusion to the lipase A from *Bacillus subtilis* (*Bs*LA), a hydroxynitrile lyase from *Arabidopsis thaliana* (*At*HNL), and the thiamine-diphosphate (ThDP)-dependent enzyme MenD (2-succinyl-5-enol-pyruvyl-6-hydroxy-3-cyclohexene-1-carboxylate synthase) from *E.* *coli* (*Ec*MenD) [9].

To provide a better overview, we grouped the different constructs into two categories (i) TDoT and 3HAMP fusion constructs showing robust CatIB/FIB formation and (ii) constructs that only showed low CatIB/FIB formation efficiency (< 10%), irrespective of whether they were fused with TDoT or 3HAMP. The latter category contained constructs of the initial round of experiments, where the TDoT domain was fused N-terminally to the target protein/enzyme (TDoT-L-mCherry, TDoT-L-*Lb*ADH, TDoT-L-*Pp*BFD; see chapter “*Formation of CatIBs/FIBs by N*-*terminal TDoT fusion*”) as well as constructs fused with the 3HAMP domain (3HAMP-L-YFP and 3HAMP-L-mCherry; see chapter “*Fusion to a different coiled*-*coil domain*”). Compared to the constructs with robust CatIB/FIB formation efficiency those preparations showed low to moderate protein content (16–49%) and lyophilizate yields (1.6–5.4 g lyophilizate per 100 g wet cells) as well as low lipid content (19–25%). In terms of yield and composition those values likely derive from cellular constituents, which remain in the insoluble pellet after cell lysis and centrifugation, i.e. non-lysed cells, cell debris, membrane proteins, and membrane lipids. Surprisingly, the CatIB preparations of TDoT-L-*Lb*ADH and TDoT-L-*Pp*BFD, for which we only observe low CatIB formation efficiency (1.2–5.4% of the overall crude cell extract activity), still showed activities that correspond to 5.8% (TDoT-L-*Lb*ADH) and 4.1% (TDoT-L-*Pp*BFD) of the activity of the corresponding soluble purified enzymes. Several explanations could account for this phenomenon. First, the observed activities result from intact non-lysed cells, containing the respective soluble produced fusion protein, which would require that the substrates used for the activity assays can be taken up by these cells. Likewise, those intact cells could become (partially) lysed during lyophilization of the washed pellet, which would result in the release of the soluble produced fusion protein and hence could account for the observed activity. Secondly, those constructs might indeed form intracellular CatIBs, which, however, disintegrate or are solubilized during the washing step of the CatIB preparation procedure. The latter hypothesis should be observable by SDS-PAGE analyses, i.e. by the appearance of target fusion protein bands in the soluble wash fractions retrieved during the CatIB preparation procedure. Indeed, compared to TDoT-L-*Pf*BAL (Additional file 1: Figure S9c), increased solubilization/leakage of the fusion proteins is observed during the preparation of the TDoT-L-*Lb*ADH and TDoT-L-*Pp*BFD CatIBs (Additional file 1: Figure S9a and b).

Among the constructs showing robust CatIB/FIB formation, all CatIBs/FIBs produced by TDoT fusion, with the exception of the FPs, which showed FIB formation efficiencies between approx. 32% (TDoT-mCherry) and 65% (TDoT-YFP), showed higher CatIB/FIB formation efficiencies [between 76% (TDoT-L-*At*HNL) and 114% (TDoT-L-*Bs*LA)] compared to the 3HAMP fusions [between 61% (3HAMP-L-*Pp*BFD) and 76% (3HAMP-L-*Pf*BAL)]. Here, either less efficient CatIB/FIB formation or partial solubilization/leakage of the fusion protein during the CatIB/FIB preparation procedure might be potential causes. The latter is supported by SDS-PAGE analysis (Additional file 1: Figure S10), where for both 3HAMP-L-*Lb*ADH as well as 3HAMP-L-*Pp*BFD increased leakage/solubilization is observed during the washing steps performed for CatIB/FIB preparation (Additional file 1: Figure S10, compare to TDoT-*L*-*Pf*BAL, Additional file 1: Figure S9c).

Interestingly, in terms of activity (expressed as k_cat_ to account for the differences in molecular mass between the TDoT and 3HAMP fusions and the respective soluble purified enzymes) and residual activity (compared to the respective soluble enzyme), the 3HAMP CatIBs generally seem to outperform the TDoT CatIBs. With the exception of TDoT-L-*At*HNL the TDoT CatIBs showed residual activities of 1–2% of the respective soluble purified enzyme, while the 3HAMP CatIBs possessed residual activities between 10% (3HAMP-L-*Pp*BFD) and 18% (3HAMP-L-*Pf*BAL). For example, the direct comparison between equivalent TDoT and 3HAMP fusions revealed a 6- and 18-fold increase in k_cat_ and residual activity for the 3HAMP-*R*ADH and 3HAMP-*Pf*BAL CatIBs, respectively (Table 1).

The observed differences in activity between TDoT and 3HAMP CatIBs are also manifested in differences in CatIB/FIB composition. Here, the relative protein content of the respective lyophilizates was higher for the TDoT CatIBs/FIBs (between 66% [*Ec*LDC-L-TDoT) and 93% (TDoT-L-*Ec*MenD)] compared to the corresponding 3HAMP CatIBs/FIBs [between 34% (3HAMP-L-*Pf*BAL) and 57% (*Ec*LDC-L-3HAMP)]. The lower protein content of the 3HAMP CatIBs/FIBs, however, was accompanied by increased lipid content (approx. 2-fold higher than for the tested TDoT CatIBs/FIBs).

With the exception of the FPs, in terms of yield we routinely obtain 7.3–12.2 g of CatIB lyophilizate per 100 g wet cells of TDoT CatIBs, while for the 3HAMP CatIBs somewhat lower yields of 3.3–8.1 g of CatIB lyophilizate per 100 g wet cells were obtained.

In conclusion, CatIBs/FIBs derived from TDoT or 3HAMP fusion appear to possess different characteristics. Most interestingly the here described 3HAMP CatIBs showed much higher residual activities than the TDoT-derived CatIBs, which would be advantageous for application. This might be related to a less dense packing of the 3HAMP CatIBs, which would enable better substrate access and could result in higher activities. This hypothesis is supported by the observation that 3HAMP CatIBs more easily disintegrate during CatIB preparation (vide supra, see Additional file 1: Figure S10). As a carrier-free immobilization method, CatIBs can be best compared to cross-linked enzyme aggregates (CLEAs), which showed residual activities of 6–100% based on the initial activity of the enzyme preparation (usually crude cell extracts) before immobilization [55–63]. In the case of CatIBs, as in situ produced immobilizates, we cannot determine the initial total activity before the immobilization process but can only refer to k_cat_ of the purified soluble enzyme. Although a direct comparison of residual activities is not possible, it can be concluded that 3HAMP CatIBs possess residual activities that are at least comparable to certain CLEA preparations. However, compared to CLEAs, CatIBs can be produced more easily and more straightforward involving only cell lysis, centrifugation, and washing steps, i.e. not requiring tedious and expensive enzyme purification, precipitation, and/or cross-linking.

#### Morphology of the CatIBs

The distinct characteristics observed here for the TDoT and 3HAMP CatIBs/FIBs hint at distinct molecular differences, which might be observable as different CatIB/FIB morphologies. We therefore comparatively investigated the morphology of the different CatIBs/FIBs by conventional (fluorescence) microscopy and scanning electron microscopy (SEM). Hereby, IBs are known to form dense refractive particles at the cell poles in *E.* *coli*, which can be observed by conventional microscopy [64]. Previous SEM studies of isolated IBs revealed round or barrel like shapes with a size between 300 nm and 1 µm [9, 30, 65, 66].

**Fig. 4:**
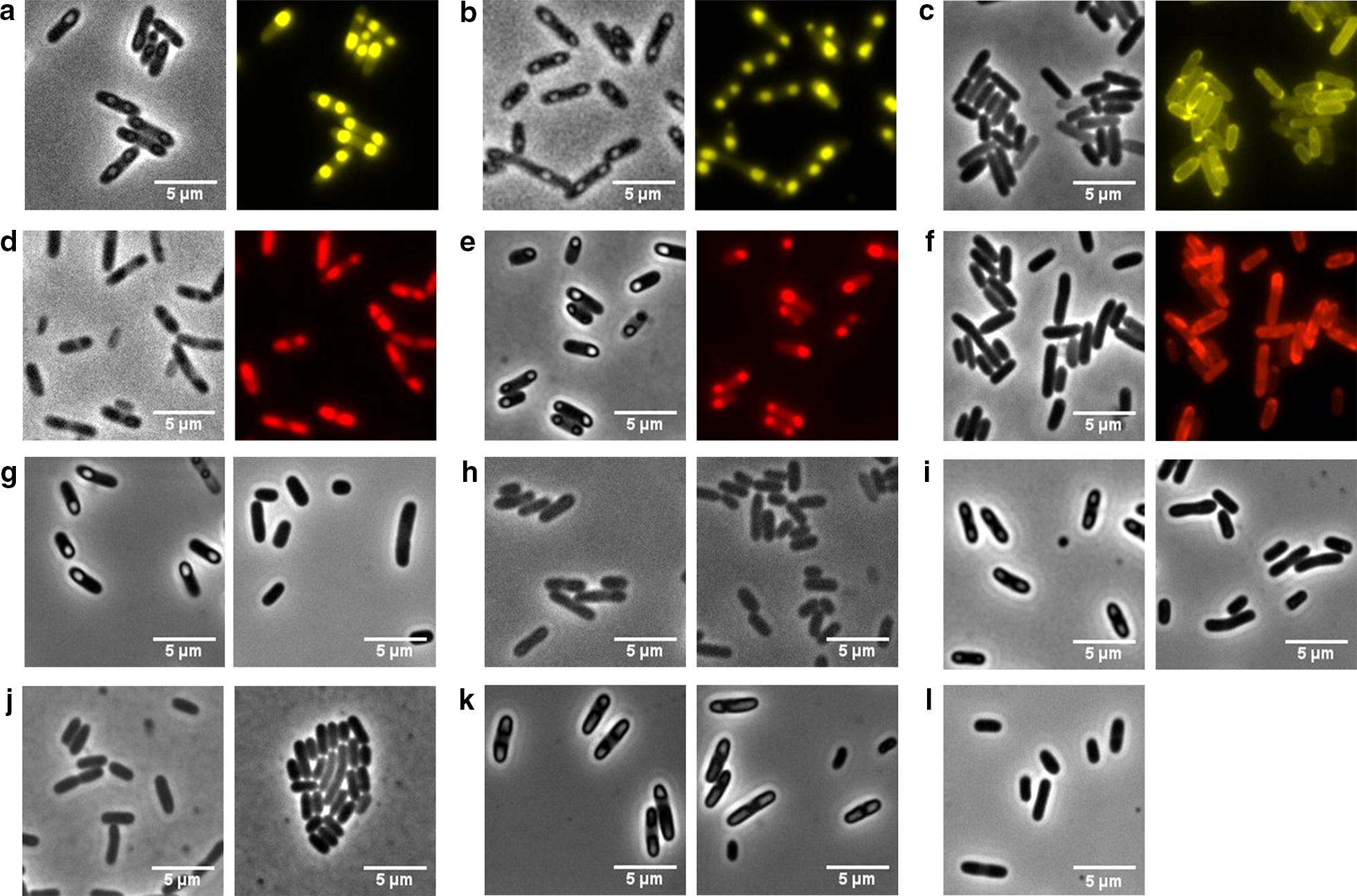
Microscopy images illustrating CatIB/FIB formation in *E.* *coli*. **a**–**f** Phase-contrast and fluorescence images of *E. coli* cells expressing **a** TDoT-L-YFP, **b** TDoT-YFP, **c** 3HAMP-L-YFP, **d** TDoT-L-mCherry, **e** TDoT-mCherry, and **f** 3HAMP-mCherry. **g**–**k** phase-contrast images of TDoT fusion (left) and 3HAMP fusion (right) expressing *E.* *coli* cells containing **g** *R*ADH, **h** *Lb*ADH, **i** *Pf*BAL, **j** *Pp*BFD, **k** *Ec*LDC (here, the coiled-coil domain is fused C-terminally), and **l** *E. coli* BL21(DE3) with empty pET-28a vector. All strains were grown under standard growth conditions as described in “Methods” section

As a first step, microscopic images of *E.* *coli* cells after production of different CatIBs/FIBs were taken (Fig. 4). Phase-contrast images were acquired for all preparations and fluorescence detection was used to directly visualize FIB formation for the YFP and mCherry FIBs (Fig. 4a–f); the latter providing additional insight into the localization and morphology of the resulting IB particles. Therefore, we first focused on the different FIB producing constructs. For all TDoT-fusions of YFP and mCherry (with or without the linker region), defined particles were visible at the cell poles, in both phase-contrast and fluorescence images. Interestingly, the construct TDoT-L-mCherry, which showed only low FIB formation efficiency (Fig. 1; Table 1), still shows intracellular FIB formation (Fig. 4d), indicating that the particles are less stable/compact and thus disintegrate more or less completely during cell lysis or later CatIB preparation steps. The same, although to a lesser degree, might be the case for TDoT-L-YFP, as also lower than average FIB formation efficiencies were observed here (Fig. 1, Table 1). In contrast, the 3HAMP-FIBs of YFP and mCherry show no distinct cell-pole localized IB particles in phase-contrast. The corresponding fluorescence images, however, reveal that the fusion proteins are partly distributed throughout the cytoplasm and are partly membrane associated (Fig. 4c, f). In a few cells, less well-defined bright fluorescent spots are found at the cell poles. This is in accordance to the low FIB formation efficiency observed for 3HAMP-L-YFP and 3HAMP-L-mCherry (Fig. 3), and indicates that, indeed, the 3HAMP-derived CatIBs/FIBs might possess a different morphology.

To address this issue, we next acquired phase-contrast images for the remaining 3HAMP and TDoT fusion constructs (Fig. 4g–k). Here, only the TDoT-fusion of *R*ADH (g), *Pf*BAL (i), and *Ec*LDC (k) as well as the 3HAMP-fusion of *Ec*LDC (k), which also showed robust CatIB formation efficiencies (Fig. 1, Table 1), gave visible CatIB formation. With the exception of the *Ec*LDC-L-3HAMP fusion, which clearly showed intracellular IB formation, all 3HAMP fusions did not show distinct IB particles in the corresponding phase-contrast images.

At the first glance, this appears contradictory to the robust CatIB formation efficiency and the high specific activity (Table 1) observed for e.g. 3HAMP-L-*R*ADH and 3HAMP-L-*Pf*BAL (Fig. 3, Table 1). In principle, two explanations could account for this discrepancy. First, although unlikely, the respective 3HAMP CatIBs are not formed inside the cell and only aggregate into particles after cell disruption. Secondly, the particles are formed within the cell but possess a less dense and more diffuse structure, so that they are not detectable as refractive particles in phase-contrast images. The observed membrane association and the presence of bright fluorescent spots at the cell poles of the 3HAMP mCherry and YFP fusions (Fig. 4c, f) would support the latter possibility. A more diffuse, less densely packed structure would also account for the higher activities observed for the 3HAMP CatIBs, as such particles would enable better substrate accessibility. Likewise, partial membrane association would also explain the increased lipid content of the 3HAMP CatIBs, as membrane lipids might become co-purified together with the CatIBs.

**Fig. 5:**
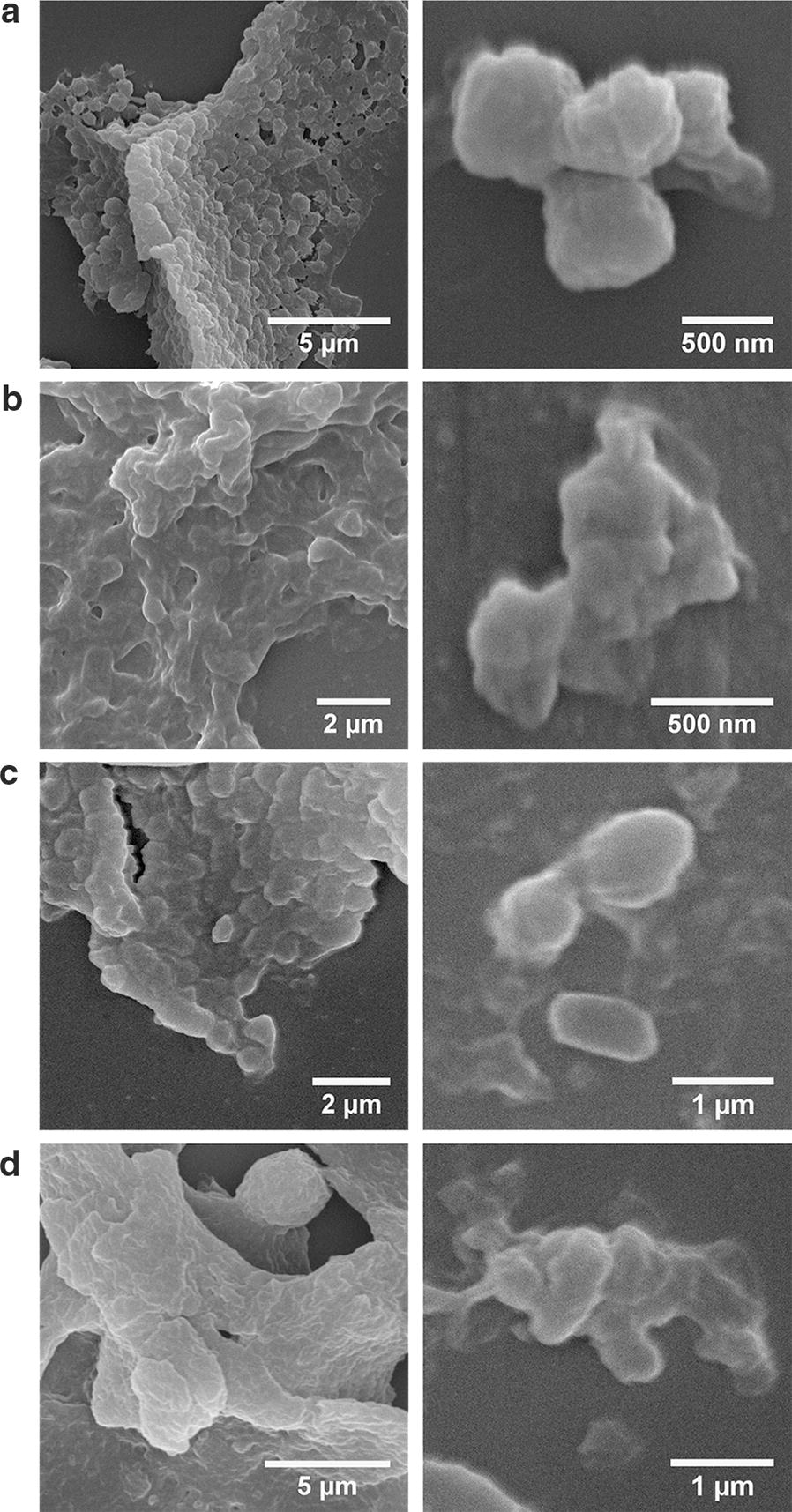
SEM images of **a** TDoT-YFP, **b** 3HAMP-L-YFP, **c** TDoT-L-*Pf*BAL, and **d** 3HAMP-L-*Pf*BAL CatIBs/FIBs at different magnification. Overview images are shown on the left and detailed images at higher magnification illustrating the smallest particles found in the corresponding preparations are shown on the right. SEM samples were prepared from the final lyophilized CatIB/FIB preparations. Samples were prepared and SEM images were acquired as described in “Methods” section

Further, more detailed insight into those morphological features might be gained by scanning SEM. Therefore, we exemplarily acquired SEM images for a set of TDoT and 3HAMP CatIBs (Fig. 5).

As expected, the TDoT-YFP and the TDoT-L-*Pf*BAL CatIBs form classical IBs with round or barrel-like shapes and a size between 500 nm and about 1 µm (Fig. 5a, c). Interestingly, the structures of the corresponding 3HAMP CatIBs appear less well ordered, forming sheets of micrometer-sized flakes, which, however, appear to consist of smaller substructures (Fig. 5b, d).

In conclusion, the TDoT and 3HAMP CatIBs, which possess different characteristics such as residual activity and composition, also show clearly distinct morphology. Moreover, the more diffuse, less densely packed structure of the 3HAMP CatIBs could account for the improved activity compared to the compact, well ordered TDoT CatIBs. To the best of our knowledge this morphological distinction has not been observed before.

#### Relationship between target sequence, structure, and CatIB formation

The fact that fusing the TDoT coiled-coil domain to certain target proteins resulted in low CatIB/FIB formation efficiencies (Fig. 1, Table 1), while others showed robust aggregate formation (although with variable efficacy), indicates that certain sequence- or structural-features are a prerequisite for CatIB/FIB formation and/or determine the efficiency of the aggregation process. This rationale includes the observation that certain CatIBs/FIBs appear to more easily disintegrate during CatIB/FIB preparation, as this phenomenon likewise results in lower apparent CatIB/FIB formation efficiencies.

**Fig. 6:**
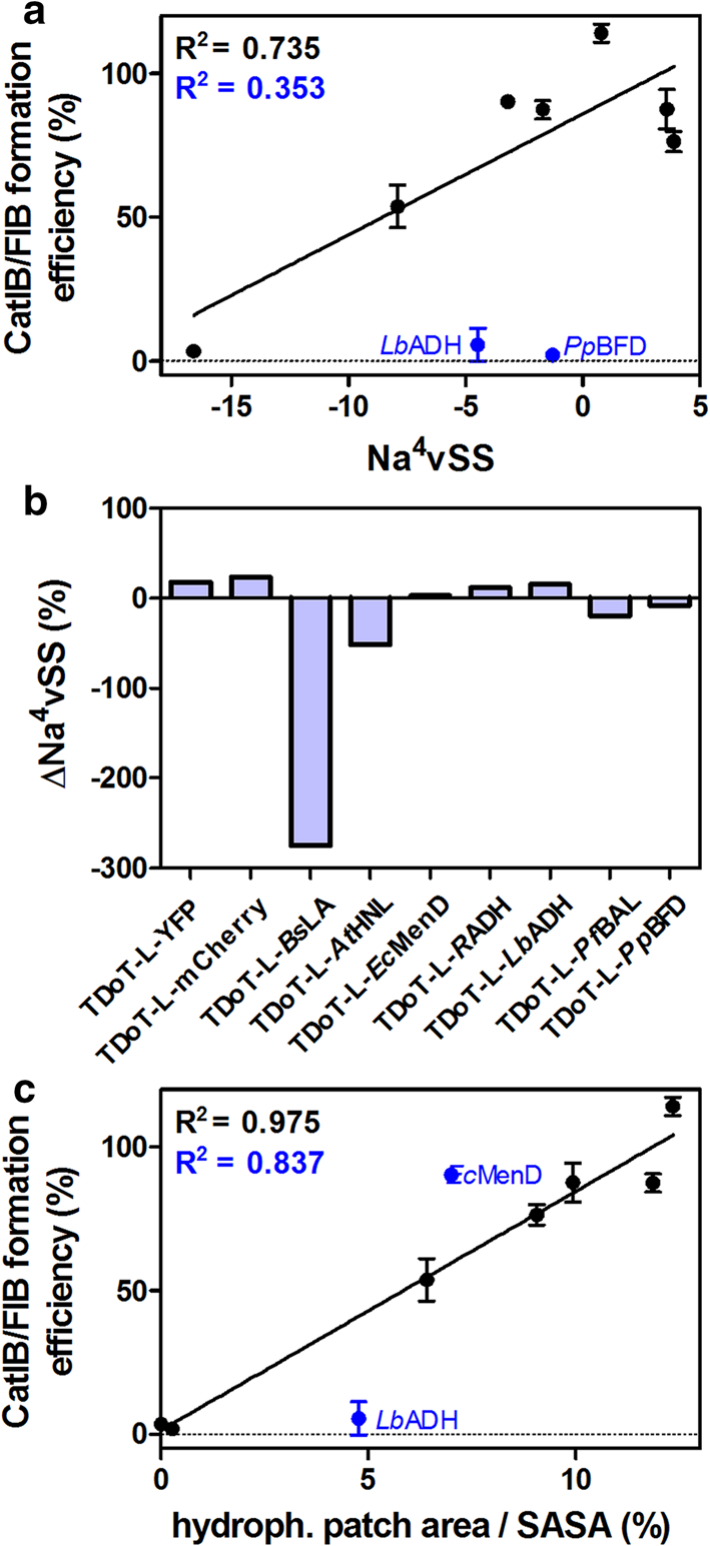
Computational analysis of the **a**, **b** sequence-based and **c** structural determinants of CatIB/FIB formation analyzed based on the TDoT dataset. **a** Sequence-based aggregation propensities were inferred using the AGGRESCAN webserver [72] and the average aggregation-propensity values per amino acid (a^4^v) normalized to a 100-residue protein (Na^4^vSS) were used as indicator for aggregation. Low (negative) Na^4^vSS are an indicator for low aggregation propensity as for example demonstrated for intrinsically disordered proteins (IDPs) [72]. **b** The relative change of the Na^4^vSS value due to addition of the TDoT domain $\left( {{\Delta Na}^{4} \text{vSS} = \left( {\frac{{\left( {\text{Na}^{4} \text{vSS}_{{\text{fusion}}} - \varvec{ }\text{Na}^{4} \text{vSS}_{{\text{target}}} } \right)}}{{\left| {\text{Na}^{4} \text{vSS}_{{\text{target}}} } \right|}}} \right) \times 100} \right)$ has in the past been used for the computation of the effects of point mutations on aggregation [72]. Positive values suggest increased and negative values decreased aggregation due to addition of the TDoT domain. **c** The presence/absence of large hydrophobic surface patches for the corresponding target protein structures was quantified using the hpatch tool implemented in Rosetta [75, 76, 94]. Surface areas were quantified using Pymol 1.7.0.0 (Schrödinger, LCC, New York, NY, USA). In a and c CatIB-formation was plotted as the relative activity in the insoluble fraction (Additional file 1: Table S1). Coefficient of determination (R^2^) values are always given excluding the blue-highlighted outliers (black) and including the outliers (blue)

We therefore initially analyzed the here employed target proteins as well as the corresponding TDoT fusions for their propensity to aggregate using sequenced-based predictions, as recent studies have indicated that the propensity for IB formation is linked to certain aggregation-prone sequence stretches [11, 67–69]. Hereby, the aggregation propensity of unfolded polypeptide chains appears to be correlated to physicochemical properties like hydrophobicity, secondary structure propensity and charge [70], which can be inferred from the amino acid sequence of both the target protein and the fusion [71]. We here used AGGRESCAN, one of the more widely employed tools for the prediction of aggregation hot spots [72]. In Fig. 6a, the CatIB/FIB formation efficiency (Table 1) of all TDoT fusions was plotted against the AGGRESCAN-derived Na^4^vSS score (for further explanations see “Methods”; Additional file 1: Table S1). With the exception of *Lb*ADH and the *Pp*BFD (which both did not form classical, compact CatIBs when fused to the TDoT domain; Fig. 4h, j), there seems to be a weak linear relationship between the Na^4^vSS values of the target proteins and the CatIB-formation propensity (outliers were *Lb*ADH and *Pp*BFD; R^2^ = 0.735 when excluding outliers and R^2^ = 0.353 when including outliers). Here, target proteins that yield low Na^4^vSS values (mCherry, YFP; Additional file 1: Table S1) also yield lower activities/fluorescence in the insoluble fraction. Such low aggregation propensities (i.e. high negative Na^4^vSS values) have been for example inferred for intrinsically disordered proteins (IDPs) [72], which are generally very resistant to aggregation and often remain soluble even after boiling [73, 74]. In contrast, the majority of the here employed target proteins showing robust CatIB-formation yield Na^4^vSS values between − 5 and + 5 (Additional file 1: Table S1). Thus, they show aggregation propensities well within the range reported for globular, soluble, and IB-forming polypeptides [72].

We next tried to address the influence of fusing the TDoT coiled-coil domain to any given target by calculating the relative change of the Na^4^vSS value due to addition of the TDoT domain (ΔNa^4^vSS) (Fig. 6b; Additional file 1: Table S1). Here, no clear trend was observed. On the contrary, while some of the targets that show little aggregation or no classical compact CatIBs (mCherry, *Lb*ADH, *Pp*BFD, YFP) exhibit low positive or low negative ΔNa^4^vSS values, the *Bs*LA and *At*HNL fusions, which display robust CatIB formation, show the most prominent (− 275% and − 51%) change in Na^4^vSS by addition of the TDoT domain. This suggests that the TDoT fusion should increase their solubility, which however was not observed experimentally (Additional file 1: Table S1). In conclusion sequence-based predictions can be used in a first approximation to predict the aggregation propensity of a given target protein, however, the consequences of TDoT fusion (i.e. the efficacy of the resulting CatIB-formation process) cannot be directly inferred or understood only based on those predictions.

Since CatIBs/FIBs, in contrast to conventional IBs, retain a certain degree of activity, it seems reasonable to assume that the corresponding enzymes/proteins retain their native (quaternary) structure in CatIBs/FIBs (at least to some extend). Therefore, it seems likely that in CatIBs/FIBs aggregation does not solely occur from the unfolded state (as the previous sequence-based predictions assume) but also involves the aggregation or co-aggregation of already folded (i.e. native) protein species. Hereby, the presence/absence of hydrophobic surface patches on a given target could determine the efficiency of aggregation. To address this issue, we evaluated the available target protein structures for the presence of large hydrophobic surface patches by using the Rosetta protein design software (see “Methods” for details; Additional file 1: Figure S11, Table S2) [75, 76]. For five out of nine of the target proteins, the plot of the CatIB/FIB formation efficiency against the percentage of the hydrophobic patch area on the overall solvent accessible surface area (SASA) yielded a good linear relation (excluding outliers: R^2^ = 0.995; including *Ec*MenD, *R*ADH, YFP, and *Lb*ADH: R^2^ = 0.286) (Additional file 1: Figure S12). When we consider the presence of alternative oligomeric assemblies (inferred by using the PISA webserver; see “Methods”; Additional file 1: Table S2), the correlation is significantly improved for *R*ADH and YFP (excluding outliers: R^2^ = 0.975; including *Ec*MenD and *Lb*ADH: R^2^ = 0.837) (Fig. 6c). Here, it appears that the presence of large hydrophobic surface patches clearly relates to the efficacy of CatIB formation, thus providing a structural rationale, why certain highly soluble proteins like mCherry fail to form insoluble FIBs or form only FIBs that disintegrate during cell lysis or are solubilized during CatIB/FIB preparation.

## Conclusions

The generation of catalytically-active inclusion bodies (CatIBs) represents a recently developed, promising strategy for the solely biological production of carrier-free enzyme immobilizates. This strategy relies on the molecular biological fusion of a coiled-coil domain to target enzymes/proteins to induce the formation of intracellular aggregates (inclusion bodies, IBs) which retain a certain degree of activity. While this strategy has already been proven successful in multiple cases, the efficiency, the potential for optimization, and important CatIB properties like yield, activity, and morphology have not been investigated systematically. In this contribution, different optimization strategies, like linker deletion, C- versus N-terminal fusion, and the fusion of alternative aggregation-inducing tags have been evaluated. While linker deletion and C-terminal instead of N-terminal fusion successfully yielded CatIBs/FIBs for certain target proteins for which our initial N-terminal fusion strategy failed, the use of the 3HAMP coiled-coil domain as alternative aggregation-inducing tag resulted in CatIBs with superior activity and altered composition. Using conventional microscopy and scanning electron microscopy, we provide evidence for the distinct morphology of 3HAMP-derived CatIBs. The latter appears moreover to be linked to their superior performance. Last but not least, we demonstrated that CatIB formation efficiency can be correlated to the solvent-accessible hydrophobic surface area of the target enzyme, providing a structure-based rationale for our strategy and opening up the possibility to predict its efficiency for any given target protein. In conclusion, we here provide evidence for the general applicability, predictability, and flexibility of the CatIB immobilization strategy, highlighting its application potential for synthetic chemistry and industry.

## Methods

### Reagents and chemicals

Chemicals were purchased from Sigma-Aldrich, Fluka, Roth, KMF, Biosolve, Alfa Aesar, AppliChem, and Merck. Enzymes for molecular biology were purchased from Thermo Scientific (Waltham, MA, USA). Enantiopure (*R*)-(3,3‘,5,5‘)-tetramethoxy benzoin (TMBZ) for the calibration of HPLC analysis was taken from a stock prepared as described elsewhere [10, 25, 77].

### Construction of expression plasmids

The general design strategy for the construction of the respective TDoT gene fusions has been described before [9] (Additional file 1: Figure S5a). If not stated otherwise, all gene fusions consisted of gene fragments coding for a coiled-coil domain (here TDoT or 3HAMP), a linker polypeptide, consisting of a protease Factor Xa cleavage site and a triple (GGGS)_3_ and the respective target proteins/enzymes cloned into a pET-28a vector (Novagen, Merck KGaA, Frankfurt, Germany). As target FPs YFP (27.1 kDa), a monomeric version of the enhanced yellow fluorescent protein (eYFP) from *Aequorea victoria* was used. This YFP contains the A206K exchange for the monomerization [36] but lacks the Q69K substitution, which renders it less sensitive in the neutral pH [78, 79]. As a second FP target, the monomeric red fluorescent protein mCherry from *Discosoma striata (*26.7 kDa) [37] was chosen. As target enzymes, two alcohol dehydrogenases (*R*ADH from *Ralstonia* sp. and *Lb*ADH from *Lactobacillus brevis* [38–40]) and two ThDP-dependent enzymes [benzoylformate decarboxylase from *Pseudomonas putida* (*Pp*BFD) [43] and benzaldehyde lyase from *Pseudomonas fluorescence* (*Pf*BAL)] [46, 47] were used. To simplify the toolbox vector, the N-terminal His_6_-tag was removed from pTDoT-Linker-YFP [9], resulting in the pTDoT-L-YFP vector, as described before [25]. All in the following described constructs were based on this simplified toolbox vector and hence lacked the N-terminal His_6_ tag.

The pTDoT-YFP and pTDoT-mCherry vectors lacking the linker polypeptide, consisting of the Factor Xa protease cleavage site and the triple (GGGS)_3_ motif, were created as described before [25]. For the exchange of the coiled-coil domain, the pTDoT-L-YFP plasmid was digested with *Nde*I and *Spe*I to release the *tdot* fragment. A codon-optimized *3hamp* gene fragment, containing 5′-*Nde*I and 3′-*Spe*I restriction sites, was synthesized and supplied on a plasmid (pEX-A-3HAMP-Linker, Eurofins Genomics, Ebersberg, Germany). After restriction, the corresponding *3hamp* gene fragment was ligated into the initial plasmid, lacking the *tdot* gene fragment, to attain the p3HAMP-L-YFP vector. Genes coding for mCherry, *R*ADH, *Lb*ADH, *Pf*BAL, *Pp*BFD, and *Ec*LDC were amplified by standard PCR utilizing oligonucleotide primers containing a 5′-*BamH*I and a 3′-*Sal*I (mCherry, *R*ADH, *Lb*ADH, *Ec*LDC) or 3′-*Not*I (*Pf*BAL, *Pp*BFD) site. PCR products were digested with respective restriction endonucleases and ligated into similarly hydrolyzed pTDoT-L-YFP or p3HAMP-L-YFP. The vectors containing the TDoT-L-*R*ADH and TDoT-L-*Pf*BAL fusion as well as the vector containing the 3HAMP-L-*Pf*BAL fusion has been constructed as described in [25] and [32], respectively. The construction of the plasmid p*Ec*LDC-L-TDoT, for C-terminal fusion of TDoT to *Ec*LDC, has also been described before [10]. The plasmid containing the gene fusion encoding for the C-terminal *Ec*LDC-L-3HAMP fusion (p*Ec*LDC-L-3HAMP) was constructed similarly to the N-terminal 3HAMP-vectors by digesting p*Ec*LCD-L-TDoT with *Bam*HI and *Not*I and ligating the resulting linear DNA with a PCR amplified *3hamp* gene fragment utilizing oligonucleotide primers containing a 5′-*BamH*I and a 3′-*Not*I restriction site, originated from the pEX-A-3HAMP-Linker vector. All sequences were verified by sequencing (Seqlab GmbH, Göttingen, Germany and LGC genomics, Berlin, Germany). For information about all plasmids and oligonucleotide primers see Additional file 1.

### Production and purification of inclusion bodies (IBs)

The target gene fusions were heterologously expressed in *E.* *coli* BL21(DE3) using autoinduction medium [80] for 69 h at 15 °C as described recently [9, 10, 25]. Cell disruption was performed from a 10% (w/v) suspension in lysis buffer (50 mM sodium phosphate buffer, 100 mM NaCl, pH 8.0) with an Emulsiflex-C5 high-pressure homogenizer (Avestin Europe GmbH, Mannheim, Germany) as described before [10, 25]. To separate the IB-containing pellet from the soluble supernatant, the crude cell extract was centrifuged (30 min at 4 °C and 15,000×*g*) and frozen at − 20 °C in a freezer. The pellet was washed once with the initial volume of MilliQ water and was again centrifuged. The obtained pellet was lyophilized for 72 h from a frozen (− 80 °C) 10% (w/v) suspension in MilliQ water (Christ ALPHA 1-3 LD Plus, Martin Christ Gefriertrocknungsanlagen GmbH, Osterode, Germany). The dried CatIBs were grounded and stored as a fine powder at − 20 °C until further use [10, 25].

### Production and purification of soluble enzymes

Soluble *R*ADH, encoded on a pET-22b vector [81], was produced in *E.* *coli* BL21(DE3) according to the expression protocol used for the CatIB production. Soluble *Lb*ADH, encoded on a pET-21a vector, was produced in *E.* *coli* BL21(DE3) as described elsewhere [82, 83]. Soluble *Pf*BAL was fused to a C-terminal hexahistidine tag and was encoded on a pkk233_2 vector [84]. The protein was produced in *E.* *coli* SG 13009 according to a protocol described elsewhere [77, 84] using a 40 l Techfors fermenter (Infors AG, Bottmingen, Swiss) at 30 °C in fed-batch mode [85]. Soluble *Pp*BFD-L476Q (fused to a C-terminal hexahistidine tag) encoded on the pkk233_2 vector was produced in *E.* *coli* SG 13009 according to a protocol described elsewhere [48].

Cells were harvested, centrifuged and the remaining pellet was frozen at − 20 °C. The frozen cells were suspended in a 25% (w/v) suspension in the respective equilibration buffer used for purification. Cell disruption was performed on ice by sonication (UP200 s, Hielscher Ultrasonics GmbH, Teltow, Germany) 10-times for 1 min at an amplitude of 70% and a cycle of 0.5, followed by a 1 min break. The soluble enzyme was separated from the cell debris by centrifugation for 30 min (18,000×*g*, 4 °C).

Purification of soluble *R*ADH was performed by anion exchange chromatography according to the protocol described previously [81]. The first step included a desalting by gel filtration with a Sephadex-G25 (GE Healthcare, Little Chalfont, United Kingdom) column with 10 mM TEA-buffer (pH 7.5, 0.8 mM CaCl_2_). In the second step the desalted protein fraction was purified via anion exchanger (Q-Sepharose Fast Flow column, GE Healthcare, Little Chalfont, United Kingdom) starting with equilibration buffer (50 mM TEA, pH 7.5, 0.8 mM CaCl_2_), followed by an application of a linear NaCl-gradient up to 200 mM NaCl (50 mM TEA, pH 7.5, 0.8 mM CaCl_2_, 200 mM NaCl) within 150 min at a flow of 1 ml min^−1^. Desalting was performed again by gel filtration on a Sephadex-G25 (GE Healthcare, Little Chalfont, United Kingdom) column with 10 mM TEA-buffer (pH 7.5, 0.8 mM CaCl_2_).

Soluble *Lb*ADH was purified by anion-exchange chromatography [82, 83] by an anion exchanger (Q-Sepharose Fast Flow column, GE Healthcare, Little Chalfont, United Kingdom) starting with equilibration buffer (50 mM TEA, pH 7.2, 1 mM MgCl_2_). This was followed by an application of a linear NaCl-gradient up to 1 M NaCl (50 mM TEA, pH 7.2, 1 mM MgCl_2_, 1 M NaCl) within 150 min at a flow of 1 ml min^−1^. Desalting was performed by gel filtration on a Sephadex-G25 (GE Healthcare, Little Chalfont, United Kingdom) column with 10 mM TEA-buffer (pH 7.5, 1 mM MgCl_2_).

The soluble *Pf*BAL was purified by metal ion affinity chromatography as described earlier [46, 86]. For the purification with the Ni–NTA-Sepharose column (QIAGEN, Hilden, Germany) the following buffers were used: equilibration buffer (50 mM TEA, pH 7.5, 2.5 mM MgSO_4_, 0.5 mM ThDP, 300 mM NaCl), washing buffer (50 mM TEA, pH 7.5, 50 mM imidazole, 300 mM NaCl), elution buffer (50 mM TEA, pH 7.5, 250 mM imidazole, 300 mM NaCl). For the final desalting step with Sephadex-G25 (GE Healthcare, Little Chalfont, United Kingdom) column, 10 mM TEA-buffer (pH 7.5, 2.5 mM MgSO_4_, 0.1 mM ThDP) was employed.

The soluble *Pp*BFD-L476Q was purified by metal ion affinity chromatography as described earlier [45, 46, 48]. For the purification with Ni–NTA-Sepharose column (QIAGEN, Hilden, Germany) the following buffers were used: equilibration buffer (50 mM KPi, pH 7.0, 2.5 mM MgSO_4_, 0.1 mM ThDP), washing buffer (50 mM TEA, pH 7.0, 50 mM imidazole), elution buffer (50 mM TEA, pH 7.5, 250 mM imidazole). For the final desalting step with Sephadex-G25 (GE Healthcare, Little Chalfont, United Kingdom) column, 10 mM TEA-buffer (pH 6.5, 2.5 mM MgSO_4_, 0.1 mM ThDP) was employed.

The enzyme solutions were lyophilized (Christ ALPHA 1-3 LD Plus, Martin Christ Gefriertrocknungsanlagen GmbH, Osterode, Germany) from frozen (− 20 °C), maximal 2 mg ml^−1^ protein solutions (in the respective storage buffer) and stored at − 20 °C until further use.

### Sodium dodecyl sulfate-polyacrylamide gel electrophoresis (SDS-PAGE) and determination of protein concentration

The distribution of the recombinant fusion proteins in *E.* *coli* cell extract fractions, crude cell extract (CCE), soluble supernatant (SN), and insoluble IB-containing pellet (P), as well as the success of the IB-purification was analyzed by SDS-PAGE as described recently [10, 25]. For SDS-PAGE NuPAGE™ 4–12% Bis–Tris Protein Gels with MES SDS running buffer (50 mM MES, 50 mM TRIS, 0.1% SDS, 1 mM EDTA, pH 7.3) and PageRuler Prestained Protein ladders or PageRuler Plus Prestained Protein ladders (both: ThermoFisher Nunc, Waltham, MA, USA) were used. The total protein content in the supernatant was determined, using the Bradford assay [87]. SDS-PAGE samples of the supernatant fraction contained 10 μg protein, all other samples were prepared relative to the supernatant fractions by using the same sample volume [10, 25].

The protein content of lyophilized CatIBs was determined by the absorption at 280 nm. Therefore, lyophilized CatIBs were dissolved in 6 M guanidine hydrochloride, incubated for 30 min at 30 °C under constant shaking at 1000 rpm (Thermomixer comfort, Eppendorf, Germany), and centrifuged for 20 min at 4 °C and 16,060×*g*. The absorption of the protein solution was measured at 280 nm. The protein content was estimated using the molar extinction coefficient as calculated based on the amino acid composition using the ProtParam Tool (http://web.expasy.org/protparam [88] (Additional file 1: Table S6).

### Cell fractionation and determination of the CatIB/FIB formation efficiency

Inclusion body production was evaluated by determining the distribution of functional recombinant fusion proteins in different *E.* *coli* cell extract fractions. Therefore, the fluorescence or activity of the respective target protein was measured in all fractions: crude cell extract (CCE), supernatant (SN), and pellet (P) as described before [25]. Suitable dilutions of the CCE in lysis buffer (50 mM sodium phosphate buffer, 100 mM sodium chloride, pH 8.0) were separated into the soluble supernatant (SN) fraction and insoluble IB-containing pellet fraction (P) by centrifugation (2 min, 7697×*g*, room temperature). The P fraction was washed once with lysis buffer and was resuspended in the initial volume of lysis buffer before measuring. The fluorescence/activity in P (IBs) and SN (soluble protein) was expressed relative to the activity of the crude cell extract (set to 100%). CatIB/FIB formation efficiency was defined as the relative activity, or in case of FPs fluorescence, of the insoluble IB-containing pellet fraction.

For the fluorescent proteins YFP and mCherry distribution in different fractions [crude cell extract (CCE), soluble protein-containing supernatant (SN), and IB-containing pellet (P)] was determined by fluorescence spectroscopy, as described recently [25].

The distribution of the enzymes *R*ADH, *Lb*ADH, *Pf*BAL, and *Pp*BFD in different cell fractions was determined by continuous photometric activity assays in 10 × 4 mm quartz-glass cuvettes with a volume of 1 ml (4 mm light path in excitation) using a Fluorolog3-22 spectrofluorometer (Horiba Jobin–Yvon, Bensheim, Germany) in front-face angle according to the *Pf*BAL initial rate activity assay developed by Schwarz [77].

*R*ADH activity was measured by following the reduction of cyclohexanone to cyclohexanol (Additional file 1: Figure S13a) by detecting the consumption of the cofactor NADPH. The reaction was monitored for 90 s at 30 °C by excitation at λ_ex_ 350 nm and emission at λ_em_ 460 nm (bandwidth 1.4 nm in excitation and emission) using TEA-buffer (50 mM TEA, 0.8 mM CaCl2, pH 7.5) with 100 mM cyclohexanone, 0.2 mM NADPH, and 200 μl sample suspension in suitable dilutions. Measurements of all distributions were performed at least as four technical replicates of biological triplicates.

*Pf*BAL activity was measured using the carboligation of 3,5-dimethoxybenzaldehyde (DMBA) to (*R*)-(3,3′,5,5′)-tetramethoxy benzoin (TMBZ) (Additional file 1: Figure S13c). DMBA consumption was monitored for 90 s at 25 °C by excitation at λ_ex_ 350 nm and emission at λ_em_ 460 nm (bandwidth 1.3 nm in excitation and emission) in TEA-buffer (50 mM TEA, 0.5 mM ThDP, 2.5 mM MgSO_4_, pH 8.0) with 3 mM DMBA [in DMSO, final concentration 20% (v/v)] and 200 μl sample suspension in suitable dilutions.

The *Lb*ADH and *Pp*BFD activity distribution in different *E. coli* cell extract fractions was measured as described in the activity assays section below.

### Phase-contrast and fluorescence image acquisition

Microscopy imaging was performed as described before [25]. After cultivation of *E.* *coli* BL21(DE3) containing CatIBs/FIBs, a culture volume of 1 ml was removed and the cells were harvested by centrifugation for 2 min at 15,800×*g*. The resulting cell pellet was suspended in lysis buffer (50 mM sodium phosphate buffer, 100 mM NaCl, pH 8) to an OD_600_ of approx. 10. A volume of 1.5 μl was applied on a microscope slide with a 1% (w/v) agarose base, covered with a coverslip and placed in the microscope setup for imaging. An inverted Nikon Eclipse Ti microscope (Nicon GmbH, Düsseldorf, Germany) was used, equipped with an Apo TIRF 100× Oil DIC N objective (ALA OBJ-Heater, Ala Scientific Instruments, USA), an ANDOR Zyla CMOS camera (Andor Technology plc., Belfast, UK), and an Intensilight (Nicon GmbH, Düsseldorf, Germany) light source for fluorescence excitation, and fluorescence filters for YFP (excitation: 520/60 nm, dichroic mirror: 510 nm, emission: 540/40 nm) and mCherry (excitation: 575/15 nm, dichroic mirror: 593 nm, emission: 629/56 nm) (AHF Analysentechnik, Tübingen, Germany). The filter spectra are given in nm as peak/peak width. The dichroic mirror serves as longpass filter for wavelengths larger than the given value. Fluorescence and camera exposure was 200 ms for both filters at 25 or 12.5% lamp intensity. Analysis of cell images were performed with Fiji [89].

### Lipid content determination

For the gravimetric determination of the lipid content [90] approx. 100 mg lyophilized CatIBs were weighted and transferred into a 50 ml falcon tube. After mixing with 14 ml chloroform and 7 ml methanol, the suspension was incubated for 2 h at 60 °C and 750 rpm in a thermomixer (Thermomixer comfort, Eppendorf, Hamburg, Germany). After incubation, the complete suspension was transferred to a 50 ml separating funnel for washing with 5.6 ml 0.73% (w/v) NaCl solution. After collecting the lower organic phase, the remaining aqueous phase was extracted with 14 ml chloroform. The organic phase was pooled, dried over MgSO_4_, and concentrated by a rotating evaporator (Rotavapor R-100, Büchi Labortechnik GmbH, Essen, Germany). The remaining liquid was transferred to a glass vessel and organic solvent was removed by evaporation first under the hood and then under high vacuum (0.2 mbar) over 24 h. The lipid amount was gravimetrically determined. The lipid content was calculated based on the initial weight. All measurements were performed in three technical replicates of one biological sample.

### Activity assays

The initial rate activity of *R*ADH and *R*ADH-CatIBs was measured by using a discontinuous photometric assay in which the consumption of the cofactor NADPH was measured at 340 nm, during the enzyme-catalyzed reduction of cyclohexanone to cyclohexanol (Additional file 1: Figure S13a). The reaction was performed in a polypropylene reaction tube (2 ml safe-lock tube) in a reaction volume of 1750 µl containing 100 mM cyclohexanone and 0.4 mM NADPH in TEA-buffer (50 mM, pH 7.5, 0.8 mM CaCl_2_) which was pre-incubated at 30 °C. The reaction was started with 300–500 µg ml^−1^ *R*ADH-CatIBs or 10–20 µg ml^−1^ soluble *R*ADH (pre-incubated for 5 min at 30 °C). Reactions were performed for 5 min at 30 °C and 1000 rpm in a thermomixer (Thermomixer comfort, Eppendorf, Germany). Every minute (0–5 min) samples of 250 µl were taken and diluted 1:3 in MeOH to stop the reaction. Samples were centrifuged for 5 min (7697×*g*, room temperature) and measured in standard disposable cuvettes. The amount consumption of NADPH was quantified employing a molar extinction coefficient of ε_340nm_ = 1.975 M^−1^ cm^−1^ as determined in the reaction system.

For initial rate activity determination of *Pf*BAL-CatIBs and soluble *Pf*BAL, the carboligation of 3,5-dimethoxy benzaldehyde (DMBA) to (*R*)-(3,3‘,5,5‘)-tetramethoxy benzoin (TMBZ) (Additional file 1: Figure S13c) was followed to a conversion of 10% by a discontinuous HPLC assay. The reaction was carried out in polypropylene reaction tubes in 1 ml reaction volume comprised of 80% (v/v) TEA-buffer (50 mM, pH 7.5, 2.5 mM MgSO_4_, 0.1 mM ThDP), 20% (v/v) DMSO and 10 mM DMBA, This solution was incubated at 30 °C before the reaction was started by addition of the enzyme (0.017–0.30 mg ml^−1^ *Pf*BAL-CatIBs, 3–6 µg ml^−1^ soluble *Pf*BAL, initial protein weight) The reaction was performed for 5 min at 30 °C and 1000 rpm in a thermomixer (Thermomixer comfort, Eppendorf, Germany) under sampling (20 µl) every minute. Subsequently, the sample was diluted 1:10 with 180 µl methanol (incl. 0.1‰ (v/v) *p*-methoxy benzaldehyde as internal standard) to stop the reaction and to prepare the sample for HPLC analysis (see below).

The initial rate activity of *Ec*LDC-CatIBs was measured for the decarboxylation of 10 mM l-lysine in potassium phosphate buffer (50 mM, pH 8.0) containing 0.1 mM PLP at 30 °C and 1000 rpm by a discontinuous HPLC-based assay according to the protocol described previously [10].

*Lb*ADH and *Pp*BFD initial rate activities were measured by continuous photometric activity assays in 10 × 4 mm quartz-glass cuvettes with a volume of 1 ml (4 mm light path in excitation) using a Fluorolog3-22 spectrofluorometer (Horiba Jobin–Yvon, Bensheim, Germany) in front-face angle [77].

*Lb*ADH activity was measured for the reduction of acetophenone to 1-phenylethanol (Additional file 1: Figure S13b) under the consumption of the cofactor NADPH [83], which was detected by excitation at λ_ex_ 350 nm and emission at λ_em_ 460 nm (bandwidth 1.5 nm in excitation and emission). The reaction was started by addition of 500 μl sample suspension in suitable dilutions (protein amount of approx. 0.07–0.4 mg ml^−1^ soluble *Lb*ADH and 2–25 mg ml^−1^ *Lb*ADH-CatIBs) to the preheated TEA-buffer (50 mM pH 7.0, 0.8 mM MgCl_2_) containing 10.7 mM acetophenone, and 0.2 mM NADPH, and was followed for 90 s at 30 °C. For NADPH calibration, NADPH, in concentrations between 0.1 mM and 0.20 mM, was dissolved in TEA-buffer and measured under the same conditions.

*Pp*BFD activity was followed by a coupled two-step assay reaction beginning with *Pp*BFD-catalyzed decarboxylation of phenylglyoxylic acid (PGA) to benzaldehyde, which was followed by the reduction to benzyl alcohol by horse liver (HL-)ADH under the oxidation of NADH (Additional file 1: Figure S13d). The reaction was started by the addition of 500 μl sample suspension in suitable dilutions (protein amount of approx. 0.05–0.35 mg ml^−1^ soluble *Pp*BFD and 0.4–2.5 mg ml^−1^ *Pp*BFD-CatIBs) to the preheated reaction solution containing TEA-buffer (50 mM TEA, 0.5 mM ThDP, 2.5 mM MgSO_4_, pH 6.5) with 5 mM PGA, 0.25 mM NADH, and 0.25 U ml^−1^ HL-ADH. NADH consumption was monitored for 90 s at 30 °C by excitation at λ_ex_ 350 nm and emission at λ_em_ 460 nm (bandwidth 1.4 nm in excitation and emission). For NADPH calibration, NADPH concentrations between 0.1 mM and 0.25 mM were dissolved in TEA-buffer and measured under the same conditions.

Measurements of the initial rate activities were performed at least as three technical replicates of the respective biological triplicates. Activity was calculated as turn over number k_cat_ [s^−1^] referring to the amount of enzyme (in µmol and referring to one subunit, calculated based on the protein content) which catalyzes the formation of 1 µmol product per second from the respective substrate under the applied reaction conditions.

### HPLC analysis

For *Ec*LDC activity determinations the concentration of l-lysine and 1,5-diaminopentane (DAP) was determined as described recently [10].

The concentration of DMBA and TMBZ, the substrate and reaction product of the *Pf*BAL activity assay, were determined by high performance liquid chromatography (HPLC). The samples were prepared for HPLC analysis by centrifugation at 15,800×*g* for 1 min. Subsequently, the supernatant was transferred to HPLC vials equipped with inlets. For analysis, 10 µl of samples were injected into a Thermo Scientific Dionex Ultimate 3000 HPLC system containing a diode-array detector DAD-3000 (ThermoFisher Scientific, Waltham, MA, USA). As stationary phase, a Chiralpak^®^ IE column was used (4,6 µm × 250 mm, 5 µm particle size column, Daicel, Tokyo, Japan), which was combined with a pre-column of the same material (Chiralpak^®^ IE 4 mm x 10 mm; Daicel, Tokyo, Japan). The columns were tempered to 20 °C. Separation was achieved under isocratic elution (flow rate 1 ml min^−1^) using a binary mobile phase consisting of 50% (v/v) dd H_2_O and 50% (v/v) acetonitrile. The analytes eluted at retention times of 6.1 min for *p*-MBA (270 nm), 7.6 min for DMBA (215 nm); and 9.4 min for (*R*)-TMBZ (215 nm). To quantify substrate and product, a calibration of DMBA and TMBZ was performed [32].

### Scanning electron microscopy

Scanning electron microscopy images of CatIBs were taken by Steffen Köhler from the Center for Advanced Imaging (CAi) at the Heinrich-Heine University Düsseldorf with a Leo 1430 VP scanning electron microscopy (Carl Zeiss AG, Oberkochen, Germany). For sample preparation, 2 mg ml^−1^ lyophilized TDoT-CatIBs or 4 mg ml^−1^ 3HAMP-CatIBs were used. CatIB solutions (2.5 µl) were fixed on a silicon disk (VWR, Radnor, Pennsylvania, USA) with 2.5% (v/v) glutaraldehyde in 100 mM sodium phosphate buffer, pH 7.2 for 2 h at 25 °C and 250 rpm and rinsed three times for 10 min with buffer [91]. Afterwards, the samples were dehydrated through a graded ethanol series (30, 50, 70, 95, and 100%) for 15 min, respectively. Samples were dried by critical point method and coated with gold at the CAi before images were taken at an accelerating voltage of 15 or 19 kV.

### Computational analysis of sequence-based and structural determinants of CatIB formation

The aggregation propensity of the target proteins, as well as of the corresponding TDoT fusions, was inferred from their amino acid sequence by using the AGGRESCAN tool (http://bioinf.uab.es/aggrescan) [72] (see Additional file 1: Table S1). Implementation tests and details about the algorithm employed by AGGRESCAN have been provided elsewhere [11, 72]. The program provides several parameters that serve as a global indicator for the aggregation propensity of a given amino acid sequence. The average aggregation-propensity values per amino acid (a^4^v) normalized to a 100-residue protein (Na^4^vSS) were employed as quantitative descriptors for aggregation propensity. Those values have previously been shown to be good indicators for changes in aggregation properties, due to the introduction of point mutations, and have also been employed for the differentiation of soluble, unfolded, amyloid- and IB-forming proteins [72].

Alternatively, the presence/absence of large hydrophobic surface patches was considered as structural proxy for the aggregation propensity of a given target protein. Therefore, for each target protein, the pdb coordinates representing the most likely native oligomer were obtained from the pdb data bank (https://www.rcsb.org) [92]. Alternative oligomeric assemblies were derived using the PISA webserver [‘Protein interfaces, surfaces and assemblies’ service PISA at the European Bioinformatics Institute (http://www.ebi.ac.uk/pdbe/prot_int/pistart.html)] [93]. All solvent molecules and heteroatoms were removed before surface calculations were performed. The surface properties of the target proteins were evaluated by using the Rosetta protein design software [75, 76] by employing the hpatch tool [94]. The hpatch tool identifies surface localized clusters of hydrophobic atoms (hydrophobic patches) and provides a Pymol selection term for visualization of each identified patch. The overall patch area was calculated by summation over all identified patches. Patch areas and the overall solvent accessible surface area (SASA) were calculated with Pymol 1.7.0.0 (Schrödinger, LCC, New York, NY, USA). PDB-IDs and additional information about the employed structures and assemblies is provided in Additional file 1: Table S2.

## Additional file

**Additional file 1.** Additional information containing Additional Results, Methods, DNA and amino acid sequences of the fusion proteins and Additional references.

## Abbreviations

IBs: inclusion bodies
CatIBs: catalytically-active inclusion bodies
FIBs: functional inclusion bodies
*E. coli*: *Escherichia coli*
CLEAs: cross-linked enzyme aggregates
FPs: fluorescent proteins
TDoT: coiled-coil tetramerization domain of tetrabrachion
HAMP: histidine kinases, adenylyl cyclases, methyl-accepting chemotaxis proteins, and phosphatases
3HAMP: coiled-coil HAMP domain of the Aer2 oxygen sensor
SEM: scanning electron microscopy
SASA: solvent accessible surface area
