# Supplementary material for: Tailoring the properties of (catalytically)-active inclusion bodies
Source: Microb Cell Fact. 2019 Feb 7;18:33. doi: 10.1186/s12934-019-1081-5 (PMC6367779; doi:10.1186/s12934-019-1081-5)
Supplement: Supplementary file 1 — Additional file 1. Additional information containing Additional Results, Methods, DNA and amino acid sequences of the fusion proteins and Additional references. [file 12934_2019_1081_MOESM1_ESM.pdf]

Additional file 1

Tailoring the properties of (catalytically)-active inclusion bodies

V. D. Jäger[1,5+], R. Kloss[2,5+], A. Grünberger[2,4], S. Seide[2], D. Hahn[2], T. Karmainski[2], M. Piqueray[1], J. Embruch[2], S. Longerich[1], U. Mackfeld[2], K.-E. Jaeger[1,2,5], W. Wiechert[2,5], M. Pohl[2,5], and U. Krauss[1,5*]

[1]Institut für Molekulare Enzymtechnologie, Heinrich-Heine-Universität Düsseldorf, Forschungszentrum Jülich, 52425 Jülich, Germany

[2] Forschungszentrum Jülich GmbH, IBG-1: Biotechnology, 52425 Jülich, Germany

[4] Multiscale Bioengineering, Bielefeld University, Universitätsstraße 25, 33615 Bielefeld, Germany

[5] Bioeconomy Science Center (BioSC), c/o, Forschungszentrum Jülich, 52425 Jülich, Germany

*Corresponding author

+Both authors contributed equally

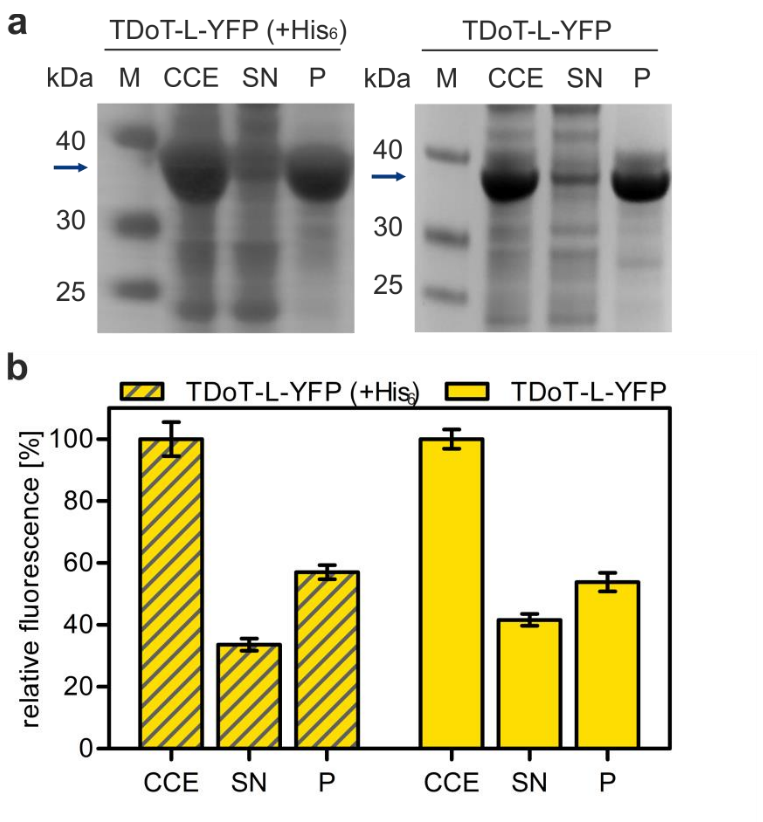

Figure S1: Improving the CatIB strategy by deleting the Hexahistidine-tag (His_6-tag) from the original vector [1]. Evaluation by (a) SDS-PAGE analysis and (b) fluorescence distribution for TDoT-L-YFP (+His) (36.8 kDa) and TDoT-L-YFP (34.6). After cell disruption, the crude cell extract (CCE) was separated by centrifugation into the soluble protein containing supernatant (SN) and the insoluble IB containing pellet (P) fraction. (a) SDS-PAGE analysis of the respective protein fractions: CCE, SN, and P. The molecular mass of the respective fusion protein is indicated by arrows. The protein content in the SN was measured according to Bradford [2]. (b) Normalized fluorescence in the CCE, SN, and P fractions of the respective proteins. Note, that the P fraction was washed once with water and centrifuged again before the fluorescence measurement. The fluorescence in CCE, SN, and P fractions was calculated relative to the activity in the CCE, which was set to 100%. Error bars correspond to the standard deviation of the mean derived from at least three technical replicates.

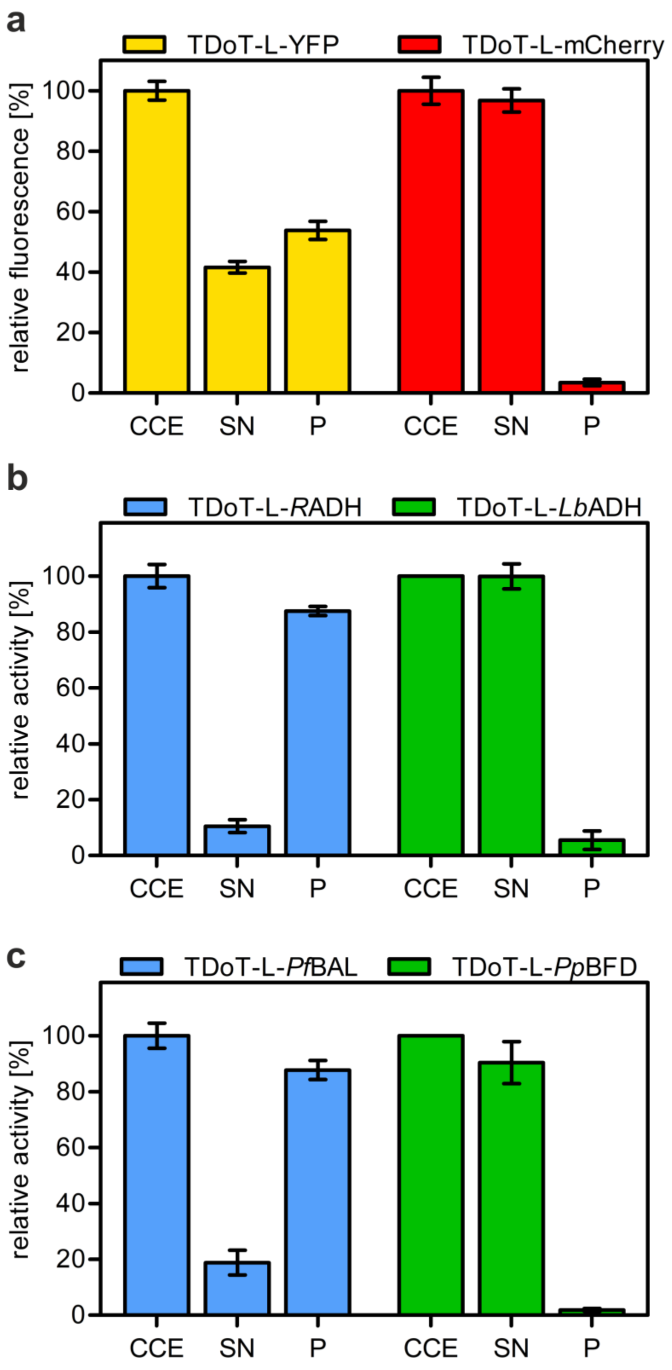

Figure S2: The fluorescence/activity distribution of (a) TDoT-L-YFP-, TDoT-L-mCherry-, (b) TDoT-L-*R*ADH-, TDoT-L-*Lb*ADH-, (c) TDoT-L-*Pf*BAL- and TDoT-L-*Pp*BFD-CatIB. Sample preparation was done as in Figure S1. Initial rate activities were measured by carboligation of 3,5-dimethoxybenzaldehyde (DMBA) to the respective benzoin catalyzed by TDoT-L-*Pf*BAL-CatIBs and decarboxylation of benzoylformate to benzaldehyde by TDoT-L-*Pp*BFD-CatIBs, which was followed by the reduction to benzyl alcohol by horse liver HL-ADH under the oxidation of NADH. Initial rate activities of the ADH enzymes were measured by reduction of 1-phenylethanol (TDoT-L-*Lb*ADH) or cyclohexaone (TDoT-L-*R*ADH) under the consumption of NADPH. For reaction equations see Figure S13. Error bars correspond to the standard deviation of the mean derived from at least three biological replicates. Controls expressing the corresponding soluble versions of YFP, mCherry, *R*ADH, and *Pf*BAL (with TDoT tag) have been presented before [3].

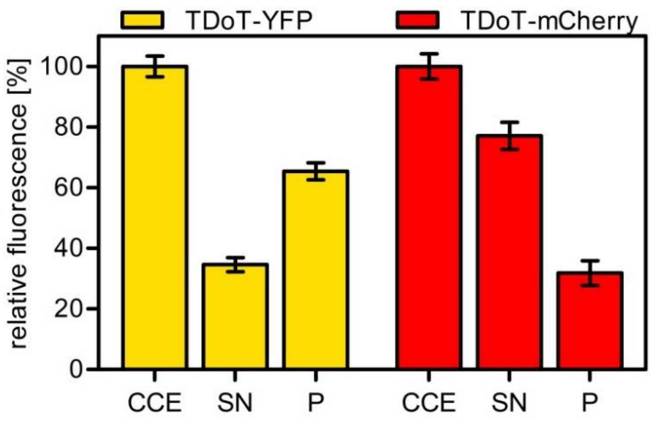

Figure S3: The fluorescence distribution of the linker-free variants TDoT-YFP- and TDoT-mCherry-FIBs. Sample preparation was done as in Figure S1. Error bars correspond to the standard deviation of the mean derived from at least three biological replicates.

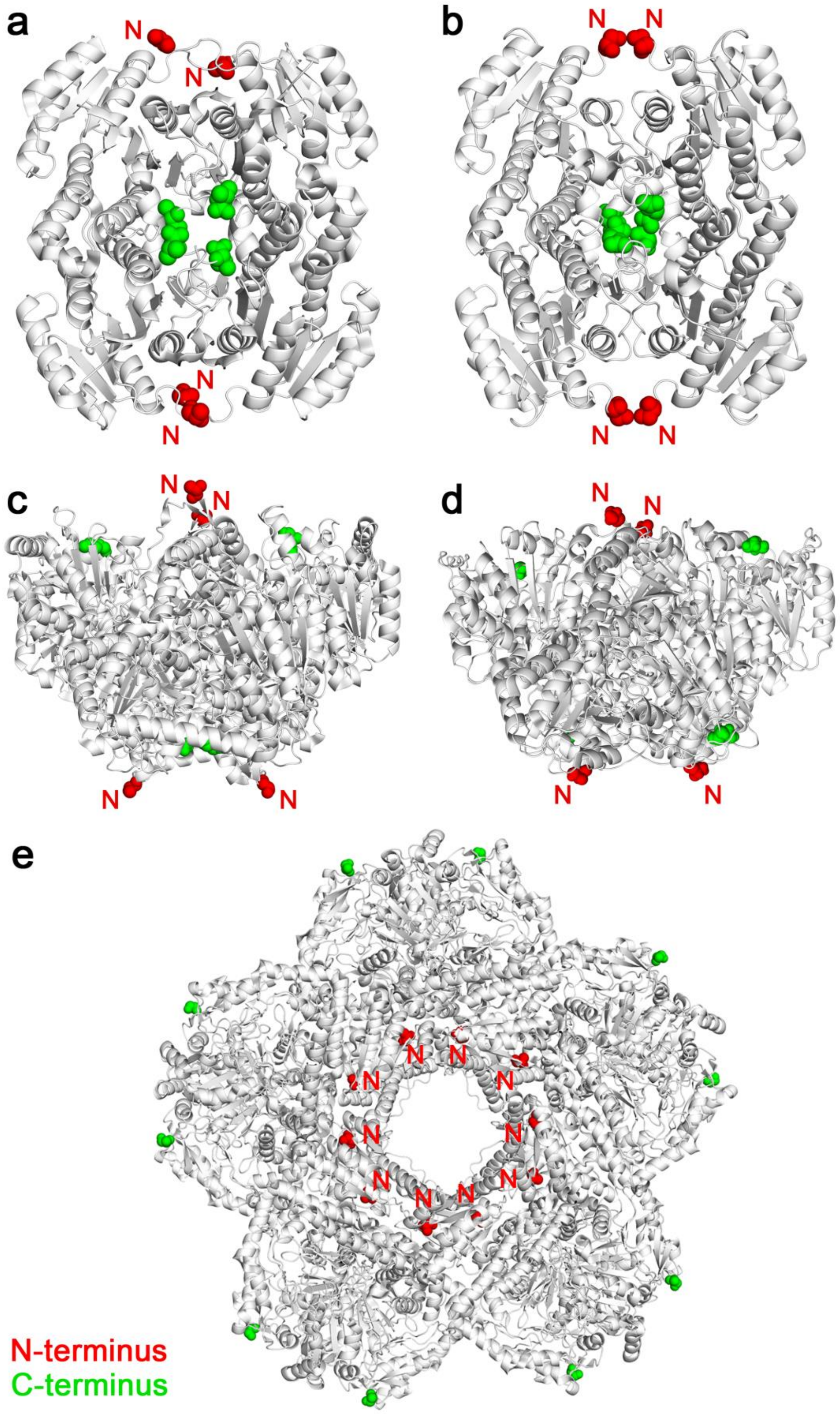

Figure S4: Structures of the multimeric target enzymes of this study: (a) *R*ADH (PDB: 4BMN), (b) *Lb*ADH (PDB: 1ZK4), (c) *Pf*BAL (PDB: 2UZ1), (d) *Pp*BFD (PDB: 5DEI) and (e) *Ec*LDC (PDB: 5FKZ). All subunits are shown in grey cartoon representation, while the N- and C-terminal amino acids are shown as spheres in red (N-terminus) and green (C-terminus). For clarity, only the N-terminus is labelled in each panel. The N-terminus is accessible in *R*ADH, *Lb*ADH, *Pf*BAL, and *Pp*BFD. In contrast, it is buried within the decameric structure in the N-terminal wing domain of the *Ec*LDC multimer, whereas the C-terminus is located at the outer part in the C-terminal domain [4] and hence should be accessible for tag fusion.

a

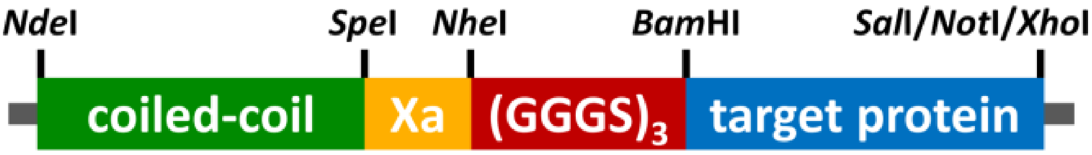

b

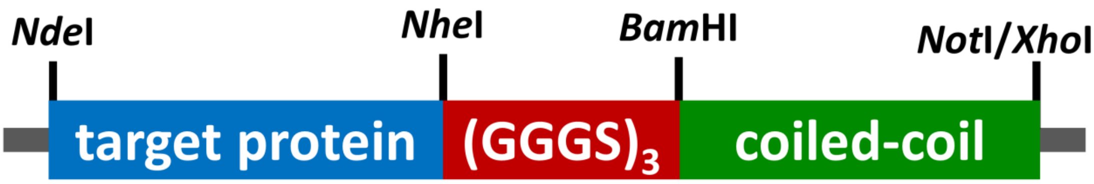

Figure S5: Schematic illustration of the N-terminal (a) and C-terminal (b) fusion strategy employed for the generation of CatIBs/FIBs. All expression vectors/gene fusions are constructed modularly, so that every element can be easily exchanged or deleted, by the use of the depicted restriction sites. Gene fusions consist of coding sequences for: the respective target protein (blue), a coiled-coil domain (green), for which we used the TDoT coiled-coil domain (52 amino acids) of the cell-surface protein tetrabrachion of *Staphylothermus marinus* [5] or the 3HAMP coiled coil (172 amino acids) of the soluble oxygen sensor Aer2 of *Pseudomonas aeruginosa* [6]. Whenever mentioned gene fusions additionally contained a linker region, comprising a cleavage site of the Factor Xa protease (orange, 4 amino acids) and a $(GGGS)_3$-motif (red, 12 amino acids). The gene fusion design shown in (b) was chosen only for the enzyme *Ec*LDC [4].

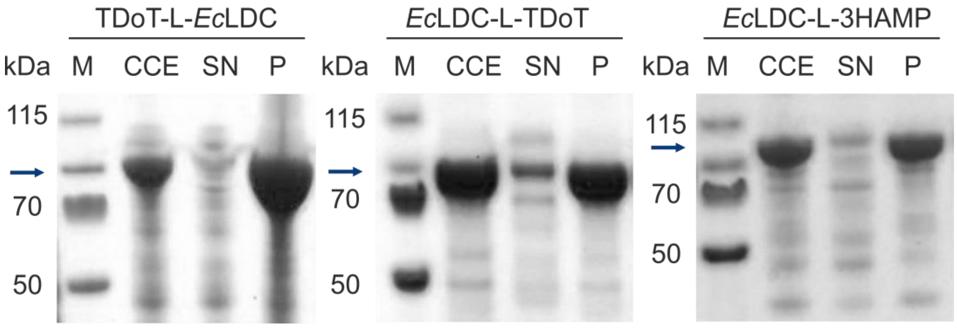

Figure S6: SDS-PAGE analysis of the respective protein fractions of TDoT-L-*Ec*LDC-CatIBs (90.3 kDa), *Ec*LDC-L-TDoT-CatIBs (87.5 kDa) and *Ec*LDC-L-3HAMP-CatIBs (100.5 kDa): crude cell extract (CCE), supernatant (SN), and pellet (P). The target protein is indicated by arrows. Sample preparation was done as in Figure S1.

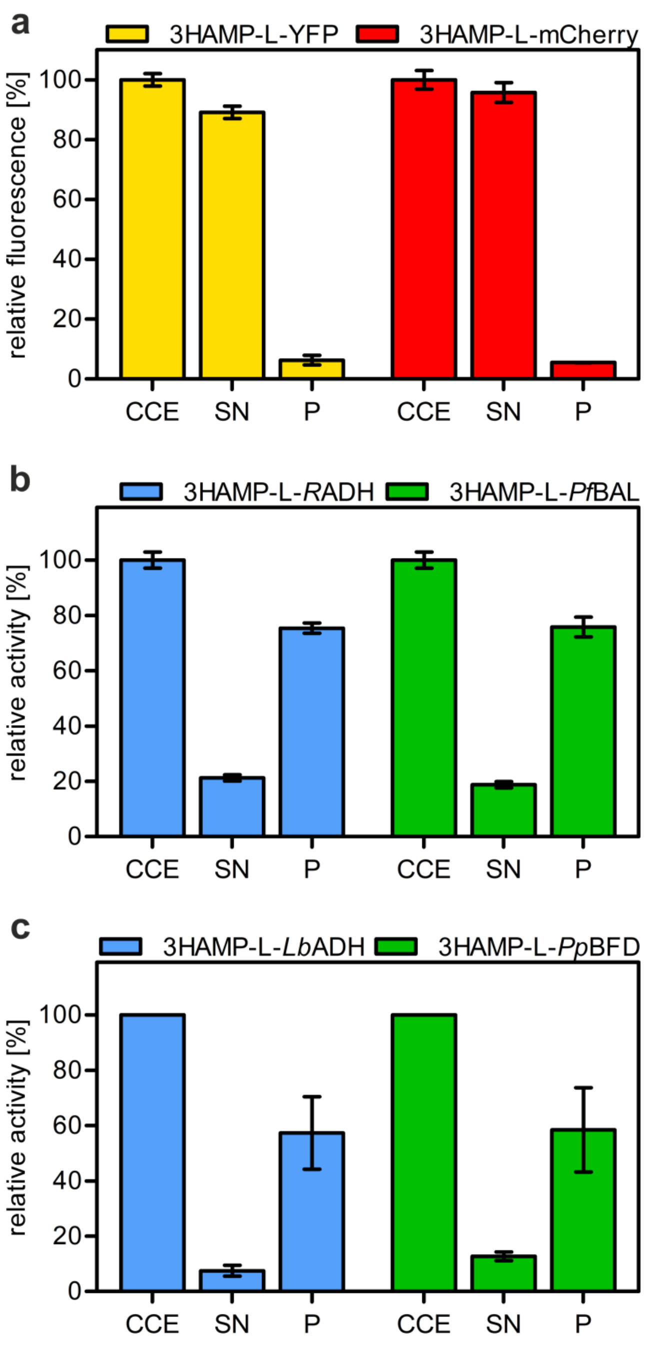

Figure S7: The fluorescence/activity distribution of (a) 3HAMP-L-YFP-, 3HAMP-L-mCherry-, (b) 3HAMP-L-*R*ADH-, 3HAMP-L-*Pf*BAL-, (c) 3HAMP-L-*Lb*ADH-, and 3HAMP-L-*Pp*BFD-CatIBs.. Sample preparation was done as in Figure S1. Initial rate activities were measured by carboligation of 3,5-dimethoxybenzaldehyde (DMBA) to the respective benzoin catalyzed by 3HAMP-L-*Pf*BAL-CatIBs and decarboxylation of benzoylformate to benzaldehyde by 3HAMP-L-*Pp*BFD-CatIBs, which was followed by the reduction to benzyl alcohol by horse liver HL-ADH under the oxidation of NADH. The initial rate activities of the ADH enzymes were measured by reduction of 1-phenylethanol (3HAMP-L-*Lb*ADH) or cycloheaxone (3HAMP-L-*R*ADH) under the consumption of NADPH. For reaction equations see Figure S13. Error bars correspond to the standard deviation of the mean derived from at least three biological replicates.

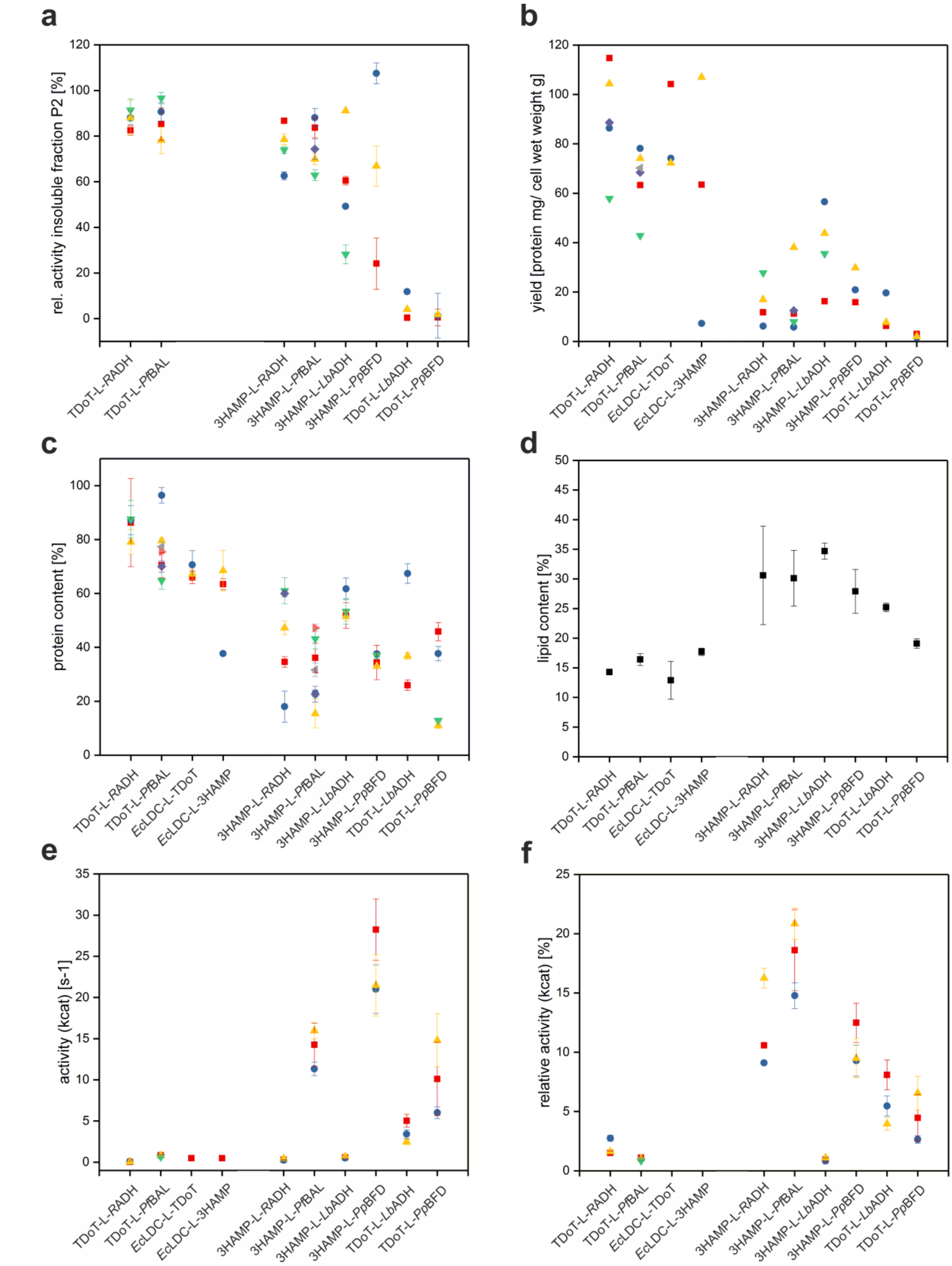

Figure S8: Mean values for each biological replicate with standard deviation (A) for the activity in the pellet compared to CCE, (B) yield (mg protein for 1 g wet cell weight), (C) protein content, and (D) lipid content, based on the dry weight of lyophilized CatIBs, (E) initial rate activity (k_{cat}) and (F) residual activity compared to the soluble enzyme.

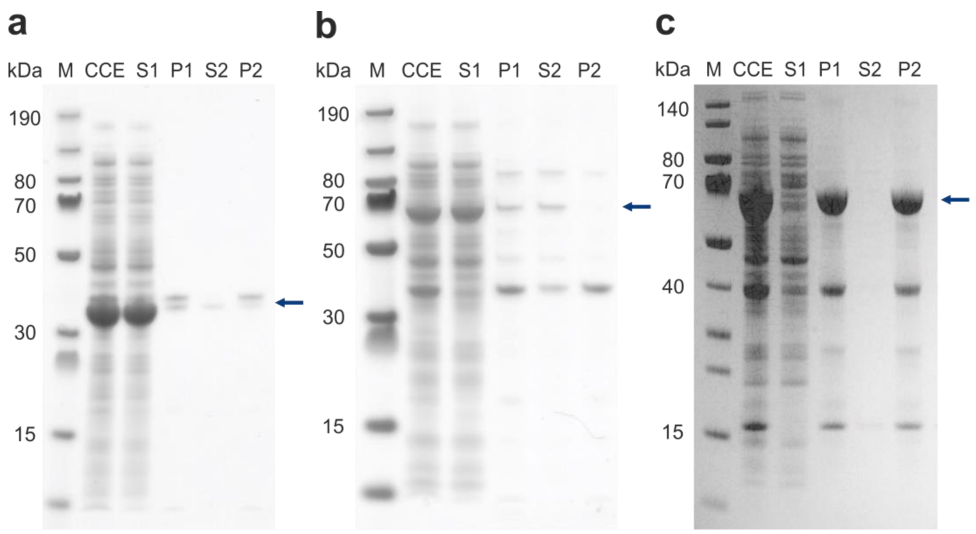

Figure S9: Evaluation of the CatIB strategy by SDS-PAGE analysis of the respective protein fractions of (a) TDoT-L-*Lb*ADH (34.3 kDa), (b) TDoT-L-*Pp*BFD (64.2 kDa) and (c) TDoT-L-*Pf*BAL CatIBs (66.5 kDa): crude cell extract (CCE), supernatant (SN), and pellet (P). The target protein is indicated by arrows. Sample preparation was done as in Figure S1.

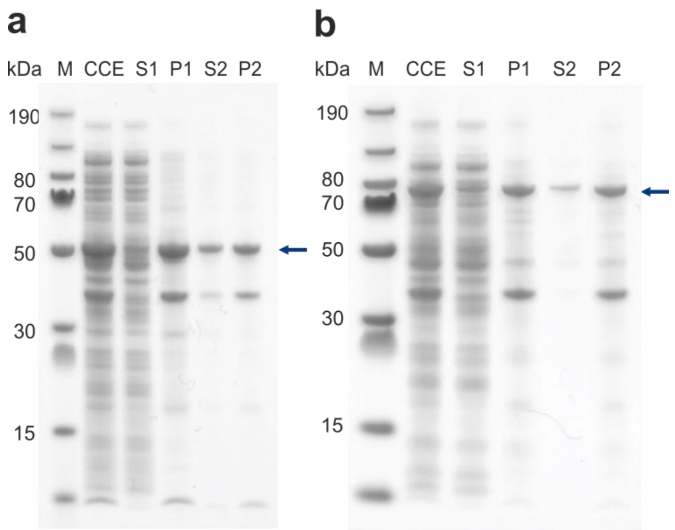

Figure S10: Evaluation of the CatIB strategy by SDS-PAGE analysis of the respective protein fractions of (a) 3HAMP-L-*Lb*ADH- (47.1 kDa), (b) 3HAMP-L-*Pp*BFD-CatIBs (77.0 kDa): crude cell extract (CCE), supernatant (SN), and pellet (P). The target protein is indicated by arrows. Sample preparation was done as in Figure S1.

Table S1: Sequence-based aggregation-propensity prediction for each target protein and the corresponding TDoT fusion. All predictions were carried out using the AGGRESCAN web server (http://bioinf.uab.es/aggrescan) [7]. Displayed are the average aggregation-propensity values per amino acid (a^4v) normalized to a 100 residue protein (Na^4vSS) and the relative change in Na^4vSS (ΔNa^4vSS) due to fusion of the TDoT domain.

**target protein**	**Na^4vSS**	**TDoT-target-protein fusion**	**Na^4vSS**	**ΔNa^4vSS***
YFP	-7.9	TDoT-L-YFP	-6.5	17.7
mCherry	-16.6	TDoT-L-mCherry	-12.6	24.1
*Bs*LA	0.8	TDoT-L-*Bs*LA	-1.4	-275.0
*At*HNL	3.9	TDoT-L-*At*HNL	1.9	-51.3
*Ec*MenD	-3.2	TDoT-L-*Ec*MenD	-3.1	3.1
*R*ADH	-1.7	TDoT-L-*R*ADH	-1.5	11.8
*Lb*ADH	-4.5	TDoT-L-*Lb*ADH	-3.8	15.6
*Pf*BAL	3.6	TDoT-L-*Pf*BAL	2.9	-19.4
*Pp*BFD	-1.3	TDoT-L-*Pp*BFD	-1.4	-7.7

*calculated according to: $\Delta Na^4vSS = \left(\frac{(Na^4vSS_{fusion} - Na^4vSS_{target})}{|Na^4vSS_{target}|} \times 100\right)$ [7].

Table S2: Surface properties of the target proteins used in this study. The presence/absence of large hydrophobic surface patches for the corresponding target protein structures was quantified using the hpatch tool implemented in Rosetta [8-10]. Surface areas were quantified using Pymol 1.7.0.0 (Schrödinger, LCC, New York, NY, USA).

target protein	PDB-ID	comments	hydrophobic patch area [A^2]	solvent accessible surface area (SASA) [A^2]	hydrophob. patch area / overall SASA [%]
YFP	1YFP	asu, monomer, chain A and B	1000	9467	10.6
YFP*	1YFP	1st PISA dimer	1085	16914	6.4
mCherry*	2H5Q	asu, monomer	0	9493	0
*Bs*LA*	1ISP	asu, monomer	972	7867	12.4
*At*HNL*	3DQZ	asu, dimer, chain A and B	1692	18662	9.1
*Ec*MenD	2JLC	asu, dimer	1566	34022	4.6
*Ec*MenD*	2JLC	1st PISA tetramer	3797	54278	7.0
*R*ADH	4BMN	tetramer	1183	27318	4.3
*R*ADH*	4BMN	1st PISA dimer	2068	17423	11.9
*Lb*ADH	1ZK4	1st PISA tetramer	2748	27590	9.9
*Lb*ADH*	1ZK4	2nd PSIA dimer	874	18316	4.8
*Pf*BAL*	2UZ1	asu, tetramer	5194	52235	9.9
*Pp*BFD*	5DEI	asu, tetramer	185	69766	0.27

* final data used in Figure 6c; main paper; asu: the pdb coordinates present in the asymmetric unit were used for surface calculations.

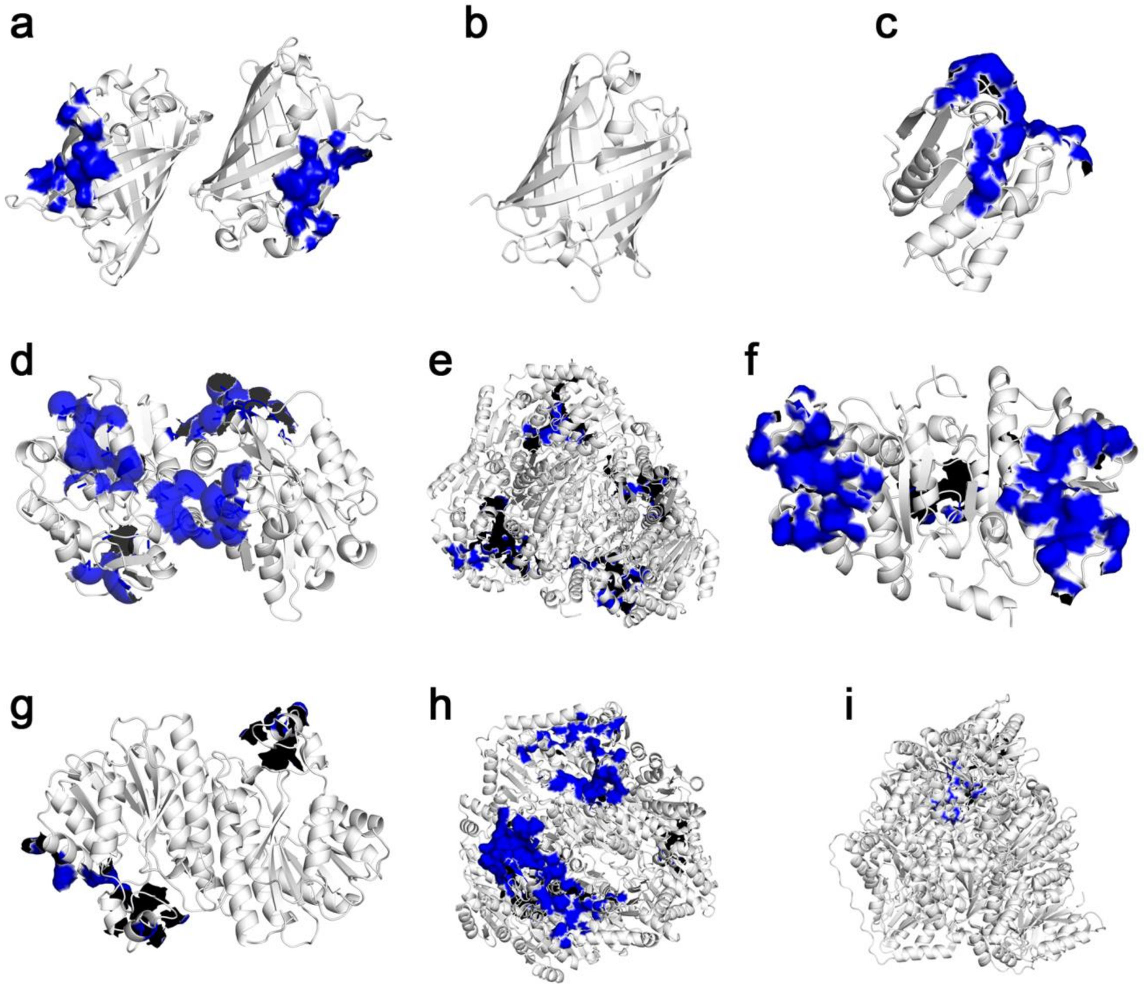

Figure S11: Hydrophobic patch analysis of (a) YFP, (b) mCherry, (c) *Bs*LA, (d) *At*HNL, (e) *Ec*MenD, (f) *R*ADH, (g) *Lb*ADH, (h) *Pf*BAL, and (i) *Pp*BFD. Structures are shown in cartoon representation in grey with the Rossetta-identified hydrophobic surface patches shown as blue surfaces [9, 10]. PDB-IDs were as follows YFP (1YFP), mCherry (2H5Q), *Bs*LA (1ISP), *At*HNL (3DWZ), *Ec*MenD (2JLC), *R*ADH (4BMN), *Lb*ADH (1ZK4), *Pf*BAL (2UZ1) and *Pp*BFD (5DEI).

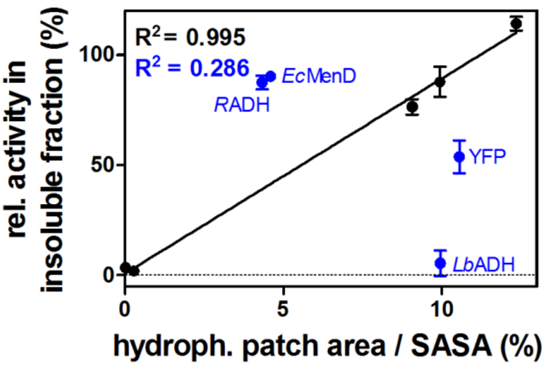

Figure S12: Computational analysis of the structural determinants of CatIB formation. The presence/absence of large hydrophobic surface patches for the corresponding target protein structures was quantified using the hpatch tool implemented in Rosetta [8-10]. Surface areas were quantified using Pymol 1.7.0.0 (Schrödinger, LCC, New York, NY, USA). CatIB-formation was plotted as the relative activity in the insoluble fraction (Table S6). Coefficient of determination (R^2) values are always given excluding the blue-highlighted outliers (black) and including the outliers (blue).

Table S3: Plasmids used in this study. DNA and amino acid sequences of all gene fusions are given in the annex.

Vector	Genotyp	Description
pET28a	*ColE1 lacZ'* Kan^R P_{T7} P_{lac}	Merck (Darmstadt, Germany)
pTDoT-Linker-YFP	pET-28a, P_{T7}, gene fusion [*His-tag-tdot*-factor Xa recognition site-*(GGGS)*$_3$ *linker-yfp*]	[11]
pTDoT-L-YFP	pET-28a, P_{T7}, gene fusion [*tdot*-factor Xa recognition site-*(GGGS)*$_3$ *linker-yfp*]	pTDoT-Linker-YFP derivative, insertion of a 40 bp *Xba*I/*Nde*I RBS containing fragment of pET28a in pTDoT-Linker-YFP; without the 99 bp fragment containing RBS and *His-tag* [3]
pTDoT-YFP	pET-28a, P_{T7}, gene fusion [*tdot-yfp*]	pTDoT-L-YFP derivative, insertion of a 155 bp *Nde*I/*Bam*HI *tdot*-containing fragment in pTDoT-L-YFP; without the 215 bp fragment containing *tdot*, factor Xa recognition site and *(GGGS)*$_3$ *linker*
p3HAMP-L-YFP	pET-28a, P_{T7}, gene fusion [*3hamp*-factor Xa recognition site-*(GGGS)*$_3$ *linker-yfp*]	pTDoT-L-YFP derivative, insertion of a 518 bp *Nde*I/*Spe*I *3hamp*-containing fragment in pTDoT-L-YFP; without the 155 bp fragment containing *tdot*,
pTDoT-L-mCherry	pET-28a, P_{T7}, gene fusion [*tdot*-factor Xa recognition site-*(GGGS)*$_3$ *linker-mcherry*]	pTDoT-L-YFP derivative, insertion of 717 bp PCR-amplified *Bam*HI/*Sal*I *mcherry* fragment in pTDoT-L-YFP; without the 726 bp fragment containing *yfp* [3]
pTDoT-mCherry	pET-28a, P_{T7}, gene fusion [*tdot-mcherry*]	pTDoT-L-mCherry derivative, insertion of 155 bp *Nde*I/*Bam*HI *tdot*-containing fragment in pTDoT-L-mCherry; without the 215 bp fragment containing *tdot*, factor Xa recognition site and *(GGGS)*$_3$ *linker* [3]
p3HAMP-L-mCherry	pET-28a, P_{T7}, gene fusion [*3hamp*-factor Xa recognition site-*(GGGS)*$_3$ *linker-mcherry*]	pTDoT-L-mCherry derivative, insertion of a 518 bp *Nde*I/*Spe*I *3hamp*-containing fragment in pTDoT-L-mCherry; without the 155 bp fragment containing *tdot*,
pET22b-*R*ADH	pET22b, P_{T7}, *radh* gene	[12]
pTDoT-L-*R*ADH	pET-28a, $P_{T7,}$ gene fusion [*tdot*- factor Xa recognition site-*(GGGS)*$_3$ *linker-radh*]	pTDoT-L-YFP derivative, insertion of 756 bp PCR-amplified *Bam*HI/*Sal*I *radh* fragment in pTDoT-L-YFP; without the 726 bp fragment containing *yfp* [3]
p3HAMP-L-*R*ADH	pET-28a, P_{T7}, gene fusion [*3hamp*-factor Xa recognition site-*(GGGS)*$_3$ *linker-radh*]	pTDoT-L-*R*ADH derivative, insertion of a 518 bp *Nde*I/*Spe*I *3hamp*-containing fragment in pTDoT-L-*R*ADH; without the 155 bp fragment containing *tdot*
pET21a-*Lb*ADH	pET21a, P_{T7}, *lbadh* gene	[13]
pTDoT-L-*Lb*ADH	pET-28a, $P_{T7,}$ gene fusion [*tdot*-factor Xa recognition site-*(GGGS)*$_3$ *linker-lbadh*]	pTDoT-L-YFP derivative, insertion of 766 bp PCR-amplified *Bam*HI/*Sal*I *lbadh* fragment in pTDoT-L-YFP; without the 726 bp fragment containing *yfp*
p3HAMP-L-*Lb*ADH	pET-28a, P_{T7}, gene fusion [*3hamp*-factor Xa recognition site-*(GGGS)*$_3$ *linker-lbadh*]	p3HAMP-L-*R*ADH derivative, insertion of 766 bp PCR-amplified *Bam*HI/*Sal*I *lbadh* fragment in p3HAMP-L-*R*ADH; without the 756 bp fragment containing *radh*
pKK233_2-*Pf*BAL-*His*	pKK233_2 P_{trc}, gene fusion [*pfbal, His-tag]*	[14]
pTDoT-L-*Pf*BAL	pET-28a, P_{T7}, gene fusion [*tdot*-factor Xa recognition site-*(GGGS)*$_3$ *linker-pfbal*]	pTDoT-L-YFP derivative, insertion of 1699 bp PCR-amplified *Bam*HI/*Not*I *pfbal* fragment in pTDoT-L-YFP; without the 739 bp fragment containing *yfp* [3, 15]
p3HAMP-L-*Pf*BAL	pET-28a, P_{T7}, gene fusion [*3hamp*-factor Xa recognition site-*(GGGS)*$_3$ *linker-pfbal*]	pTDoT-L-*Pf*BAL derivative, insertion of a 518 bp *Nde*I/*Spe*I *3hamp*-containing fragment in pTDoT-L-*Pf*BAL; without the 155 bp fragment containing *tdot* [15]
pKK233_2-*Pp*BFD *L476Q-His*	pKK233_2 P_{trc}, gene fusion [*ppbfd, His-tag]*	[16]
pTDoT-L-*Pp*BFD *L476Q*	pET-28a, $P_{T7,}$ gene fusion [*tdot*-factor Xa recognition site-*(GGGS)*$_3$ *linker-ppbfd*]	pTDoT-L-YFP derivative, insertion of 1600 bp PCR-amplified *Bam*HI/*Not*I *ppbfd* fragment in pTDoT-L-YFP; without the 739 bp fragment containing *yfp*

p3HAMP-L-*Pp*BFD *L476Q*	pET-28a, P_{T7}, gene fusion [*3hamp*-factor Xa recognition site-*(GGGS)*$_3$ *linker-ppbfd*]	p3HAMP-L-*Pf*BAL derivative, insertion of 766 bp PCR-amplified *Bam*HI/*Not*I *ppbfd* fragment in p3HAMP-L-*Pf*BAL; without the 1699 bp fragment containing *pfbal*
pTDoT-L-*Ec*LDC	pET-28a, $P_{T7,}$ gene fusion [*tdot*-factor Xa recognition site-*(GGGS)*$_3$ *linker-ecldc*]	pTDoT-L-YFP derivative, insertion of 2148 bp PCR-amplified *Bam*HI/*Sal*I *ecldc* fragment in pTDoT-L-YFP; without the 726 bp fragment containing *yfp*
p*Ec*LDC-L-TDoT	pET-28a, $P_{T7,}$ gene fusion [*ecldc-(GGGS)*$_3$ *linker-tdot*]	pTDoT-L-*Pf*BAL derivative, i) insertion of 2144 bp PCR-amplified *Nde*I/*Nhe*I *ecld* fragment in pTDoT-L-*Pf*BAL; without the 173 bp fragment containing *tdot-Xa*, ii) insertion of 160 bp PCR-amplified *Bam*HI/*Not*I *tdot* fragment in p*Ec*LDC-L-*Pf*BAL, without the 1699 bp fragment containing *bfbal* [17]
p*Ec*LDC-L-3HAMP	pET-28a, $P_{T7,}$ gene fusion [*ecldc-(GGGS)*$_3$ *linker-3hamp*]	p*Ec*LDC-L-TDoT derivative, insertion of 526 bp PCR-amplified *Bam*HI/*Not*I *3hamp* fragment in p*Ec*LDC-L-TDoT; without the 160 bp fragment containing *tdot*

Table S4: Oligonucleotide primers used in this study. Restriction endonuclease cleavage sites are underlined.

Name	Sequence (5' - 3')	Application
*Bam*HI_YFP_fw	ATATATGGATCCATGGTGAGCAAGGGCGAG	PCR amplification of *yfp*
YFP_*Sal*I_rv	ATATATGTCGACTTACTTGTACAGCTCGTCCATG	
*Bam*HI_mCherry_fw	ATATATGGATCCATGGTGAGCAAGGGCGAGGAGG	PCR amplification of *mcherry*
mCherry_*Sal*I_rv	ATATATGTCGACTTACTTGTACAGCTCGTCCATGCCGC	
*Bam*HI_*Pf*BAL_fw	ATATATGGATCCATGGCGATGATTACAGGCGGCGAAC	PCR amplification of *pfbal*
*Pf*BAL_*Not*I_rv	ATATATGCGGCCGCTTATGCGAAGGGGTCCATG	
*Bam*HI_*R*ADH_fw	ATATATGGATCCATGTATCGTCTGCTGAATAAAAACCGC	PCR amplification of *radh*
*R*ADH_*Sal*I_rv	ATATATGTCGACTTAAACCTGGGTCAGACCACCATC	
*Nde*I_ TDoT_fw	ATATATCATATGATCATTAACGAAACTGCCGATGAC	Removal of factor Xa recognition site and $(GGGS)_3$ linker
TDoT_*Bam*HI_rv	TATATAGGATCCAATGCTCGCGAGAATGGTG	
RBS_Oligo_fw	CTAGAAATAATTTTGTTTAACTTTAAGAAGGAGATATACA	Removal of His-Tag
RBS_Oligo_rv	TATGTATATCTCCTTCTTAAAGTTAAACAAAATTATTT	
*Bam*HI_*Lb*ADH_fw	ATATATGGATCCATGTCTAACCGTTTGGATGGTAAGGTAG	PCR amplification of *lbadh*
*Lb*ADH_*Sal*I_rev	ATATATGTCGACTCTATTGAGCAGTGTAGCCACCG	
*Bam*HI_BFD_fw	ATATATGGATCCATGGCTTCGGTACACGGCACCAC	PCR amplification of *ppbfd*
BFD_*Not*I_rev	ATATATGCGGCCGCTTAAGATCTCTTCACCGGGCTTAC	
*Bam*HI_LDC_fwd	ATATATGGATCCATGAACATCATCGCTATCATGGGCCC	PCR amplification of *ecldc*
LDC_*Sal*I_rev	ATATATGTCGACTTAGCCTGCCATCTTAAGGACGCG	
*Nde*I_LDC_fw	ATATATCATATGATGAACATCATCGCTATCATGGGCCC	PCR amplification of *ecldc*
LDC_*Nhe*I_rv	ATATATGCTAGCGCCTGCCATCTTAAGGACG	
*Bam*HI_TDot_fw	ATATATGGATCCATCATTAACGAAACTGCCGATGACATCG	PCR amplification of *tdot*
TDot_*Not*I_rv	ATATATGCGGCCGCTTAAATGCTCGCGAGAATG	
*Bam*HI_3HAMP_fw	ACGTATGGATCCATGGGCCTGTTTAACGCCCATGCAGTTG	PCR amplification of *3hamp*
3HAMP_*Not*I_rev	ACATATGCGGCCGCTTAATTGTAGGCGGCTGTGGCCAGC	

Table S5: The used strains are given with genotype and reference or source.

strains	genotype	reference or source
E. coli BL21 (DE3)	*F^- ompT hsdSB(rB$^-$ mB $^-$) gal dcm (λIts857ind1 Sam7 nin5 lacUV5-T7 gene1)*	[18]<br>Invitrogen (Carlsbad, USA)
E. coli DH5α	*supE44 ΔlacU169 (Φ80lacZDM15) hsdR17 recA1 endA1 gyrA96 thi-1 relA1*	Invitrogen (Carlsbad, USA)
E. coli SG 13009	*F^- ompT $hsdS_B$ (rB$^-$mB$^-$) dcm gal* (DE3)	Qiagen (Hilden Germany)

Table S6: Extinction coefficient and molecular weight of all used enzymes and proteins in soluble and CatIB form calculated with ExPASy ProtParam Tool (http://expasy.org/tools/protparam [19].

Protein	Extinction coefficient [L·mol^{-1}·cm^{-1}]	Molecular weight [kDa]
TDoT-L-YFP-CatIBs	26 485	34.6
TDoT-YFP-CatIBs	26 485	33.1
3HAMP-L-YFP-CatIBs	34 965	47.4
TDoT-L-mCherry-CatIBs	37 360	34.3
TDoT-mCherry-CatIBs	37 360	32.7
3HAMP-L-mCherry-CatIBs	45 840	47.1
soluble *R*ADH	14 440	26.7
TDoT-L-*R*ADH-CatIBs	17 420	34.3
3HAMP-L-*R*ADH-CatIBs	25 900	47.1
soluble *Lb*ADH	19 940	26.8
TDoT-L-*Lb*ADH-CatIBs	22 920	34.3
3HAMP-L-*Lb*ADH-CatIBs	31 400	47.1
soluble *Pf*BAL	52 160	60.0
TDoT-L-*Pf*BAL-CatIBs	55 140	66.5
3HAMP-L-*Pf*BAL-CatIBs	63 620	79.3
soluble *Pp*BFD	62 340	57.4
TDoT-L-*Pp*BFD-CatIBs	65 320	64.2
3HAMP-*L-Pp*BFD-CatIBs	73 800	77.0
TDoT-L-*Ec*LDC-CatIBs	109 210	90.3
*Ec*LDC-L-TDoT-CatIBs	109 210	87.5
*Ec*LDC-L-3HAMP-CatIBs	117 690	100.5

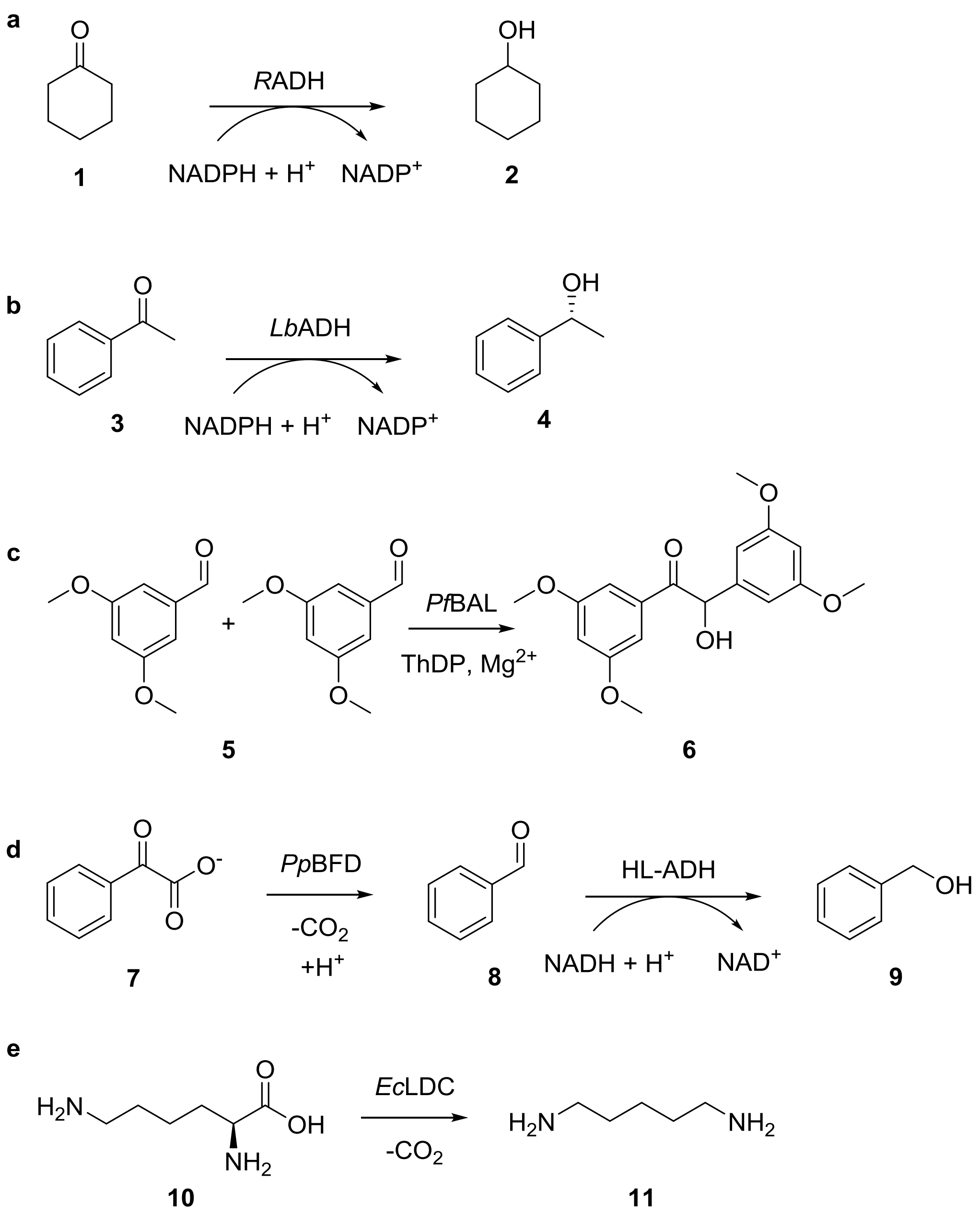

Figure S13: Reactions to measure the initial rate activities of the enzymes used in this study. (a) *R*ADH-CatIBs catalyze the reduction of cyclohexanone **1** to cyclohexanol **2** under the consumption of NADPH. (b) *Lb*ADH-CatIBs catalyze the reduction of acetophenone **3** to (*R*)-1-phenylethanol **4** under the consumption of NADPH. (c) *Pf*BAL-CatIBs catalyze the carboligation of 3,5-dimethoxybenzaldehyde (DMBA, **5**) to the respective benzoin (*R*)-(3,3´,5,5´)-tetramethoxybenzoin (TMBZ, **6**). (d) *Pp*BFD-CatIBs catalyze the decarboxylation of benzoylformate **7** to benzaldehyde **8** followed by the reduction to benzyl alcohol **9** by horse liver (HL-)ADH under the oxidation of NADH. (e) *Ec*LDC-CatIBs catalyze the decarboxylation of L-lysine **10** to cadaverine (1,5-diaminopentane, **11**).

DNA and amino acid sequence of the fusion constructs

The here employed variant of the yellow fluorescent protein (YFP) was derived from introducing the A206K mutation into enhanced YFP (eYFP). In contrast to mYFP [20] this variant lacks the Q69K mutation, which renders YFP less pH-sensitive in the neutral pH range [21]. The here used *Pp*BFD variant L476Q was derived from error-prone PCR and is described elsewhere [16].

TDoT-L-YFP

```
   M  I  I  N  E  T  A  D  D  I  V  Y  R  L  T  V  I  I  D  D  R  Y  E  S
CATATGATCATTAACGAAACTGCCGATGACATCGTTTATCGCCTGACAGTCATTATCGATGATCGCTACGAATCG
NdeI        TDoT
L  K  N  L  I  T  L  R  A  D  R  L  E  M  I  I  N  D  N  V  S  T  I  L  A
CTGAAAAACCTGATTACCTTACGTGCAGATCGCTTGGAGATGATCATCAATGACAATGTGTCCACCATTCTCGCG

S  I  T  S  I  E  G  R  A  S  G  G  G  S  G  G  G  S  G  G  G  S  G  S  M
AGCATTACTAGTATTGAAGGCCGTGCTAGCGGCGGTGGGTCTGGAGGCGGCTCAGGTGGTGGGTCGGGATCCATG
       SpeI     Xa          NheI       3xGGGS-Linker                 BamHI
V  S  K  G  E  E  L  F  T  G  V  V  P  I  L  V  E  L  D  G  D  V  N  G  H
GTGAGCAAGGGCGAGGAGCTGTTCACCGGGGTGGTGCCCATCCTGGTCGAGCTGGACGGCGACGTAAACGGCCAC
YFP
K  F  S  V  S  G  E  G  E  G  D  A  T  Y  G  K  L  T  L  K  F  I  C  T  T
AAGTTCAGCGTGTCCGGCGAGGGCGAGGGCGATGCCACCTACGGCAAGCTGACCCTGAAGTTCATCTGCACCACC

G  K  L  P  V  P  W  P  T  L  V  T  T  F  G  Y  G  L  Q  C  F  A  R  Y  P
GGCAAGCTGCCCGTGCCCTGGCCCACCCTCGTGACCACCTTCGGCTACGGCCTGCAGTGCTTCGCCCGCTACCCC

D  H  M  K  Q  H  D  F  F  K  S  A  M  P  E  G  Y  V  Q  E  R  T  I  F  F
GACCACATGAAGCAGCACGACTTCTTCAAGTCCGCCATGCCCGAAGGCTACGTCCAGGAGCGCACCATCTTCTTC

K  D  D  G  N  Y  K  T  R  A  E  V  K  F  E  G  D  T  L  V  N  R  I  E  L
AAGGACGACGGCAACTACAAGACCCGCGCCGAGGTGAAGTTCGAGGGCGACACCCTGGTGAACCGCATCGAGCTG

K  G  I  D  F  K  E  D  G  N  I  L  G  H  K  L  E  Y  N  Y  N  S  H  N  V
AAGGGCATCGACTTCAAGGAGGACGGCAACATCCTGGGGCACAAGCTGGAGTACAACTACAACAGCCACAACGTC

Y  I  M  A  D  K  Q  K  N  G  I  K  V  N  F  K  I  R  H  N  I  E  D  G  S
TATATCATGGCCGACAAGCAGAAGAACGGCATCAAGGTGAACTTCAAGATCCGCCACAACATCGAGGACGGCAGC

V  Q  L  A  D  H  Y  Q  Q  N  T  P  I  G  D  G  P  V  L  L  P  D  N  H  Y
GTGCAGCTCGCCGACCACTACCAGCAGAACACCCCCATCGGCGACGGCCCCGTGCTGCTGCCCGACAACCACTAC

L  S  Y  Q  S  K  L  S  K  D  P  N  E  K  R  D  H  M  V  L  L  E  F  V  T
CTGAGCTACCAGTCCAAACTGAGCAAAGACCCCAACGAGAAGCGCGATCACATGGTCCTGCTGGAGTTCGTGACC

A  A  G  I  T  L  G  M  D  E  L  Y  K  *
GCCGCCGGGATCACTCTCGGCATGGACGAGCTGTACAAGTAAGTCGACAAGCTTGCGGCCGCACTCGAG
                                             SalI              NotI      XhoI
```

TDoT-YFP

```
   M  I  I  N  E  T  A  D  D  I  V  Y  R  L  T  V  I  I  D  D  R  Y  E  S
CATATGATCATTAACGAAACTGCCGATGACATCGTTTATCGCCTGACAGTCATTATCGATGATCGCTACGAATCG
NdeI        TDoT
L  K  N  L  I  T  L  R  A  D  R  L  E  M  I  I  N  D  N  V  S  T  I  L  A
CTGAAAAACCTGATTACCTTACGTGCAGATCGCTTGGAGATGATCATCAATGACAATGTGTCCACCATTCTCGCG

S  I  G  S  M  V  S  K  G  E  E  L  F  T  G  V  V  P  I  L  V  E  L  D  G
AGCATTGGATCCATGGTGAGCAAGGGCGAGGAGCTGTTCACCGGGGTGGTGCCCATCCTGGTCGAGCTGGACGGC
```

```
          BamHI    YFP
D  V  N  G  H  K  F  S  V  S  G  E  G  E  G  D  A  T  Y  G  K  L  T  L  K
GACGTAAACGGCCACAAGTTCAGCGTGTCCGGCGAGGGCGAGGGCGATGCCACCTACGGCAAGCTGACCCTGAAG

F  I  C  T  T  G  K  L  P  V  P  W  P  T  L  V  T  T  F  G  Y  G  L  Q  C
TTCATCTGCACCACCGGCAAGCTGCCCGTGCCCTGGCCCACCCTCGTGACCACCTTCGGCTACGGCCTGCAGTGC

F  A  R  Y  P  D  H  M  K  Q  H  D  F  F  K  S  A  M  P  E  G  Y  V  Q  E
TTCGCCCGCTACCCCGACCACATGAAGCAGCACGACTTCTTCAAGTCCGCCATGCCCGAAGGCTACGTCCAGGAG

R  T  I  F  F  K  D  D  G  N  Y  K  T  R  A  E  V  K  F  E  G  D  T  L  V
CGCACCATCTTCTTCAAGGACGACGGCAACTACAAGACCCGCGCCGAGGTGAAGTTCGAGGGCGACACCCTGGTG

N  R  I  E  L  K  G  I  D  F  K  E  D  G  N  I  L  G  H  K  L  E  Y  N  Y
AACCGCATCGAGCTGAAGGGCATCGACTTCAAGGAGGACGGCAACATCCTGGGGCACAAGCTGGAGTACAACTAC

N  S  H  N  V  Y  I  M  A  D  K  Q  K  N  G  I  K  V  N  F  K  I  R  H  N
AACAGCCACAACGTCTATATCATGGCCGACAAGCAGAAGAACGGCATCAAGGTGAACTTCAAGATCCGCCACAAC

I  E  D  G  S  V  Q  L  A  D  H  Y  Q  Q  N  T  P  I  G  D  G  P  V  L  L
ATCGAGGACGGCAGCGTGCAGCTCGCCGACCACTACCAGCAGAACACCCCCATCGGCGACGGCCCCGTGCTGCTG

P  D  N  H  Y  L  S  Y  Q  S  K  L  S  K  D  P  N  E  K  R  D  H  M  V  L
CCCGACAACCACTACCTGAGCTACCAGTCCAAACTGAGCAAAGACCCCAACGAGAAGCGCGATCACATGGTCCTG

L  E  F  V  T  A  A  G  I  T  L  G  M  D  E  L  Y  K  *
CTGGAGTTCGTGACCGCCGCCGGGATCACTCTCGGCATGGACGAGCTGTACAAGTAAGTCGACAAGCTTGCGGCC
                                                            SalI          NotI
GCACTCGAGCACCACCACCACCACCACTGAGATCCGGCT
   XhoI
```

3HAMP-L-YFP

```
   M  G  L  F  N  A  H  A  V  A  Q  Q  R  A  D  R  I  A  T  L  L  Q  S  F
CATATGGGCCTGTTTAACGCCCATGCAGTTGCGCAGCAACGCGCGGATCGCATTGCGACTCTCCTGCAGTCCTTT
NdeI         3HAMP
A  D  G  Q  L  D  T  A  V  G  E  A  P  A  P  G  Y  E  R  L  Y  D  S  L  R

GCGGATGGTCAGTTGGACACCGCCGTGGGTGAAGCGCCAGCACCTGGTTACGAACGCCTGTATGACTCGCTTCGC
A  L  Q  R  Q  L  R  E  Q  R  A  E  L  Q  Q  V  E  S  L  E  A  G  L  A  E

GCCCTTCAGCGCCAACTGCGCGAACAACGTGCGGAGTTACAACAGGTTGAGAGCCTGGAAGCAGGCTTGGCTGAA
M  S  R  Q  H  E  A  G  W  I  D  Q  T  I  P  A  E  R  L  E  G  R  A  A  R

ATGAGTCGGCAGCATGAAGCAGGGTGGATTGACCAGACGATTCCGGCTGAACGGTTAGAGGGCCGTGCAGCACGT
I  A  K  G  V  N  E  L  V  A  A  H  I  A  V  K  M  K  V  V  S  V  V  T  A

ATCGCCAAAGGCGTGAATGAGCTGGTTGCTGCGCACATTGCGGTGAAAATGAAAGTCGTGAGCGTAGTCACCGCG
Y  G  Q  G  N  F  E  P  L  M  D  R  L  P  G  K  K  A  Q  I  T  E  A  I  D

TATGGCCAAGGGAACTTCGAACCGCTCATGGATCGCCTGCCGGGTAAGAAAGCCCAGATCACGGAGGCCATTGAT
G  V  R  E  R  L  R  G  A  A  E  A  T  S  A  Q  L  A  T  A  A  Y  N  T  S

GGCGTACGTGAACGCCTGCGTGGAGCTGCTGAAGCGACCTCTGCGCAGCTGGCCACAGCCGCCTACAATACTAGT
                                                                       SpeI
I  E  G  R  A  S  G  G  G  S  G  G  G  S  G  G  G  S  G  S  M  V  S  K  G
ATTGAAGGCCGTGCTAGCGGCGGTGGGTCTGGAGGCGGCTCAGGTGGTGGGTCGGGATCCATGGTGAGCAAGGGC
Xa             NheI     3xGGGS-Linker                   BamHI     YFP
E  E  L  F  T  G  V  V  P  I  L  V  E  L  D  G  D  V  N  G  H  K  F  S  V
GAGGAGCTGTTCACCGGGGTGGTGCCCATCCTGGTCGAGCTGGACGGCGACGTAAACGGCCACAAGTTCAGCGTG

S  G  E  G  E  G  D  A  T  Y  G  K  L  T  L  K  F  I  C  T  T  G  K  L  P
TCCGGCGAGGGCGAGGGCGATGCCACCTACGGCAAGCTGACCCTGAAGTTCATCTGCACCACCGGCAAGCTGCCC

V  P  W  P  T  L  V  T  T  F  G  Y  G  L  Q  C  F  A  R  Y  P  D  H  M  K
```

```
GTGCCCTGGCCCACCCTCGTGACCACCTTCGGCTACGGCCTGCAGTGCTTCGCCCGCTACCCCGACCACATGAAG

Q  H  D  F  F  K  S  A  M  P  E  G  Y  V  Q  E  R  T  I  F  F  K  D  D  G
CAGCACGACTTCTTCAAGTCCGCCATGCCCGAAGGCTACGTCCAGGAGCGCACCATCTTCTTCAAGGACGACGGC

N  Y  K  T  R  A  E  V  K  F  E  G  D  T  L  V  N  R  I  E  L  K  G  I  D
AACTACAAGACCCGCGCCGAGGTGAAGTTCGAGGGCGACACCCTGGTGAACCGCATCGAGCTGAAGGGCATCGAC

F  K  E  D  G  N  I  L  G  H  K  L  E  Y  N  Y  N  S  H  N  V  Y  I  M  A
TTCAAGGAGGACGGCAACATCCTGGGGCACAAGCTGGAGTACAACTACAACAGCCACAACGTCTATATCATGGCC

D  K  Q  K  N  G  I  K  V  N  F  K  I  R  H  N  I  E  D  G  S  V  Q  L  A
GACAAGCAGAAGAACGGCATCAAGGTGAACTTCAAGATCCGCCACAACATCGAGGACGGCAGCGTGCAGCTCGCC

D  H  Y  Q  Q  N  T  P  I  G  D  G  P  V  L  L  P  D  N  H  Y  L  S  Y  Q
GACCACTACCAGCAGAACACCCCCATCGGCGACGGCCCCGTGCTGCTGCCCGACAACCACTACCTGAGCTACCAG

S  K  L  S  K  D  P  N  E  K  R  D  H  M  V  L  L  E  F  V  T  A  A  G  I
TCCAAACTGAGCAAAGACCCCAACGAGAAGCGCGATCACATGGTCCTGCTGGAGTTCGTGACCGCCGCCGGGATC

T  L  G  M  D  E  L  Y  K  *
ACTCTCGGCATGGACGAGCTGTACAAGTAAGTCGACAAGCTTGCGGCCGCACTCGAG
                              SalI            NotI     XhoI
```

TDoT-L-mCherry

```
  M  I  I  N  E  T  A  D  D  I  V  Y  R  L  T  V  I  I  D  D  R  Y  E  S
CATATGATCATTAACGAAACTGCCGATGACATCGTTTATCGCCTGACAGTCATTATCGATGATCGCTACGAATCG
NdeI         TDoT
L  K  N  L  I  T  L  R  A  D  R  L  E  M  I  I  N  D  N  V  S  T  I  L  A
CTGAAAAACCTGATTACCTTACGTGCAGATCGCTTGGAGATGATCATCAATGACAATGTGTCCACCATTCTCGCG

S  I  T  S  I  E  G  R  A  S  G  G  G  S  G  G  G  S  G  G  G  S  G  S  M
AGCATTACTAGTATTGAAGGCCGTGCTAGCGGCGGTGGGTCTGGAGGCGGCTCAGGTGGTGGGTCGGGATCCATG
        SpeI    Xa        NheI      3xGGGS-Linker                 BamHI
V  S  K  G  E  E  D  N  M  A  I  I  K  E  F  M  R  F  K  V  H  M  E  G  S
GTGAGCAAGGGCGAGGAGGATAACATGGCCATCATCAAGGAGTTCATGCGCTTCAAGGTGCACATGGAGGGCTCC
mCherry
V  N  G  H  E  F  E  I  E  G  E  G  E  G  R  P  Y  E  G  T  Q  T  A  K  L
GTGAACGGCCACGAGTTCGAGATCGAGGGCGAGGGCGAGGGCCGCCCCTACGAGGGCACCCAGACCGCCAAGCTG

K  V  T  K  G  G  P  L  P  F  A  W  D  I  L  S  P  Q  F  M  Y  G  S  K  A
AAGGTGACCAAGGGTGGCCCCCTGCCCTTCGCCTGGGACATCCTGTCCCCTCAGTTCATGTACGGCTCCAAGGCC

Y  V  K  H  P  A  D  I  P  D  Y  L  K  L  S  F  P  E  G  F  K  W  E  R  V
TACGTGAAGCACCCCGCCGACATCCCCGACTACTTGAAGCTGTCCTTCCCCGAGGGCTTCAAGTGGGAGCGCGTG

M  N  F  E  D  G  G  V  V  T  V  T  Q  D  S  S  L  Q  D  G  E  F  I  Y  K
ATGAACTTCGAGGACGGCGGCGTGGTGACCGTGACCCAGGACTCCTCCTTGCAGGACGGCGAGTTCATCTACAAG

V  K  L  R  G  T  N  F  P  S  D  G  P  V  M  Q  K  K  T  M  G  W  E  A  S
GTGAAGCTGCGCGGCACCAACTTCCCCTCCGACGGCCCCGTAATGCAGAAGAAGACCATGGGCTGGGAGGCCTCC

S  E  R  M  Y  P  E  D  G  A  L  K  G  E  I  K  Q  R  L  K  L  K  D  G  G
TCCGAGCGGATGTACCCCGAGGACGGCGCCCTGAAGGGCGAGATCAAGCAGAGGCTGAAGCTGAAGGACGGCGGC

H  Y  D  A  E  V  K  T  T  Y  K  A  K  K  P  V  Q  L  P  G  A  Y  N  V  N
CACTACGACGCTGAGGTCAAGACCACCTACAAGGCCAAGAAGCCCGTGCAGCTGCCCGGCGCCTACAACGTCAAC

I  K  L  D  I  T  S  H  N  E  D  Y  T  I  V  E  Q  Y  E  R  A  E  G  R  H
ATCAAGTTGGACATCACCTCCCACAACGAGGACTACACCATCGTGGAACAGTACGAACGCGCCGAGGGCCGCCAC

S  T  G  G  M  D  E  L  Y  K  *
TCCACCGGCGGCATGGACGAGCTGTACAAGTAAGTCGACAAGCTTGCGGCCGCACTCGAG
                                  SalI            NotI     XhoI
```

TDoT-mCherry

M I I N E T A D D I V Y R L T V I I D D R Y E S
CATATGATCATTAACGAAACTGCCGATGACATCGTTTATCGCCTGACAGTCATTATCGATGATCGCTACGAATCG
*Nde*I TDoT
L K N L I T L R A D R L E M I I N D N V S T I L A
CTGAAAAACCTGATTACCTTACGTGCAGATCGCTTGGAGATGATCATCAATGACAATGTGTCCACCATTCTCGCG

S I G S M V S K G E E D N M A I I K E F M R F K V
AGCATTGGATCCATGGTGAGCAAGGGCGAGGAGGATAACATGGCCATCATCAAGGAGTTCATGCGCTTCAAGGTG
*Bam*HI mCherry
H M E G S V N G H E F E I E G E G E G R P Y E G T
CACATGGAGGGCTCCGTGAACGGCCACGAGTTCGAGATCGAGGGCGAGGGCGAGGGCCGCCCCTACGAGGGCACC

Q T A K L K V T K G G P L P F A W D I L S P Q F M
CAGACCGCCAAGCTGAAGGTGACCAAGGGTGGCCCCCTGCCCTTCGCCTGGGACATCCTGTCCCCTCAGTTCATG

Y G S K A Y V K H P A D I P D Y L K L S F P E G F
TACGGCTCCAAGGCCTACGTGAAGCACCCCGCCGACATCCCCGACTACTTGAAGCTGTCCTTCCCCGAGGGCTTC

K W E R V M N F E D G G V V T V T Q D S S L Q D G
AAGTGGGAGCGCGTGATGAACTTCGAGGACGGCGGCGTGGTGACCGTGACCCAGGACTCCTCCTTGCAGGACGGC

E F I Y K V K L R G T N F P S D G P V M Q K K T M
GAGTTCATCTACAAGGTGAAGCTGCGCGGCACCAACTTCCCCTCCGACGGCCCCGTAATGCAGAAGAAGACCATG

G W E A S S E R M Y P E D G A L K G E I K Q R L K
GGCTGGGAGGCCTCCTCCGAGCGGATGTACCCCGAGGACGGCGCCCTGAAGGGCGAGATCAAGCAGAGGCTGAAG

L K D G G H Y D A E V K T T Y K A K K P V Q L P G
CTGAAGGACGGCGGCCACTACGACGCTGAGGTCAAGACCACCTACAAGGCCAAGAAGCCCGTGCAGCTGCCCGGC

A Y N V N I K L D I T S H N E D Y T I V E Q Y E R
GCCTACAACGTCAACATCAAGTTGGACATCACCTCCCACAACGAGGACTACACCATCGTGGAACAGTACGAACGC

A E G R H S T G G M D E L Y K *
GCCGAGGGCCGCCACTCCACCGGCGGCATGGACGAGCTGTACAAGTAAGTCGACAAGCTTGCGGCCGCACTCGAG
*Sal*I *Not*I *Xho*I

3HAMP-L-mCherry

M G L F N A H A V A Q Q R A D R I A T L L Q S F
CATATGGGCCTGTTTAACGCCCATGCAGTTGCGCAGCAACGCGCGGATCGCATTGCGACTCTCCTGCAGTCCTTT
*Nde*I 3HAMP
A D G Q L D T A V G E A P A P G Y E R L Y D S L R

GCGGATGGTCAGTTGGACACCGCCGTGGGTGAAGCGCCAGCACCTGGTTACGAACGCCTGTATGACTCGCTTCGC
A L Q R Q L R E Q R A E L Q Q V E S L E A G L A E

GCCCTTCAGCGCCAACTGCGCGAACAACGTGCGGAGTTACAACAGGTTGAGAGCCTGGAAGCAGGCTTGGCTGAA
M S R Q H E A G W I D Q T I P A E R L E G R A A R

ATGAGTCGGCAGCATGAAGCAGGGTGGATTGACCAGACGATTCCGGCTGAACGGTTAGAGGGCCGTGCAGCACGT
I A K G V N E L V A A H I A V K M K V V S V V T A

ATCGCCAAAGGCGTGAATGAGCTGGTTGCTGCGCACATTGCGGTGAAAATGAAAGTCGTGAGCGTAGTCACCGCG
Y G Q G N F E P L M D R L P G K K A Q I T E A I D

TATGGCCAAGGGAACTTCGAACCGCTCATGGATCGCCTGCCGGGTAAGAAAGCCCAGATCACGGAGGCCATTGAT
G V R E R L R G A A E A T S A Q L A T A A Y N T S

```
GGCGTACGTGAACGCCTGCGTGGAGCTGCTGAAGCGACCTCTGCGCAGCTGGCCACAGCCGCCTACAATACTAGT
                                                                       SpeI
I  E  G  R  A  S  G  G  G  S  G  G  G  S  G  G  G  S  G  S  M  V  S  K  G
ATTGAAGGCCGTGCTAGCGGCGGTGGGTCTGGAGGCGGCTCAGGTGGTGGGTCGGGATCCATGGTGAGCAAGGGC
Xa          NheI    3xGGGS-Linker                    BamHI   mCherry
E  E  D  N  M  A  I  I  K  E  F  M  R  F  K  V  H  M  E  G  S  V  N  G  H
GAGGAGGATAACATGGCCATCATCAAGGAGTTCATGCGCTTCAAGGTGCACATGGAGGGCTCCGTGAACGGCCAC

E  F  E  I  E  G  E  G  E  G  R  P  Y  E  G  T  Q  T  A  K  L  K  V  T  K
GAGTTCGAGATCGAGGGCGAGGGCGAGGGCCGCCCCTACGAGGGCACCCAGACCGCCAAGCTGAAGGTGACCAAG

G  G  P  L  P  F  A  W  D  I  L  S  P  Q  F  M  Y  G  S  K  A  Y  V  K  H
GGTGGCCCCCTGCCCTTCGCCTGGGACATCCTGTCCCCTCAGTTCATGTACGGCTCCAAGGCCTACGTGAAGCAC

P  A  D  I  P  D  Y  L  K  L  S  F  P  E  G  F  K  W  E  R  V  M  N  F  E
CCCGCCGACATCCCCGACTACTTGAAGCTGTCCTTCCCCGAGGGCTTCAAGTGGGAGCGCGTGATGAACTTCGAG

D  G  G  V  V  T  V  T  Q  D  S  S  L  Q  D  G  E  F  I  Y  K  V  K  L  R
GACGGCGGCGTGGTGACCGTGACCCAGGACTCCTCCTTGCAGGACGGCGAGTTCATCTACAAGGTGAAGCTGCGC

G  T  N  F  P  S  D  G  P  V  M  Q  K  K  T  M  G  W  E  A  S  S  E  R  M
GGCACCAACTTCCCCTCCGACGGCCCCGTAATGCAGAAGAAGACCATGGGCTGGGAGGCCTCCTCCGAGCGGATG

Y  P  E  D  G  A  L  K  G  E  I  K  Q  R  L  K  L  K  D  G  G  H  Y  D  A

TACCCCGAGGACGGCGCCCTGAAGGGCGAGATCAAGCAGAGGCTGAAGCTGAAGGACGGCGGCCACTACGACGCT

E  V  K  T  T  Y  K  A  K  K  P  V  Q  L  P  G  A  Y  N  V  N  I  K  L  D
GAGGTCAAGACCACCTACAAGGCCAAGAAGCCCGTGCAGCTGCCCGGCGCCTACAACGTCAACATCAAGTTGGAC

I  T  S  H  N  E  D  Y  T  I  V  E  Q  Y  E  R  A  E  G  R  H  S  T  G  G
ATCACCTCCCACAACGAGGACTACACCATCGTGGAACAGTACGAACGCGCCGAGGGCCGCCACTCCACCGGCGGC

M  D  E  L  Y  K  *
ATGGACGAGCTGTACAAGTAAGTCGACAAGCTTGCGGCCGCACTCGAG
                     SalI        NotI     XhoI
```

*R*ADH

```
catatgtatcgtctgctgaataaaaccgcagttattaccggtggtaatagcggtattggt
 H  M  Y  R  L  L  N  K  T  A  V  I  T  G  G  N  S  G  I  G
  NdeI
ctggcaaccgcaaaacgttttgttgccgaaggtgcctatgtttttattgttggtcgtcgt
 L  A  T  A  K  R  F  V  A  E  G  A  Y  V  F  I  V  G  R  R

cgtaaagaactggaacaggcagcagcagaaattggtcgtaatgttaccgcagttaaagcc
 R  K  E  L  E  Q  A  A  A  E  I  G  R  N  V  T  A  V  K  A

gatgttaccaaactggaagatctggatcgtctgtatgcaattgttcgtgaacagcgtggt
 D  V  T  K  L  E  D  L  D  R  L  Y  A  I  V  R  E  Q  R  G

agcattgatgttctgtttgcaaatagcggtgccattgaacagaaaaccctggaagaaatt
 S  I  D  V  L  F  A  N  S  G  A  I  E  Q  K  T  L  E  E  I

acaccggaacattatgatcgcacctttgatgttaatgtgcgtggtctgatttttaccgtt
 T  P  E  H  Y  D  R  T  F  D  V  N  V  R  G  L  I  F  T  V

cagaaagcactgccgctgctgcgtgatggtggtagcgttattctgaccagcagcgttgcc
 Q  K  A  L  P  L  L  R  D  G  G  S  V  I  L  T  S  S  V  A

ggtgttctgggtctgcaggcacatgatacctatagcgcagcaaaagcagcagttcgtagc
 G  V  L  G  L  Q  A  H  D  T  Y  S  A  A  K  A  A  V  R  S
```

```
ctggcacgtacctggaccaccgaactgaaaggtcgtagcattcgtgttaatgcagttagt
 L  A  R  T  W  T  T  E  L  K  G  R  S  I  R  V  N  A  V  S

ccgggtgcaattgataccccgattattgaaaatcaggttagcacccaggaagaagcagac
 P  G  A  I  D  T  P  I  I  E  N  Q  V  S  T  Q  E  E  A  D

gaactgcgcgcaaaatttgcagcagcaacaccgctgggtcgtgttggtcgtccggaagaa
 E  L  R  A  K  F  A  A  A  T  P  L  G  R  V  G  R  P  E  E

ctggcagcagccgttctgtttctggcaagtgatgatagcagctatgttgcaggtattgaa
 L  A  A  A  V  L  F  L  A  S  D  D  S  S  Y  V  A  G  I  E

ctgtttgttgatggtggtctgacccaggtttaataactcgag
 L  F  V  D  G  G  L  T  Q  V  -  -  L  E
                                     XhoI
```

TDoT-L-*R*ADH

```
   M  I  I  N  E  T  A  D  D  I  V  Y  R  L  T  V  I  I  D  D  R  Y  E  S
CATATGATCATTAACGAAACTGCCGATGACATCGTTTATCGCCTGACAGTCATTATCGATGATCGCTACGAATCG
NdeI               TDoT
L  K  N  L  I  T  L  R  A  D  R  L  E  M  I  I  N  D  N  V  S  T  I  L  A
CTGAAAAACCTGATTACCTTACGTGCAGATCGCTTGGAGATGATCATCAATGACAATGTGTCCACCATTCTCGCG

S  I  T  S  I  E  G  R  A  S  G  G  G  S  G  G  G  S  G  G  G  S  G  S  M
AGCATTACTAGTATTGAAGGCCGTGCTAGCGGCGGTGGGTCTGGAGGCGGCTCAGGTGGTGGGTCGGGATCCATG
        SpeI         Xa         NheI    3xGGGS-Linker                 BamHI
Y  R  L  L  N  K  T  A  V  I  T  G  G  N  S  G  I  G  L  A  T  A  K  R  F
TATCGTCTGCTGAATAAAACCGCAGTTATTACCGGTGGTAATAGCGGTATTGGTCTGGCAACCGCAAAACGTTTT
RADH
V  A  E  G  A  Y  V  F  I  V  G  R  R  R  K  E  L  E  Q  A  A  A  E  I  G
GTTGCCGAAGGTGCCTATGTTTTTATTGTTGGTCGTCGTCGTAAAGAACTGGAACAGGCAGCAGCAGAAATTGGT

R  N  V  T  A  V  K  A  D  V  T  K  L  E  D  L  D  R  L  Y  A  I  V  R  E
CGTAATGTTACCGCAGTTAAAGCCGATGTTACCAAACTGGAAGATCTGGATCGTCTGTATGCAATTGTTCGTGAA

Q  R  G  S  I  D  V  L  F  A  N  S  G  A  I  E  Q  K  T  L  E  E  I  T  P
CAGCGTGGTAGCATTGATGTTCTGTTTGCAAATAGCGGTGCCATTGAACAGAAAACCCTGGAAGAAATTACACCG

E  H  Y  D  R  T  F  D  V  N  V  R  G  L  I  F  T  V  Q  K  A  L  P  L  L
GAACATTATGATCGCACCTTTGATGTTAATGTGCGTGGTCTGATTTTTACCGTTCAGAAAGCACTGCCGCTGCTG

R  D  G  G  S  V  I  L  T  S  S  V  A  G  V  L  G  L  Q  A  H  D  T  Y  S
CGTGATGGTGGTAGCGTTATTCTGACCAGCAGCGTTGCCGGTGTTCTGGGTCTGCAGGCACATGATACCTATAGC

A  A  K  A  A  V  R  S  L  A  R  T  W  T  T  E  L  K  G  R  S  I  R  V  N
GCAGCAAAAGCAGCAGTTCGTAGCCTGGCACGTACCTGGACCACCGAACTGAAAGGTCGTAGCATTCGTGTTAAT

A  V  S  P  G  A  I  D  T  P  I  I  E  N  Q  V  S  T  Q  E  E  A  D  E  L
GCAGTTAGTCCGGGTGCAATTGATACCCCGATTATTGAAAATCAGGTTAGCACCCAGGAAGAAGCAGACGAACTG

R  A  K  F  A  A  A  T  P  L  G  R  V  G  R  P  E  E  L  A  A  A  V  L  F
CGCGCAAAATTTGCAGCAGCAACACCGCTGGGTCGTGTTGGTCGTCCGGAAGAACTGGCAGCAGCCGTTCTGTTT

L  A  S  D  D  S  S  Y  V  A  G  I  E  L  F  V  D  G  G  L  T  Q  V  *
CTGGCAAGTGATGATAGCAGCTATGTTGCAGGTATTGAACTGTTTGTTGATGGTGGTCTGACCCAGGTTTAAGTC
                                                                        SalI

GACAAGCTTGCGGCCGCACTCGAG
          NotI    XhoI
```

3HAMP-L-*R*ADH

```
   M   G   L   F   N   A   H   A   V   A   Q   Q   R   A   D   R   I   A   T   L   L   Q   S   F
CATATGGGCCTGTTTAACGCCCATGCAGTTGCGCAGCAACGCGCGGATCGCATTGCGACTCTCCTGCAGTCCTTT
NdeI            3Hamp
A   D   G   Q   L   D   T   A   V   G   E   A   P   A   P   G   Y   E   R   L   Y   D   S   L   R
GCGGATGGTCAGTTGGACACCGCCGTGGGTGAAGCGCCAGCACCTGGTTACGAACGCCTGTATGACTCGCTTCGC

A   L   Q   R   Q   L   R   E   Q   R   A   E   L   Q   Q   V   E   S   L   E   A   G   L   A   E
GCCCTTCAGCGCCAACTGCGCGAACAACGTGCGGAGTTACAACAGGTTGAGAGCCTGGAAGCAGGCTTGGCTGAA

M   S   R   Q   H   E   A   G   W   I   D   Q   T   I   P   A   E   R   L   E   G   R   A   A   R
ATGAGTCGGCAGCATGAAGCAGGGTGGATTGACCAGACGATTCCGGCTGAACGGTTAGAGGGCCGTGCAGCACGT

I   A   K   G   V   N   E   L   V   A   A   H   I   A   V   K   M   K   V   V   S   V   V   T   A
ATCGCCAAAGGCGTGAATGAGCTGGTTGCTGCGCACATTGCGGTGAAAATGAAAGTCGTGAGCGTAGTCACCGCG

Y   G   Q   G   N   F   E   P   L   M   D   R   L   P   G   K   K   A   Q   I   T   E   A   I   D
TATGGCCAAGGGAACTTCGAACCGCTCATGGATCGCCTGCCGGGTAAGAAAGCCCAGATCACGGAGGCCATTGAT

G   V   R   E   R   L   R   G   A   A   E   A   T   S   A   Q   L   A   T   A   A   Y   N   T   S
GGCGTACGTGAACGCCTGCGTGGAGCTGCTGAAGCGACCTCTGCGCAGCTGGCCACAGCCGCCTACAATACTAGT
                                                                        SpeI
I   E   G   R   A   S   G   G   G   S   G   G   G   S   G   G   G   S   G   S   M   Y   R   L   L
ATTGAAGGCCGTGCTAGCGGCGGTGGGTCTGGAGGCGGCTCAGGTGGTGGGTCGGGATCCATGTATCGTCTGCTG
     Xa        NheI     3xGGGS-Linker                     BamHI
N   K   T   A   V   I   T   G   G   N   S   G   I   G   L   A   T   A   K   R   F   V   A   E   G
AATAAAACCGCAGTTATTACCGGTGGTAATAGCGGTATTGGTCTGGCAACCGCAAAACGTTTTGTTGCCGAAGGT
RADH
A   Y   V   F   I   V   G   R   R   R   K   E   L   E   Q   A   A   A   E   I   G   R   N   V   T
GCCTATGTTTTTATTGTTGGTCGTCGTCGTAAAGAACTGGAACAGGCAGCAGCAGAAATTGGTCGTAATGTTACC

A   V   K   A   D   V   T   K   L   E   D   L   D   R   L   Y   A   I   V   R   E   Q   R   G   S
GCAGTTAAAGCCGATGTTACCAAACTGGAAGATCTGGATCGTCTGTATGCAATTGTTCGTGAACAGCGTGGTAGC

I   D   V   L   F   A   N   S   G   A   I   E   Q   K   T   L   E   E   I   T   P   E   H   Y   D
ATTGATGTTCTGTTTGCAAATAGCGGTGCCATTGAACAGAAAACCCTGGAAGAAATTACACCGGAACATTATGAT

R   T   F   D   V   N   V   R   G   L   I   F   T   V   Q   K   A   L   P   L   L   R   D   G   G
CGCACCTTTGATGTTAATGTGCGTGGTCTGATTTTTACCGTTCAGAAAGCACTGCCGCTGCTGCGTGATGGTGGT

S   V   I   L   T   S   S   V   A   G   V   L   G   L   Q   A   H   D   T   Y   S   A   A   K   A
AGCGTTATTCTGACCAGCAGCGTTGCCGGTGTTCTGGGTCTGCAGGCACATGATACCTATAGCGCAGCAAAAGCA

A   V   R   S   L   A   R   T   W   T   T   E   L   K   G   R   S   I   R   V   N   A   V   S   P
GCAGTTCGTAGCCTGGCACGTACCTGGACCACCGAACTGAAAGGTCGTAGCATTCGTGTTAATGCAGTTAGTCCG

G   A   I   D   T   P   I   I   E   N   Q   V   S   T   Q   E   E   A   D   E   L   R   A   K   F
GGTGCAATTGATACCCCGATTATTGAAAATCAGGTTAGCACCCAGGAAGAAGCAGACGAACTGCGCGCAAAATTT

A   A   A   T   P   L   G   R   V   G   R   P   E   E   L   A   A   A   V   L   F   L   A   S   D
GCAGCAGCAACACCGCTGGGTCGTGTTGGTCGTCCGGAAGAACTGGCAGCAGCCGTTCTGTTTCTGGCAAGTGAT

D   S   S   Y   V   A   G   I   E   L   F   V   D   G   G   L   T   Q   V   *
GATAGCAGCTATGTTGCAGGTATTGAACTGTTTGTTGATGGTGGTCTGACCCAGGTTTAAGTCGACAAGCTTGCG
                                                              SalI
GCCGCACTCGAG
NotI  XhoI
```

*Lb*ADH

CATATGTCTAACCGTTTGGATGGTAAGGTAGCAATCATTACAGGTGGTACGTTGGGTATCGGTTTAGCTATCGCC
M S N R L D G K V A I I T G G T L G I G L A I A
NdeI

ACGAAGTTCGTTGAAGAAGGGGCTAAGGTCATGATTACCGGCCGGCACAGCGATGTTGGTGAAAAAGCAGCTAAG
T K F V E E G A K V M I T G R H S D V G E K A A K

AGTGTCGGCACTCCTGATCAGATTCAATTTTTCCAACATGATTCTTCCGATGAAGACGGCTGGACGAAATTATTC
S V G T P D Q I Q F F Q H D S S D E D G W T K L F

GATGCAACGGAAAAAGCCTTTGGCCCAGTTTCTACATTAGTTAATAACGCTGGGATCGCGGTTAACAAGAGTGTC
D A T E K A F G P V S T L V N N A G I A V N K S V

GAAGAAACCACGACTGCTGAATGGCGTAAATTATTAGCCGTCAACCTTGATGGTGTCTTCTTCGGTACCCGATTA
E E T T T A E W R K L L A V N L D G V F F G T R L

GGGATTCAACGGATGAAGAACAAAGGCTTAGGGGCTTCCATCATCAACATGTCTTCGATCGAAGGCTTTGTGGGT
G I Q R M K N K G L G A S I I N M S S I E G F V G

GATCCTAGCTTAGGGGCTTACAACGCATCTAAAGGGGCCGTACGGATTATGTCCAAGTCAGCTGCCTTAGATTGT
D P S L G A Y N A S K G A V R I M S K S A A L D C

GCCCTAAAGGACTACGATGTTCGGGTAAACACTGTTCACCCTGGCTACATCAAGACACCATTGGTTGATGACCTA
A L K D Y D V R V N T V H P G Y I K T P L V D D L

CCAGGGGCCGAAGAAGCGATGTCACAACGGACCAAGACGCCAATGGGCCATATCGGTGAACCTAACGATATTGCC
P G A E E A M S Q R T K T P M G H I G E P N D I A

TACATCTGTGTTTACTTGGCTTCTAACGAATCTAAATTTGCAACGGGTTCTGAATTCGTAGTTGACGGTGGCTAC
Y I C V Y L A S N E S K F A T G S E F V V D G G Y

ACTGCTCAATAGTAGCGCGGATCCGAATTCGAGCTCCGTCGACAAGCTTGCGGCCGC
T A Q * * **R G S E F E L R R Q A C G R**
BamHI ***SalI*** ***NotI***

TDoT-L-*Lb*ADH

CATATGATCATTAACGAAACTGCCGATGACATCGTTTATCGCCTGACAGTCATTATCGATGATCGCTACGAATCG
M I I N E T A D D I V Y R L T V I I D D R Y E S
NdeI TDoT

CTGAAAAACCTGATTACCTTACGTGCAGATCGCTTGGAGATGATCATCAATGACAATGTGTCCACCATTCTCGCG
L K N L I T L R A D R L E M I I N D N V S T I L A

AGCATTACTAGTATTGAAGGCCGTGCTAGCGGCGGTGGGTCTGGAGGCGGCTCAGGTGGTGGGTCGGGATCCATG
S I **T S** I E G R **A S** G G G S G G G S G G G S **G S** M
SpeI **Xa** ***NheI*** **3xGGGS-Linker** ***BamHI***

TCTAACCGTTTGGATGGTAAGGTAGCAATCATTACAGGTGGTACGTTGGGTATCGGTTTAGCTATCGCCACGAAG
S N R L D G K V A I I T G G T L G I G L A I A T K
***Lb*ADH**

TTCGTTGAAGAAGGGGCTAAGGTCATGATTACCGGCCGGCACAGCGATGTTGGTGAAAAAGCAGCTAAGAGTGTC
F V E E G A K V M I T G R H S D V G E K A A K S V

GGCACTCCTGATCAGATTCAATTTTTCCAACATGATTCTTCCGATGAAGACGGCTGGACGAAATTATTCGATGCA
G T P D Q I Q F F Q H D S S D E D G W T K L F D A

ACGGAAAAAGCCTTTGGCCCAGTTTCTACATTAGTTAATAACGCTGGGATCGCGGTTAACAAGAGTGTCGAAGAA
T E K A F G P V S T L V N N A G I A V N K S V E E

ACCACGACTGCTGAATGGCGTAAATTATTAGCCGTCAACCTTGATGGTGTCTTCTTCGGTACCCGATTAGGGATT

T T T A E W R K L L A V N L D G V F F G T R L G I

CAACGGATGAAGAACAAAGGCTTAGGGGCTTCCATCATCAACATGTCTTCGATCGAAGGCTTTGTGGGTGATCCT
Q R M K N K G L G A S I I N M S S I E G F V G D P

AGCTTAGGGGCTTACAACGCATCTAAAGGGGCCGTACGGATTATGTCCAAGTCAGCTGCCTTAGATTGTGCCCTA
S L G A Y N A S K G A V R I M S K S A A L D C A L

AAGGACTACGATGTTCGGGTAAACACTGTTCACCCTGGCTACATCAAGACACCATTGGTTGATGACCTACCAGGG
K D Y D V R V N T V H P G Y I K T P L V D D L P G

GCCGAAGAAGCGATGTCACAACGGACCAAGACGCCAATGGGCCATATCGGTGAACCTAACGATATTGCCTACATC
A E E A M S Q R T K T P M G H I G E P N D I A Y I

TGTGTTTACTTGGCTTCTAACGAATCTAAATTTGCAACGGGTTCTGAATTCGTAGTTGACGGTGGCTACACTGCT
C V Y L A S N E S K F A T G S E F V V D G G Y T A

CAATAGAGTCGACAAGCTTGCGGCCGCACTCGA
Q * S R Q A C G R T R
*Sal*I *Not*I

3HAMP-L-*Lb*ADH

CATATGGGCCTGTTTAACGCCCATGCAGTTGCGCAGCAACGCGCGGATCGCATTGCGACTCTCCTGCAGTCCTTT
M G L F N A H A V A Q Q R A D R I A T L L Q S F
*Nde*I 3HAMP
GCGGATGGTCAGTTGGACACCGCCGTGGGTGAAGCGCCAGCACCTGGTTACGAACGCCTGTATGACTCGCTTCGC
A D G Q L D T A V G E A P A P G Y E R L Y D S L R

GCCCTTCAGCGCCAACTGCGCGAACAACGTGCGGAGTTACAACAGGTTGAGAGCCTGGAAGCAGGCTTGGCTGAA
A L Q R Q L R E Q R A E L Q Q V E S L E A G L A E

ATGAGTCGGCAGCATGAAGCAGGGTGGATTGACCAGACGATTCCGGCTGAACGGTTAGAGGGCCGTGCAGCACGT
M S R Q H E A G W I D Q T I P A E R L E G R A A R

ATCGCCAAAGGCGTGAATGAGCTGGTTGCTGCGCACATTGCGGTGAAAATGAAAGTCGTGAGCGTAGTCACCGCG
I A K G V N E L V A A H I A V K M K V V S V V T A

TATGGCCAAGGGAACTTCGAACCGCTCATGGATCGCCTGCCGGGTAAGAAAGCCCAGATCACGGAGGCCATTGAT
Y G Q G N F E P L M D R L P G K K A Q I T E A I D

GGCGTACGTGAACGCCTGCGTGGAGCTGCTGAAGCGACCTCTGCGCAGCTGGCCACAGCCGCCTACAATACTAGT
G V R E R L R G A A E A T S A Q L A T A A Y N T S
*Spe*I
ATTGAAGGCCGTGCTAGCGGCGGTGGGTCTGGAGGCGGCTCAGGTGGTGGGTCGGGATCCATGTCTAACCGTTTG
I E G R A S G G G S G G G S G G G S G S M S N R L
Xa *Nhe*I 3xGGGS-Linker *Bam*HI *Lb*ADH
GATGGTAAGGTAGCAATCATTACAGGTGGTACGTTGGGTATCGGTTTAGCTATCGCCACGAAGTTCGTTGAAGAA
D G K V A I I T G G T L G I G L A I A T K F V E E

GGGGCTAAGGTCATGATTACCGGCCGGCACAGCGATGTTGGTGAAAAAGCAGCTAAGAGTGTCGGCACTCCTGAT
G A K V M I T G R H S D V G E K A A K S V G T P D

CAGATTCAATTTTTCCAACATGATTCTTCCGATGAAGACGGCTGGACGAAATTATTCGATGCAACGGAAAAAGCC
Q I Q F F Q H D S S D E D G W T K L F D A T E K A

TTTGGCCCAGTTTCTACATTAGTTAATAACGCTGGGATCGCGGTTAACAAGAGTGTCGAAGAAACCACGACTGCT
F G P V S T L V N N A G I A V N K S V E E T T T A

GAATGGCGTAAATTATTAGCCGTCAACCTTGATGGTGTCTTCTTCGGTACCCGATTAGGGATTCAACGGATGAAG

```
 E  W  R  K  L  L  A  V  N  L  D  G  V  F  F  G  T  R  L  G  I  Q  R  M  K

AACAAAGGCTTAGGGGCTTCCATCATCAACATGTCTTCGATCGAAGGCTTTGTGGGTGATCCTAGCTTAGGGGCT
 N  K  G  L  G  A  S  I  I  N  M  S  S  I  E  G  F  V  G  D  P  S  L  G  A

TACAACGCATCTAAAGGGGCCGTACGGATTATGTCCAAGTCAGCTGCCTTAGATTGTGCCCTAAAGGACTACGAT
 Y  N  A  S  K  G  A  V  R  I  M  S  K  S  A  A  L  D  C  A  L  K  D  Y  D

GTTCGGGTAAACACTGTTCACCCTGGCTACATCAAGACACCATTGGTTGATGACCTACCAGGGGCCGAAGAAGCG
 V  R  V  N  T  V  H  P  G  Y  I  K  T  P  L  V  D  D  L  P  G  A  E  E  A

ATGTCACAACGGACCAAGACGCCAATGGGCCATATCGGTGAACCTAACGATATTGCCTACATCTGTGTTTACTTG
 M  S  Q  R  T  K  T  P  M  G  H  I  G  E  P  N  D  I  A  Y  I  C  V  Y  L

GCTTCTAACGAATCTAAATTTGCAACGGGTTCTGAATTCGTAGTTGACGGTGGCTACACTGCTCAATAGAGTCGA
 A  S  N  E  S  K  F  A  T  G  S  E  F  V  V  D  G  G  Y  T  A  Q  *  S  R
                                                                      SalI
CAAGCTTGCGGCCGCACTCGA
 Q  A  C  G  R  T  R
          NotI
```

_Pf_BAL-His$_6$

```
ATGGCGATGATTACAGGCGGCGAACTGGTTGTTCGCACCCTAATAAAGGCTGGGGTCGAACATCTGTTCGGCCTG
 M  A  M  I  T  G  G  E  L  V  V  R  T  L  I  K  A  G  V  E  H  L  F  G  L

CACGGCGCGCATATCGATACGATTTTTCAAGCCTGTCTCGATCATGATGTGCCGATCATCGACACCCGCCATGAG
 H  G  A  H  I  D  T  I  F  Q  A  C  L  D  H  D  V  P  I  I  D  T  R  H  E

GCCGCCGCAGGGCATGCGGCCGAGGGCTATGCCCGCGCTGGCGCCAAGCTGGGCGTGGCGCTGGTCACGGCGGGC
 A  A  A  G  H  A  A  E  G  Y  A  R  A  G  A  K  L  G  V  A  L  V  T  A  G

GGGGGATTTACCAATGCGGTCACGCCCATTGCCAACGCTTGGCTGGATCGCACGCCGGTGCTCTTCCTCACCGGA
 G  G  F  T  N  A  V  T  P  I  A  N  A  W  L  D  R  T  P  V  L  F  L  T  G

TCGGGCGCGCTGCGTGATGATGAAACCAACACGTTGCAGGCGGGGATTGATCAGGTCGCCATGGCGGCGCCCATT
 S  G  A  L  R  D  D  E  T  N  T  L  Q  A  G  I  D  Q  V  A  M  A  A  P  I

ACCAAATGGGCGCATCGGGTGATGGCAACCGAGCATATCCCACGGCTGGTGATGCAGGCGATCCGCGCCGCGTTG
 T  K  W  A  H  R  V  M  A  T  E  H  I  P  R  L  V  M  Q  A  I  R  A  A  L

AGCGCGCCACGCGGGCCGGTGTTGCTGGATCTGCCGTGGGATATTCTGATGAACCAGATTGATGAGGATAGCGTC
 S  A  P  R  G  P  V  L  L  D  L  P  W  D  I  L  M  N  Q  I  D  E  D  S  V

ATTATCCCCGATCTGGTCTTGTCCGCGCATGGGGCCAGACCCGACCCTGCCGATCTGGATCAGGCTCTCGCGCTT
 I  I  P  D  L  V  L  S  A  H  G  A  R  P  D  P  A  D  L  D  Q  A  L  A  L

TTGCGCAAGGCGGAGCGGCCGGTCATCGTGCTCGGCTCAGAAGCCTCGCGGACAGCGCGCAAGACGGCGCTTAGC
 L  R  K  A  E  R  P  V  I  V  L  G  S  E  A  S  R  T  A  R  K  T  A  L  S

GCCTTCGTGGCGGCGACTGGCGTGCCGGTGTTTGCCGATTATGAAGGGCTAAGCATGCTCTCGGGGCTGCCCGAT
 A  F  V  A  A  T  G  V  P  V  F  A  D  Y  E  G  L  S  M  L  S  G  L  P  D

GCTATGCGGGGCGGGCTGGTGCAAAACCTCTATTCTTTTGCCAAAGCCGATGCCGCGCCAGATCTCGTGCTGATG
 A  M  R  G  G  L  V  Q  N  L  Y  S  F  A  K  A  D  A  A  P  D  L  V  L  M

CTGGGGGCGCGCTTTGGCCTTAACACCGGGCATGGATCTGGGCAGTTGATCCCCCATAGCGCGCAGGTCATTCAG
 L  G  A  R  F  G  L  N  T  G  H  G  S  G  Q  L  I  P  H  S  A  Q  V  I  Q

GTCGACCCTGATGCCTGCGAGCTGGGACGCCTGCAGGGCATCGCTCTGGGCATTGTGGCCGATGTGGGTGGGACC
```

```
 V  D  P  D  A  C  E  L  G  R  L  Q  G  I  A  L  G  I  V  A  D  V  G  G  T

ATCGAGGCTTTGGCGCAGGCCACCGCGCAAGATGCGGCTTGGCCGGATCGCGGCGACTGGTGCGCCAAAGTGACG
 I  E  A  L  A  Q  A  T  A  Q  D  A  A  W  P  D  R  G  D  W  C  A  K  V  T

GATCTGGCGCAAGAGCGCTATGCCAGCATCGCTGCGAAATCGAGCAGCGAGCATGCGCTCCACCCCTTTCACGCC
 D  L  A  Q  E  R  Y  A  S  I  A  A  K  S  S  S  E  H  A  L  H  P  F  H  A

TCGCAGGTCATTGCCAAACACGTCGATGCAGGGGTGACGGTGGTAGCGGATGGTGCGCTGACCTATCTCTGGCTG
 S  Q  V  I  A  K  H  V  D  A  G  V  T  V  V  A  D  G  A  L  T  Y  L  W  L

TCCGAAGTGATGAGCCGCGTGAAACCCGGCGGTTTTCTCTGCCACGGCTATCTAGGCTCGATGGGCGTGGGCTTC
 S  E  V  M  S  R  V  K  P  G  G  F  L  C  H  G  Y  L  G  S  M  G  V  G  F

GGCACGGCGCTGGGCGCGCAAGTGGCCGATCTTGAAGCAGGCCGCCGCACGATCCTTGTGACCGGCGATGGCTCG
 G  T  A  L  G  A  Q  V  A  D  L  E  A  G  R  R  T  I  L  V  T  G  D  G  S

GTGGGCTATAGCATCGGTGAATTTGATACGCTGGTGCGCAAACAATTGCCGCTGATCGTCATCATCATGAACAAC
 V  G  Y  S  I  G  E  F  D  T  L  V  R  K  Q  L  P  L  I  V  I  I  M  N  N

CAAAGCTGGGGGGCGACATTGCATTTCCAGCAATTGGCCGTCGGCCCCAATCGCGTGACGGGCACCCGTTTGGAA
 Q  S  W  G  A  T  L  H  F  Q  Q  L  A  V  G  P  N  R  V  T  G  T  R  L  E

AATGGCTCCTATCACGGGGTGGCCGCCGCCTTTGGCGCGGATGGCTATCATGTCGACAGTGTGGAGAGCTTTTCT
 N  G  S  Y  H  G  V  A  A  A  F  G  A  D  G  Y  H  V  D  S  V  E  S  F  S

GCGGCTCTGGCCCAAGCGCTCGCCCATAATCGCCCCGCCTGCATCAATGTCGCGGTCGCGCTCGATCCGATCCCG
 A  A  L  A  Q  A  L  A  H  N  R  P  A  C  I  N  V  A  V  A  L  D  P  I  P

CCCGAAGAACTCATTCTGATCGGCATGGACCCCTTCGGATCTCATCACCATCACCATCACTAAGCTTCT
 P  E  E  L  I  L  I  G  M  D  P  F  G  S  H  H  H  H  H  H  *  A  S
                                                 His-tag           HindIII
```

TDoT-L-*Pf*BAL

```
   M  I  I  N  E  T  A  D  D  I  V  Y  R  L  T  V  I  I  D  D  R  Y  E  S
CATATGATCATTAACGAAACTGCCGATGACATCGTTTATCGCCTGACAGTCATTATCGATGATCGCTACGAATCG
NdeI          TDoT
L  K  N  L  I  T  L  R  A  D  R  L  E  M  I  I  N  D  N  V  S  T  I  L  A
CTGAAAAACCTGATTACCTTACGTGCAGATCGCTTGGAGATGATCATCAATGACAATGTGTCCACCATTCTCGCG

S  I  T  S  I  E  G  R  A  S  G  G  G  S  G  G  G  S  G  G  G  S  G  S  M
AGCATTACTAGTATTGAAGGCCGTGCTAGCGGCGGTGGGTCTGGAGGCGGCTCAGGTGGTGGGTCGGGATCCATG
       SpeI        Xa         NheI    3xGGGS-Linker                   BamHI
A  M  I  T  G  G  E  L  V  V  R  T  L  I  K  A  G  V  E  H  L  F  G  L  H
GCGATGATTACAGGCGGCGAACTGGTTGTTCGCACCCTAATAAAGGCTGGGGTCGAACATCTGTTCGGCCTGCAC
PfBAL
G  A  H  I  D  T  I  F  Q  A  C  L  D  H  D  V  P  I  I  D  T  R  H  E  A
GGCGCGCATATCGATACGATTTTTCAAGCCTGTCTCGATCATGATGTGCCGATCATCGACACCCGCCATGAGGCC

A  A  G  H  A  A  E  G  Y  A  R  A  G  A  K  L  G  V  A  L  V  T  A  G  G
GCCGCAGGGCATGCGGCCGAGGGCTATGCCCGCGCTGGCGCCAAGCTGGGCGTGGCGCTGGTCACGGCGGGCGGG

G  F  T  N  A  V  T  P  I  A  N  A  W  L  D  R  T  P  V  L  F  L  T  G  S
GGATTTACCAATGCGGTCACGCCCATTGCCAACGCTTGGCTGGATCGCACGCCGGTGCTCTTCCTCACCGGATCG

G  A  L  R  D  D  E  T  N  T  L  Q  A  G  I  D  Q  V  A  M  A  A  P  I  T
GGCGCGCTGCGTGATGATGAAACCAACACGTTGCAGGCGGGGATTGATCAGGTCGCCATGGCGGCGCCCATTACC

K  W  A  H  R  V  M  A  T  E  H  I  P  R  L  V  M  Q  A  I  R  A  A  L  S
AAATGGGCGCATCGGGTGATGGCAACCGAGCATATCCCACGGCTGGTGATGCAGGCGATCCGCGCCGCGTTGAGC
```

```
A   P   R   G   P   V   L   L   D   L   P   W   D   I   L   M   N   Q   I   D   E   D   S   V   I
GCGCCACGCGGGCCGGTGTTGCTGGATCTGCCGTGGGATATTCTGATGAACCAGATTGATGAGGATAGCGTCATT

I   P   D   L   V   L   S   A   H   G   A   R   P   D   P   A   D   L   D   Q   A   L   A   L   L
ATCCCCGATCTGGTCTTGTCCGCACATGGGGCCAGACCCGACCCTGCCGATCTGGATCAGGCTCTCGCGCTTTTG

R   K   A   E   R   P   V   I   V   L   G   S   E   A   S   R   T   A   R   K   T   A   L   S   A
CGCAAGGCGGAGCGGCCGGTCATCGTGCTCGGCTCAGAAGCCTCGCGGACAGCGCGCAAGACGGCGCTTAGCGCA

F   V   A   A   T   G   V   P   V   F   A   D   Y   E   G   L   S   M   L   S   G   L   P   D   A
TTCGTGGCGGCGACTGGCGTGCCGGTGTTTGCCGATTATGAAGGGCTAAGCATGCTCTCGGGGCTGCCCGATGCT

M   R   G   G   L   V   Q   N   L   Y   S   F   A   K   A   D   A   A   P   D   L   V   L   M   L
ATGCGGGGCGGGCTGGTGCAAAACCTCTATTCTTTTGCCAAAGCCGATGCCGCGCCAGATCTCGTGCTGATGCTG

G   A   R   F   G   L   N   T   G   H   G   S   G   Q   L   I   P   H   S   A   Q   V   I   Q   V
GGGGCGCGCTTTGGCCTTAACACCGGGCATGGATCTGGGCAGTTGATCCCCCATAGCGCGCAGGTCATTCAGGTC

D   P   D   A   C   E   L   G   R   L   Q   G   I   A   L   G   I   V   A   D   V   G   G   T   I
GACCCTGATGCCTGCGAGCTGGGACGCCTGCAGGGCATCGCTCTGGGCATTGTGGCCGATGTGGGTGGGACCATC

E   A   L   A   Q   A   T   A   Q   D   A   A   W   P   D   R   G   D   W   C   A   K   V   T   D
GAGGCTTTGGCGCAGGCCACCGCGCAAGATGCGGCTTGGCCGGATCGCGGCGACTGGTGCGCCAAAGTGACGGAT

L   A   Q   E   R   Y   A   S   I   A   A   K   S   S   S   E   H   A   L   H   P   F   H   A   S
CTGGCGCAAGAGCGCTATGCCAGCATCGCTGCGAAATCGAGCAGCGAGCATGCGCTCCACCCCTTTCACGCCTCG

Q   V   I   A   K   H   V   D   A   G   V   T   V   V   A   D   G   A   L   T   Y   L   W   L   S
CAGGTCATTGCCAAACACGTCGATGCAGGGGTGACGGTGGTAGCGGATGGTGCGCTGACCTATCTCTGGCTGTCC

E   V   M   S   R   V   K   P   G   G   F   L   C   H   G   Y   L   G   S   M   G   V   G   F   G
GAAGTGATGAGCCGCGTGAAACCCGGCGGTTTTCTCTGCCACGGCTATCTAGGCTCGATGGGCGTGGGCTTCGGC

T   A   L   G   A   Q   V   A   D   L   E   A   G   R   R   T   I   L   V   T   G   D   G   S   V
ACGGCGCTGGGCGCGCAAGTGGCCGATCTTGAAGCAGGCCGCCGCACGATCCTTGTGACCGGCGATGGCTCGGTG

G   Y   S   I   G   E   F   D   T   L   V   R   K   Q   L   P   L   I   V   I   I   M   N   N   Q
GGCTATAGCATCGGTGAATTTGATACGCTGGTGCGCAAACAATTGCCGCTGATCGTCATCATCATGAACAACCAA

S   W   G   A   T   L   H   F   Q   Q   L   A   V   G   P   N   R   V   T   G   T   R   L   E   N
AGCTGGGGGGCGACATTGCATTTCCAGCAATTGGCCGTCGGCCCCAATCGCGTGACGGGCACCCGTTTGGAAAAT

G   S   Y   H   G   V   A   A   A   F   G   A   D   G   Y   H   V   D   S   V   E   S   F   S   A
GGCTCCTATCACGGGGTGGCCGCCGCCTTTGGCGCGGATGGCTATCATGTCGACAGTGTGGAGAGCTTTTCTGCG

A   L   A   Q   A   L   A   H   N   R   P   A   C   I   N   V   A   V   A   L   D   P   I   P   P
GCTCTGGCCCAAGCGCTCGCCCATAATCGCCCCGCCTGCATCAATGTCGCGGTCGCGCTCGATCCGATCCCGCCC

E   E   L   I   L   I   G   M   D   P   F   A   *
GAAGAACTCATTCTGATCGGCATGGACCCCTTCGCATAAGCGGCCGCACTCGAG
                                       --------- ------
                                         NotI     XhoI
```

3HAMP-L-*Pf*BAL

```
    M   G   L   F   N   A   H   A   V   A   Q   Q   R   A   D   R   I   A   T   L   L   Q   S   F
CATATGGGCCTGTTTAACGCCCATGCAGTTGCGCAGCAACGCGCGGATCGCATTGCGACTCTCCTGCAGTCCTTT
------
NdeI         3HAMP
A   D   G   Q   L   D   T   A   V   G   E   A   P   A   P   G   Y   E   R   L   Y   D   S   L   R

GCGGATGGTCAGTTGGACACCGCCGTGGGTGAAGCGCCAGCACCTGGTTACGAACGCCTGTATGACTCGCTTCGC
A   L   Q   R   Q   L   R   E   Q   R   A   E   L   Q   Q   V   E   S   L   E   A   G   L   A   E

GCCCTTCAGCGCCAACTGCGCGAACAACGTGCGGAGTTACAACAGGTTGAGAGCCTGGAAGCAGGCTTGGCTGAA
M   S   R   Q   H   E   A   G   W   I   D   Q   T   I   P   A   E   R   L   E   G   R   A   A   R
```

```
ATGAGTCGGCAGCATGAAGCAGGGTGGATTGACCAGACGATTCCGGCTGAACGGTTAGAGGGCCGTGCAGCACGT
I  A  K  G  V  N  E  L  V  A  A  H  I  A  V  K  M  K  V  V  S  V  V  T  A

ATCGCCAAAGGCGTGAATGAGCTGGTTGCTGCGCACATTGCGGTGAAAATGAAAGTCGTGAGCGTAGTCACCGCG
Y  G  Q  G  N  F  E  P  L  M  D  R  L  P  G  K  K  A  Q  I  T  E  A  I  D

TATGGCCAAGGGAACTTCGAACCGCTCATGGATCGCCTGCCGGGTAAGAAAGCCCAGATCACGGAGGCCATTGAT
G  V  R  E  R  L  R  G  A  A  E  A  T  S  A  Q  L  A  T  A  A  Y  N  T  S

GGCGTACGTGAACGCCTGCGTGGAGCTGCTGAAGCGACCTCTGCGCAGCTGGCCACAGCCGCCTACAATACTAGT
                                                                     SpeI
I  E  G  R  A  S  G  G  G  S  G  G  G  S  G  G  G  S  G  S  M  A  M  I  T
ATTGAAGGCCGTGCTAGCGGCGGTGGGTCTGGAGGCGGCTCAGGTGGTGGGTCGGGATCCATGGCGATGATTACA
Xa           NheI   3xGGGS-Linker                       BamHI   PfBAL
G  G  E  L  V  V  R  T  L  I  K  A  G  V  E  H  L  F  G  L  H  G  A  H  I
GGCGGCGAACTGGTTGTTCGCACCCTAATAAAGGCTGGGGTCGAACATCTGTTCGGCCTGCACGGCGCGCATATC

D  T  I  F  Q  A  C  L  D  H  D  V  P  I  I  D  T  R  H  E  A  A  A  G  H
GATACGATTTTTCAAGCCTGTCTCGATCATGATGTGCCGATCATCGACACCCGCCATGAGGCCGCCGCAGGGCAT

A  A  E  G  Y  A  R  A  G  A  K  L  G  V  A  L  V  T  A  G  G  G  F  T  N
GCGGCCGAGGGCTATGCCCGCGCTGGCGCCAAGCTGGGCGTGGCGCTGGTCACGGCGGGCGGGGGATTTACCAAT

A  V  T  P  I  A  N  A  W  L  D  R  T  P  V  L  F  L  T  G  S  G  A  L  R
GCGGTCACGCCCATTGCCAACGCTTGGCTGGATCGCACGCCGGTGCTCTTCCTCACCGGATCGGGCGCGCTGCGT

D  D  E  T  N  T  L  Q  A  G  I  D  Q  V  A  M  A  A  P  I  T  K  W  A  H
GATGATGAAACCAACACGTTGCAGGCGGGGATTGATCAGGTCGCCATGGCGGCGCCCATTACCAAATGGGCGCAT

R  V  M  A  T  E  H  I  P  R  L  V  M  Q  A  I  R  A  A  L  S  A  P  R  G
CGGGTGATGGCAACCGAGCATATCCCACGGCTGGTGATGCAGGCGATCCGCGCCGCGTTGAGCGCGCCACGCGGG

P  V  L  L  D  L  P  W  D  I  L  M  N  Q  I  D  E  D  S  V  I  I  P  D  L
CCGGTGTTGCTGGATCTGCCGTGGGATATTCTGATGAACCAGATTGATGAGGATAGCGTCATTATCCCCGATCTG

V  L  S  A  H  G  A  R  P  D  P  A  D  L  D  Q  A  L  A  L  L  R  K  A  E
GTCTTGTCCGCACATGGGGCCAGACCCGACCCTGCCGATCTGGATCAGGCTCTCGCGCTTTTGCGCAAGGCGGAG

R  P  V  I  V  L  G  S  E  A  S  R  T  A  R  K  T  A  L  S  A  F  V  A  A
CGGCCGGTCATCGTGCTCGGCTCAGAAGCCTCGCGGACAGCGCGCAAGACGGCGCTTAGCGCATTCGTGGCGGCG

T  G  V  P  V  F  A  D  Y  E  G  L  S  M  L  S  G  L  P  D  A  M  R  G  G
ACTGGCGTGCCGGTGTTTGCCGATTATGAAGGGCTAAGCATGCTCTCGGGGCTGCCCGATGCTATGCGGGGCGGG

L  V  Q  N  L  Y  S  F  A  K  A  D  A  A  P  D  L  V  L  M  L  G  A  R  F
CTGGTGCAAAACCTCTATTCTTTTGCCAAAGCCGATGCCGCGCCAGATCTCGTGCTGATGCTGGGGGCGCGCTTT

G  L  N  T  G  H  G  S  G  Q  L  I  P  H  S  A  Q  V  I  Q  V  D  P  D  A
GGCCTTAACACCGGGCATGGATCTGGGCAGTTGATCCCCCATAGCGCGCAGGTCATTCAGGTCGACCCTGATGCC

C  E  L  G  R  L  Q  G  I  A  L  G  I  V  A  D  V  G  G  T  I  E  A  L  A
TGCGAGCTGGGACGCCTGCAGGGCATCGCTCTGGGCATTGTGGCCGATGTGGGTGGGACCATCGAGGCTTTGGCG

Q  A  T  A  Q  D  A  A  W  P  D  R  G  D  W  C  A  K  V  T  D  L  A  Q  E
CAGGCCACCGCGCAAGATGCGGCTTGGCCGGATCGCGGCGACTGGTGCGCCAAAGTGACGGATCTGGCGCAAGAG

R  Y  A  S  I  A  A  K  S  S  S  E  H  A  L  H  P  F  H  A  S  Q  V  I  A
CGCTATGCCAGCATCGCTGCGAAATCGAGCAGCGAGCATGCGCTCCACCCCTTTCACGCCTCGCAGGTCATTGCC

K  H  V  D  A  G  V  T  V  V  A  D  G  A  L  T  Y  L  W  L  S  E  V  M  S
AAACACGTCGATGCAGGGGTGACGGTGGTAGCGGATGGTGCGCTGACCTATCTCTGGCTGTCCGAAGTGATGAGC

R  V  K  P  G  G  F  L  C  H  G  Y  L  G  S  M  G  V  G  F  G  T  A  L  G
CGCGTGAAACCCGGCGGTTTTCTCTGCCACGGCTATCTAGGCTCGATGGGCGTGGGCTTCGGCACGGCGCTGGGC

A  Q  V  A  D  L  E  A  G  R  R  T  I  L  V  T  G  D  G  S  V  G  Y  S  I
GCGCAAGTGGCCGATCTTGAAGCAGGCCGCCGCACGATCCTTGTGACCGGCGATGGCTCGGTGGGCTATAGCATC
```

G E F D T L V R K Q L P L I V I I M N N Q S W G A
GGTGAATTTGATACGCTGGTGCGCAAACAATTGCCGCTGATCGTCATCATCATGAACAACCAAAGCTGGGGGGCG

T L H F Q Q L A V G P N R V T G T R L E N G S Y H
ACATTGCATTTCCAGCAATTGGCCGTCGGCCCCAATCGCGTGACGGGCACCCGTTTGGAAAATGGCTCCTATCAC

G V A A A F G A D G Y H V D S V E S F S A A L A Q
GGGGTGGCCGCCGCCTTTGGCGCGGATGGCTATCATGTCGACAGTGTGGAGAGCTTTTCTGCGGCTCTGGCCCAA

A L A H N R P A C I N V A V A L D P I P P E E L I
GCGCTCGCCCATAATCGCCCCGCCTGCATCAATGTCGCGGTCGCGCTCGATCCGATCCCGCCCGAAGAACTCATT

L I G M D P F A *
CTGATCGGCATGGACCCCTTCGCATAAGCGGCCGCACTCGAG
*Not*I *Xho*I

***Pp*BFD *L476Q* –His$_6$**

ATGGCTTCGGTACACGGCACCACATACGAACTCTTGCGACGTCAAGGCATCGATACGGTCTTCGGCAATCCTGGC
M A S V H G T T Y E L L R R Q G I D T V F G N P G

TCGAACGAGCTCCCGTTTTTGAAGGACTTTCCAGAGGACTTTCGATACATCCTGGCTTTGCAGGAAGCGTGTGTG
S N E L P F L K D F P E D F R Y I L A L Q E A C V

GTGGGCATTGCAGACGGCTATGCGCAAGCCAGTCGGAAGCCGGCTTTCATTAACCTGCATTCTGCTGCTGGTACC
V G I A D G Y A Q A S R K P A F I N L H S A A G T

GGCAATGCTATGGGTGCACTCAGTAACGCCTGGAACTCACATTCCCCGCTGATCGTCACTGCCGGCCAGCAGACC
G N A M G A L S N A W N S H S P L I V T A G Q Q T

AGGGCGATGATTGGCGTTGAAGCTCTGCTGACCAACGTCGATGCCGCCAACCTGCCACGACCACTTGTCAAATGG
R A M I G V E A L L T N V D A A N L P R P L V K W

AGCTACGAGCCCGCAAGCGCAGCAGAAGTCCCTCATGCGATGAGCAGGGCTATCCATATGGCAAGCATGGCGCCA
S Y E P A S A A E V P H A M S R A I H M A S M A P

CAAGGCCCTGTCTATCTTTCGGTGCCATATGACGATTGGGATAAGGATGCTGATCCTCAGTCCCACCACCTTTTT
Q G P V Y L S V P Y D D W D K D A D P Q S H H L F

GATCGCCATGTCAGTTCATCAGTACGCCTGAACGACCAGGATCTCGATATTCTGGTGAAAGCTCTCAACAGCGCA
D R H V S S S V R L N D Q D L D I L V K A L N S A

TCCAACCCGGCGATCGTCCTGGGCCCGGACGTCGACGCAGCAAATGCGAACGCAGACTGCGTCATGTTGGCCGAA
S N P A I V L G P D V D A A N A N A D C V M L A E

CGCCTCAAAGCTCCGGTTTGGGTTGCGCCATCCGCTCCACGCTGCCCATTCCCTACCCGTCATCCTTGCTTCCGT
R L K A P V W V A P S A P R C P F P T R H P C F R

GGATTGATGCCAGCTGGCATCGCAGCGATTTCTCAGCTGCTCGAAGGTCACGATGTGGTTTTGGTAATCGGCGCT
G L M P A G I A A I S Q L L E G H D V V L V I G A

CCAGTGTTCCGTTACCACCAATACGACCCAGGTCAATATCTCAAACCTGGCACGCGATTGATTTCGGTGACCTGC
P V F R Y H Q Y D P G Q Y L K P G T R L I S V T C

GACCCGCTCGAAGCTGCACGCGCGCCAATGGGCGATGCGATCGTGGCAGACATTGGTGCGATGGCTAGCGCTCTT
D P L E A A R A P M G D A I V A D I G A M A S A L

GCCAACTTGGTTGAAGAGAGCAGCCGCCAGCTCCCAACTGCAGCTCCGGAACCCGCGAAGGTTGACCAAGACGCT
A N L V E E S S R Q L P T A A P E P A K V D Q D A

GGCCGACTTCACCCAGAGACAGTGTTCGACACACTGAACGACATGGCCCCGGAGAATGCGATTTACCTGAACGAG

G R L H P E T V F D T L N D M A P E N A I Y L N E

TCGACTTCAACGACCGCCCAAATGTGGCAGCGCCTGAACATGCGCAACCCTGGTAGCTACTACTTCTGTGCAGCT
S T S T T A Q M W Q R L N M R N P G S Y Y F C A A

GGCGGACTGGGCTTCGCCCTGCCTGCAGCAATTGGCGTTCAACTCGCAGAACCCGAGCGACAAGTCATCGCCGTC
G G L G F A L P A A I G V Q L A E P E R Q V I A V

ATTGGCGACGGATCGGCGAACTACAGCATTAGTGCGTTGTGGACTGCAGCTCAGTACAACATCCCCACTATCTTC
I G D G S A N Y S I S A L W T A A Q Y N I P T I F

GTGATCATGAACAACGGCACCTACGGTGCGTTGCGATGGTTTGCCGGCGTTCTCGAAGCAGAAAACGTTCCTGGG
V I M N N G T Y G A L R W F A G V L E A E N V P G

CAGGATGTGCCAGGGATCGACTTCCGCGCACTCGCCAAGGGCTATGGGGTCCAAGCGCTGAAAGCCGACAACCTT
Q D V P G I D F R A L A K G Y G V Q A L K A D N L

GAGCAGCTCAAGGGTTCGCTACAAGAAGCGCTTTCTGCCAAAGGCCCGGTACTTATCGAAGTAAGCACCGTAAGC
E Q L K G S L Q E A L S A K G P V L I E V S T V S

CCGGTGAAGAGATCTCATCACCATCACCATCACTAAGCTTCTAGAGGATCC
P V K R S H H H H H H * A S R G S
*Hind*III

TDoT-L-*Pp*BFD *L476Q*

CATATGATCATTAACGAAACTGCCGATGACATCGTTTATCGCCTGACAGTCATTATCGATGATCGCTACGAATCG
M I I N E T A D D I V Y R L T V I I D D R Y E S
***Nde*I** TDoT
CTGAAAAACCTGATTACCTTACGTGCAGATCGCTTGGAGATGATCATCAATGACAATGTGTCCACCATTCTCGCG
L K N L I T L R A D R L E M I I N D N V S T I L A

AGCATTACTAGTATTGAAGGCCGTGCTAGCGGCGGTGGGTCTGGAGGCGGCTCAGGTGGTGGGTCGGGATCCATG
S I T S I E G R A S G G G S G G G S G G G S G S M
***Spe*I** **Xa** ***Nhe*I** **3xGGGS-Linker** ***Bam*HI**
GCTTCGGTACACGGCACCACATACGAACTCTTGCGACGTCAAGGCATCGATACGGTCTTCGGCAATCCTGGCTCG
A S V H G T T Y E L L R R Q G I D T V F G N P G S
***Pp*BFD L476Q**
AACGAGCTCCCGTTTTTGAAGGACTTTCCAGAGGACTTTCGATACATCCTGGCTTTGCAGGAAGCGTGTGTGGTG
N E L P F L K D F P E D F R Y I L A L Q E A C V V

GGCATTGCAGACGGCTATGCGCAAGCCAGTCGGAAGCCGGCTTTCATTAACCTGCATTCTGCTGCTGGTACCGGC
G I A D G Y A Q A S R K P A F I N L H S A A G T G

AATGCTATGGGTGCACTCAGTAACGCCTGGAACTCACATTCCCCGCTGATCGTCACTGCCGGCCAGCAGACCAGG
N A M G A L S N A W N S H S P L I V T A G Q Q T R

GCGATGATTGGCGTTGAAGCTCTGCTGACCAACGTCGATGCCGCCAACCTGCCACGACCACTTGTCAAATGGAGC
A M I G V E A L L T N V D A A N L P R P L V K W S

TACGAGCCCGCAAGCGCAGCAGAAGTCCCTCATGCGATGAGCAGGGCTATCCATATGGCAAGCATGGCGCCACAA
Y E P A S A A E V P H A M S R A I H M A S M A P Q

GGCCCTGTCTATCTTTCGGTGCCATATGACGATTGGGATAAGGATGCTGATCCTCAGTCCCACCACCTTTTTGAT
G P V Y L S V P Y D D W D K D A D P Q S H H L F D

CGCCATGTCAGTTCATCAGTACGCCTGAACGACCAGGATCTCGATATTCTGGTGAAAGCTCTCAACAGCGCATCC
R H V S S S V R L N D Q D L D I L V K A L N S A S

AACCCGGCGATCGTCCTGGGCCCGGACGTCGACGCAGCAAATGCGAACGCAGACTGCGTCATGTTGGCCGAACGC

N P A I V L G P D V D A A N A N A D C V M L A E R

CTCAAAGCTCCGGTTTGGGTTGCGCCATCCGCTCCACGCTGCCCATTCCCTACCCGTCATCCTTGCTTCCGTGGA
L K A P V W V A P S A P R C P F P T R H P C F R G

TTGATGCCAGCTGGCATCGCAGCGATTTCTCAGCTGCTCGAAGGTCACGATGTGGTTTTGGTAATCGGCGCTCCA
L M P A G I A A I S Q L L E G H D V V L V I G A P

GTGTTCCGTTACCACCAATACGACCCAGGTCAATATCTCAAACCTGGCACGCGATTGATTTCGGTGACCTGCGAC
V F R Y H Q Y D P G Q Y L K P G T R L I S V T C D

CCGCTCGAAGCTGCACGCGCGCCAATGGGCGATGCGATCGTGGCAGACATTGGTGCGATGGCTAGCGCTCTTGCC
P L E A A R A P M G D A I V A D I G A M A S A L A

AACTTGGTTGAAGAGAGCAGCCGCCAGCTCCCAACTGCAGCTCCGGAACCCGCGAAGGTTGACCAAGACGCTGGC
N L V E E S S R Q L P T A A P E P A K V D Q D A G

CGACTTCACCCAGAGACAGTGTTCGACACACTGAACGACATGGCCCCGGAGAATGCGATTTACCTGAACGAGTCG
R L H P E T V F D T L N D M A P E N A I Y L N E S

ACTTCAACGACCGCCCAAATGTGGCAGCGCCTGAACATGCGCAACCCTGGTAGCTACTACTTCTGTGCAGCTGGC
T S T T A Q M W Q R L N M R N P G S Y Y F C A A G

GGACTGGGCTTCGCCCTGCCTGCAGCAATTGGCGTTCAACTCGCAGAACCCGAGCGACAAGTCATCGCCGTCATT
G L G F A L P A A I G V Q L A E P E R Q V I A V I

GGCGACGGATCGGCGAACTACAGCATTAGTGCGTTGTGGACTGCAGCTCAGTACAACATCCCCACTATCTTCGTG
G D G S A N Y S I S A L W T A A Q Y N I P T I F V

ATCATGAACAACGGCACCTACGGTGCGTTGCGATGGTTTGCCGGCGTTCTCGAAGCAGAAAACGTTCCTGGGCAG
I M N N G T Y G A L R W F A G V L E A E N V P G Q

GATGTGCCAGGGATCGACTTCCGCGCACTCGCCAAGGGCTATGGGGTCCAAGCGCTGAAAGCCGACAACCTTGAG
D V P G I D F R A L A K G Y G V Q A L K A D N L E

CAGCTCAAGGGTTCGCTACAAGAAGCGCTTTCTGCCAAAGGCCCGGTACTTATCGAAGTAAGCACCGTAAGCCCG
Q L K G S L Q E A L S A K G P V L I E V S T V S P

GTGAAGAGATCTTAAGCGGCCGCACTCGAG
V K R S * **A A A L E**
***Not*I**

3HAMP-L-*Pp*BFD *L476Q*

CATATGGGCCTGTTTAACGCCCATGCAGTTGCGCAGCAACGCGCGGATCGCATTGCGACTCTCCTGCAGTCCTTT
M G L F N A H A V A Q Q R A D R I A T L L Q S F
***Nde*I** 3HAMP

GCGGATGGTCAGTTGGACACCGCCGTGGGTGAAGCGCCAGCACCTGGTTACGAACGCCTGTATGACTCGCTTCGC
A D G Q L D T A V G E A P A P G Y E R L Y D S L R

GCCCTTCAGCGCCAACTGCGCGAACAACGTGCGGAGTTACAACAGGTTGAGAGCCTGGAAGCAGGCTTGGCTGAA
A L Q R Q L R E Q R A E L Q Q V E S L E A G L A E

ATGAGTCGGCAGCATGAAGCAGGGTGGATTGACCAGACGATTCCGGCTGAACGGTTAGAGGGCCGTGCAGCACGT
M S R Q H E A G W I D Q T I P A E R L E G R A A R

ATCGCCAAAGGCGTGAATGAGCTGGTTGCTGCGCACATTGCGGTGAAAATGAAAGTCGTGAGCGTAGTCACCGCG
I A K G V N E L V A A H I A V K M K V V S V V T A

TATGGCCAAGGGAACTTCGAACCGCTCATGGATCGCCTGCCGGGTAAGAAAGCCCAGATCACGGAGGCCATTGAT
Y G Q G N F E P L M D R L P G K K A Q I T E A I D

GGCGTACGTGAACGCCTGCGTGGAGCTGCTGAAGCGACCTCTGCGCAGCTGGCCACAGCCGCCTACAATACTAGT

G V R E R L R G A A E A T S A Q L A T A A Y N T S

*Spe*I

ATTGAAGGCCGTGCTAGCGGCGGTGGGTCTGGAGGCGGCTCAGGTGGTGGGTCGGGATCCATGGCTTCGGTACAC

I E G R A S G G G S G G G S G G G S G S M A S V H

Xa *Nhe*I 3xGGGS-Linker *Bam*HI *Pp*BFD

L476Q

GGCACCACATACGAACTCTTGCGACGTCAAGGCATCGATACGGTCTTCGGCAATCCTGGCTCGAACGAGCTCCCG

G T T Y E L L R R Q G I D T V F G N P G S N E L P

TTTTTGAAGGACTTTCCAGAGGACTTTCGATACATCCTGGCTTTGCAGGAAGCGTGTGTGGTGGGCATTGCAGAC

F L K D F P E D F R Y I L A L Q E A C V V G I A D

GGCTATGCGCAAGCCAGTCGGAAGCCGGCTTTCATTAACCTGCATTCTGCTGCTGGTACCGGCAATGCTATGGGT

G Y A Q A S R K P A F I N L H S A A G T G N A M G

GCACTCAGTAACGCCTGGAACTCACATTCCCCGCTGATCGTCACTGCCGGCCAGCAGACCAGGGCGATGATTGGC

A L S N A W N S H S P L I V T A G Q Q T R A M I G

GTTGAAGCTCTGCTGACCAACGTCGATGCCGCCAACCTGCCACGACCACTTGTCAAATGGAGCTACGAGCCCGCA

V E A L L T N V D A A N L P R P L V K W S Y E P A

AGCGCAGCAGAAGTCCCTCATGCGATGAGCAGGGCTATCCATATGGCAAGCATGGCGCCACAAGGCCCTGTCTAT

S A A E V P H A M S R A I H M A S M A P Q G P V Y

CTTTCGGTGCCATATGACGATTGGGATAAGGATGCTGATCCTCAGTCCCACCACCTTTTTGATCGCCATGTCAGT

L S V P Y D D W D K D A D P Q S H H L F D R H V S

TCATCAGTACGCCTGAACGACCAGGATCTCGATATTCTGGTGAAAGCTCTCAACAGCGCATCCAACCCGGCGATC

S S V R L N D Q D L D I L V K A L N S A S N P A I

GTCCTGGGCCCGGACGTCGACGCAGCAAATGCGAACGCAGACTGCGTCATGTTGGCCGAACGCCTCAAAGCTCCG

V L G P D V D A A N A N A D C V M L A E R L K A P

GTTTGGGTTGCGCCATCCGCTCCACGCTGCCCATTCCCTACCCGTCATCCTTGCTTCCGTGGATTGATGCCAGCT

V W V A P S A P R C P F P T R H P C F R G L M P A

GGCATCGCAGCGATTTCTCAGCTGCTCGAAGGTCACGATGTGGTTTTGGTAATCGGCGCTCCAGTGTTCCGTTAC

G I A A I S Q L L E G H D V V L V I G A P V F R Y

CACCAATACGACCCAGGTCAATATCTCAAACCTGGCACGCGATTGATTTCGGTGACCTGCGACCCGCTCGAAGCT

H Q Y D P G Q Y L K P G T R L I S V T C D P L E A

GCACGCGCGCCAATGGGCGATGCGATCGTGGCAGACATTGGTGCGATGGCTAGCGCTCTTGCCAACTTGGTTGAA

A R A P M G D A I V A D I G A M A S A L A N L V E

GAGAGCAGCCGCCAGCTCCCAACTGCAGCTCCGGAACCCGCGAAGGTTGACCAAGACGCTGGCCGACTTCACCCA

E S S R Q L P T A A P E P A K V D Q D A G R L H P

GAGACAGTGTTCGACACACTGAACGACATGGCCCCGGAGAATGCGATTTACCTGAACGAGTCGACTTCAACGACC

E T V F D T L N D M A P E N A I Y L N E S T S T T

GCCCAAATGTGGCAGCGCCTGAACATGCGCAACCCTGGTAGCTACTACTTCTGTGCAGCTGGCGGACTGGGCTTC

A Q M W Q R L N M R N P G S Y Y F C A A G G L G F

GCCCTGCCTGCAGCAATTGGCGTTCAACTCGCAGAACCCGAGCGACAAGTCATCGCCGTCATTGGCGACGGATCG

A L P A A I G V Q L A E P E R Q V I A V I G D G S

GCGAACTACAGCATTAGTGCGTTGTGGACTGCAGCTCAGTACAACATCCCCACTATCTTCGTGATCATGAACAAC

A N Y S I S A L W T A A Q Y N I P T I F V I M N N

GGCACCTACGGTGCGTTGCGATGGTTTGCCGGCGTTCTCGAAGCAGAAAACGTTCCTGGGCAGGATGTGCCAGGG

```
 G  T  Y  G  A  L  R  W  F  A  G  V  L  E  A  E  N  V  P  G  Q  D  V  P  G

ATCGACTTCCGCGCACTCGCCAAGGGCTATGGGGTCCAAGCGCTGAAAGCCGACAACCTTGAGCAGCTCAAGGGT
 I  D  F  R  A  L  A  K  G  Y  G  V  Q  A  L  K  A  D  N  L  E  Q  L  K  G

TCGCTACAAGAAGCGCTTTCTGCCAAAGGCCCGGTACTTATCGAAGTAAGCACCGTAAGCCCGGTGAAGAGATCT
 S  L  Q  E  A  L  S  A  K  G  P  V  L  I  E  V  S  T  V  S  P  V  K  R  S

TAAGCGGCCGCACTCGAG
 *  A  A  A  L  E
     NotI
```

TDoT-L-*Ec*LDC

```
CATATGATCATTAACGAAACTGCCGATGACATCGTTTATCGCCTGACAGTCATTATCGATGATCGCTACGAATCG
    M  I  I  N  E  T  A  D  D  I  V  Y  R  L  T  V  I  I  D  D  R  Y  E  S
NdeI                                                                     TDoT
CTGAAAAACCTGATTACCTTACGTGCAGATCGCTTGGAGATGATCATCAATGACAATGTGTCCACCATTCTCGCG
 L  K  N  L  I  T  L  R  A  D  R  L  E  M  I  I  N  D  N  V  S  T  I  L  A

AGCATTACTAGTATTGAAGGCCGTGCTAGCGGCGGTGGGTCTGGAGGCGGCTCAGGTGGTGGGTCGGGATCCATG
 S  I  T  S  I  E  G  R  A  S  G  G  G  S  G  G  G  S  G  G  G  S  G  S  M
       SpeI        Xa        NheI        3xGGGS-Linker                BamHI
AACATCATCGCTATCATGGGCCCTCACGGTGTTTTCTACAAGGATGAGCCAATCAAGGAGCTGGAATCCGCACTA
 N  I  I  A  I  M  G  P  H  G  V  F  Y  K  D  E  P  I  K  E  L  E  S  A  L
 EcLDC
GTTGCACAGGGCTTTCAGATCATCTGGCCCCAGAACTCCGTTGACCTTCTCAAATTCATCGAGCACAATCCTCGC
 V  A  Q  G  F  Q  I  I  W  P  Q  N  S  V  D  L  L  K  F  I  E  H  N  P  R

ATTTGTGGTGTGATTTTTGACTGGGACGAGTACTCTCTTGATTTATGCTCCGACATCAACCAGCTCAACGAGTAC
 I  C  G  V  I  F  D  W  D  E  Y  S  L  D  L  C  S  D  I  N  Q  L  N  E  Y

CTGCCACTCTACGCATTCATCAACACTCACTCCACCATGGACGTTTCCGTGCAGGACATGCGTATGGCACTCTGG
 L  P  L  Y  A  F  I  N  T  H  S  T  M  D  V  S  V  Q  D  M  R  M  A  L  W

TTCTTTGAATACGCTCTGGGCCAGGCTGAGGACATCGCGATCCGCATGCGTCAGTACACCGACGAGTACCTGGAC
 F  F  E  Y  A  L  G  Q  A  E  D  I  A  I  R  M  R  Q  Y  T  D  E  Y  L  D

AACATCACCCCTCCATTCACCAAGGCTCTCTTCACCTACGTAAAGGAACGCAAGTACACTTTCTGCACCCCAGGC
 N  I  T  P  P  F  T  K  A  L  F  T  Y  V  K  E  R  K  Y  T  F  C  T  P  G

CACATGGGCGGCACCGCCTACCAGAAGTCCCCAGTCGGATGCCTCTTCTACGACTTCTTCGGCGGTAACACTCTT
 H  M  G  G  T  A  Y  Q  K  S  P  V  G  C  L  F  Y  D  F  F  G  G  N  T  L

AAGGCAGATGTCTCCATTTCCGTCACCGAGTTGGGCTCTCTGCTGGACCACACCGGCCCTCACCTGGAGGCAGAA
 K  A  D  V  S  I  S  V  T  E  L  G  S  L  L  D  H  T  G  P  H  L  E  A  E

GAGTACATCGCTCGTACCTTCGGTGCTGAACAGTCCTACATCGTCACCAACGGTACTTCCACCAGCAACAAGATC
 E  Y  I  A  R  T  F  G  A  E  Q  S  Y  I  V  T  N  G  T  S  T  S  N  K  I

GTTGGTATGTACGCAGCTCCTTCTGGCTCCACCCTGTTGATCGACCGCAACTGTCACAAGTCCCTCGCGCATCTT
 V  G  M  Y  A  A  P  S  G  S  T  L  L  I  D  R  N  C  H  K  S  L  A  H  L

CTTATGATGAACGATGTGGTCCCTGTATGGCTGAAGCCAACCCGTAACGCTCTGGGCATCCTTGGCGGTATCCCC
 L  M  M  N  D  V  V  P  V  W  L  K  P  T  R  N  A  L  G  I  L  G  G  I  P

CGTCGCGAGTTCACCCGTGATTCCATCGAGGAAAAGGTTGCAGCCACTACCCAGGCACAGTGGCCTGTCCACGCT
 R  R  E  F  T  R  D  S  I  E  E  K  V  A  A  T  T  Q  A  Q  W  P  V  H  A

GTCATTACCAACTCGACCTACGACGGCCTGCTCTACAACACCGATTGGATCAAGCAGACCCTAGATGTTCCTTCC
```

V I T N S T Y D G L L Y N T D W I K Q T L D V P S

ATTCACTTCGACAGCGCATGGGTTCCTTACACTCACTTCCACCCAATCTACCAGGGTAAGTCCGGAATGTCCGGC
I H F D S A W V P Y T H F H P I Y Q G K S G M S G

GAGCGTGTCGCTGGCAAGGTTATCTTCGAAACCCAATCAACCCACAAGATGCTGGCTGCTCTCTCCCAGGCTTCT
E R V A G K V I F E T Q S T H K M L A A L S Q A S

CTGATCCACATCAAGGGCGAGTACGACGAGGAAGCTTTCAACGAGGCTTTCATGATGCACACCACCACCTCCCCA
L I H I K G E Y D E E A F N E A F M M H T T T S P

TCCTACCCTATCGTCGCGTCCGTCGAGACTGCTGCCGCAATGCTTCGCGGTAACCCAGGTAAGCGCCTCATCAAC
S Y P I V A S V E T A A A M L R G N P G K R L I N

CGTTCCGTTGAGCGCGCTCTTCACTTCCGTAAGGAAGTGCAGCGCCTGCGTGAGGAATCTGACGGTTGGTTCTTC
R S V E R A L H F R K E V Q R L R E E S D G W F F

GACATTTGGCAGCCACCTCAGGTTGATGAGGCCGAGTGCTGGCCAGTTGCTCCAGGTGAACAGTGGCACGGATTC
D I W Q P P Q V D E A E C W P V A P G E Q W H G F

AACGATGCAGATGCTGACCACATGTTTTTGGACCCGGTCAAGGTCACCATTCTTACTCCTGGTATGGATGAGCAG
N D A D A D H M F L D P V K V T I L T P G M D E Q

GGCAACATGTCTGAGGAGGGTATCCCAGCTGCTCTGGTTGCAAAGTTCCTCGACGAACGTGGCATCGTTGTTGAG
G N M S E E G I P A A L V A K F L D E R G I V V E

AAGACCGGACCATACAACCTGCTGTTCCTGTTCAGCATCGGCATCGACAAAACCAAGGCAATGGGTCTGCTGCGC
K T G P Y N L L F L F S I G I D K T K A M G L L R

GGCCTTACCGAGTTCAAGCGCTCCTACGACCTGAACCTTCGCATCAAGAATATGCTGCCGGACCTGTACGCTGAA
G L T E F K R S Y D L N L R I K N M L P D L Y A E

GATCCTGATTTCTACCGCAACATGCGCATCCAGGACCTCGCACAGGGCATCCACAAGCTCATTCGCAAGCACGAC
D P D F Y R N M R I Q D L A Q G I H K L I R K H D

CTGCCAGGCCTTATGCTCCGTGCATTCGATACCCTCCCAGAGATGATCATGACCCCTCACCAGGCTTGGCAGCGC
L P G L M L R A F D T L P E M I M T P H Q A W Q R

CAGATCAAGGGCGAGGTGGAAACCATCGCACTGGAGCAGCTGGTTGGTCGTGTCTCCGCCAACATGATCCTGCCA
Q I K G E V E T I A L E Q L V G R V S A N M I L P

TATCCACCTGGCGTTCCGCTGCTGATGCCAGGCGAGATGCTCACCAAGGAGTCCCGCACCGTATTGGACTTCCTT
Y P P G V P L L M P G E M L T K E S R T V L D F L

CTCATGTTGTGCTCTGTTGGCCAGCACTACCCAGGCTTCGAGACCGACATCCACGGCGCTAAGCAAGATGAAGAC
L M L C S V G Q H Y P G F E T D I H G A K Q D E D

GGCGTTTACCGCGTTCGCGTCCTTAAGATGGCAGGCTAAGTCGACAAGCTTGCGGCCGCACTCGAG
G V Y R V R V L K M A G * **V D K L A A A L E**
*Sal*I *Not*I

*Ec*LDC-L-TDoT

CATATGATGAACATCATCGCTATCATGGGCCCTCACGGTGTTTTCTACAAGGATGAGCCAATCAAGGAGCTGGAA
H M M N I I A I M G P H G V F Y K D E P I K E L E
*Nde*I EcLDC

TCCGCACTAGTTGCACAGGGCTTTCAGATCATCTGGCCCCAGAACTCCGTTGACCTTCTCAAATTCATCGAGCAC
S A L V A Q G F Q I I W P Q N S V D L L K F I E H

AATCCTCGCATTTGTGGTGTGATTTTTGACTGGGACGAGTACTCTCTTGATTTATGCTCCGACATCAACCAGCTC

N P R I C G V I F D W D E Y S L D L C S D I N Q L

AACGAGTACCTGCCACTCTACGCATTCATCAACACTCACTCCACCATGGACGTTTCCGTGCAGGACATGCGTATG
N E Y L P L Y A F I N T H S T M D V S V Q D M R M

GCACTCTGGTTCTTTGAATACGCTCTGGGCCAGGCTGAGGACATCGCGATCCGCATGCGTCAGTACACCGACGAG
A L W F F E Y A L G Q A E D I A I R M R Q Y T D E

TACCTGGACAACATCACCCCTCCATTCACCAAGGCTCTCTTCACCTACGTAAAGGAACGCAAGTACACTTTCTGC
Y L D N I T P P F T K A L F T Y V K E R K Y T F C

ACCCCAGGCCACATGGGCGGCACCGCCTACCAGAAGTCCCCAGTCGGATGCCTCTTCTACGACTTCTTCGGCGGT
T P G H M G G T A Y Q K S P V G C L F Y D F F G G

AACACTCTTAAGGCAGATGTCTCCATTTCCGTCACCGAGTTGGGCTCTCTGCTGGACCACACCGGCCCTCACCTG
N T L K A D V S I S V T E L G S L L D H T G P H L

GAGGCAGAAGAGTACATCGCTCGTACCTTCGGTGCTGAACAGTCCTACATCGTCACCAACGGTACTTCCACCAGC
E A E E Y I A R T F G A E Q S Y I V T N G T S T S

AACAAGATCGTTGGTATGTACGCAGCTCCTTCTGGCTCCACCCTGTTGATCGACCGCAACTGTCACAAGTCCCTC
N K I V G M Y A A P S G S T L L I D R N C H K S L

GCGCATCTTCTTATGATGAACGATGTGGTCCCTGTATGGCTGAAGCCAACCCGTAACGCTCTGGGCATCCTTGGC
A H L L M M N D V V P V W L K P T R N A L G I L G

GGTATCCCCCGTCGCGAGTTCACCCGTGATTCCATCGAGGAAAAGGTTGCAGCCACTACCCAGGCACAGTGGCCT
G I P R R E F T R D S I E E K V A A T T Q A Q W P

GTCCACGCTGTCATTACCAACTCGACCTACGACGGCCTGCTCTACAACACCGATTGGATCAAGCAGACCCTAGAT
V H A V I T N S T Y D G L L Y N T D W I K Q T L D

GTTCCTTCCATTCACTTCGACAGCGCATGGGTTCCTTACACTCACTTCCACCCAATCTACCAGGGTAAGTCCGGA
V P S I H F D S A W V P Y T H F H P I Y Q G K S G

ATGTCCGGCGAGCGTGTCGCTGGCAAGGTTATCTTCGAAACCCAATCAACCCACAAGATGCTGGCTGCTCTCTCC
M S G E R V A G K V I F E T Q S T H K M L A A L S

CAGGCTTCTCTGATCCACATCAAGGGCGAGTACGACGAGGAAGCTTTCAACGAGGCTTTCATGATGCACACCACC
Q A S L I H I K G E Y D E E A F N E A F M M H T T

ACCTCCCCATCCTACCCTATCGTCGCGTCCGTCGAGACTGCTGCCGCAATGCTTCGCGGTAACCCAGGTAAGCGC
T S P S Y P I V A S V E T A A A M L R G N P G K R

CTCATCAACCGTTCCGTTGAGCGCGCTCTTCACTTCCGTAAGGAAGTGCAGCGCCTGCGTGAGGAATCTGACGGT
L I N R S V E R A L H F R K E V Q R L R E E S D G

TGGTTCTTCGACATTTGGCAGCCACCTCAGGTTGATGAGGCCGAGTGCTGGCCAGTTGCTCCAGGTGAACAGTGG
W F F D I W Q P P Q V D E A E C W P V A P G E Q W

CACGGATTCAACGATGCAGATGCTGACCACATGTTTTTGGACCCGGTCAAGGTCACCATTCTTACTCCTGGTATG
H G F N D A D A D H M F L D P V K V T I L T P G M

GATGAGCAGGGCAACATGTCTGAGGAGGGTATCCCAGCTGCTCTGGTTGCAAAGTTCCTCGACGAACGTGGCATC
D E Q G N M S E E G I P A A L V A K F L D E R G I

GTTGTTGAGAAGACCGGACCATACAACCTGCTGTTCCTGTTCAGCATCGGCATCGACAAAACCAAGGCAATGGGT
V V E K T G P Y N L L F L F S I G I D K T K A M G

CTGCTGCGCGGCCTTACCGAGTTCAAGCGCTCCTACGACCTGAACCTTCGCATCAAGAATATGCTGCCGGACCTG
L L R G L T E F K R S Y D L N L R I K N M L P D L

TACGCTGAAGATCCTGATTTCTACCGCAACATGCGCATCCAGGACCTCGCACAGGGCATCCACAAGCTCATTCGC

```
 Y  A  E  D  P  D  F  Y  R  N  M  R  I  Q  D  L  A  Q  G  I  H  K  L  I  R

AAGCACGACCTGCCAGGCCTTATGCTCCGTGCATTCGATACCCTCCCAGAGATGATCATGACCCCTCACCAGGCT
 K  H  D  L  P  G  L  M  L  R  A  F  D  T  L  P  E  M  I  M  T  P  H  Q  A

TGGCAGCGCCAGATCAAGGGCGAGGTGGAAACCATCGCACTGGAGCAGCTGGTTGGTCGTGTCTCCGCCAACATG
 W  Q  R  Q  I  K  G  E  V  E  T  I  A  L  E  Q  L  V  G  R  V  S  A  N  M

ATCCTGCCATATCCACCTGGCGTTCCGCTGCTGATGCCAGGCGAGATGCTCACCAAGGAGTCCCGCACCGTATTG
 I  L  P  Y  P  P  G  V  P  L  L  M  P  G  E  M  L  T  K  E  S  R  T  V  L

GACTTCCTTCTCATGTTGTGCTCTGTTGGCCAGCACTACCCAGGCTTCGAGACCGACATCCACGGCGCTAAGCAA
 D  F  L  L  M  L  C  S  V  G  Q  H  Y  P  G  F  E  T  D  I  H  G  A  K  Q

GATGAAGACGGCGTTTACCGCGTTCGCGTCCTTAAGATGGCAGGCGCTAGCGGCGGTGGGTCTGGAGGCGGCTCA
 D  E  D  G  V  Y  R  V  R  V  L  K  M  A  G  A  S  G  G  G  S  G  G  G  S
                                                NheI             3xGGGS-Linker
GGTGGTGGGTCGGGATCCATCATTAACGAAACTGCCGATGACATCGTTTATCGCCTGACAGTCATTATCGATGAT
 G  G  G  S  G  S  I  I  N  E  T  A  D  D  I  V  Y  R  L  T  V  I  I  D  D
              BamHI         TDoT
CGCTACGAATCGCTGAAAAACCTGATTACCTTACGTGCAGATCGCTTGGAGATGATCATCAATGACAATGTGTCC
 R  Y  E  S  L  K  N  L  I  T  L  R  A  D  R  L  E  M  I  I  N  D  N  V  S

ACCATTCTCGCGAGCATTTAAGCGGCCGCACTCGAG
 T  I  L  A  S  I  *  A  A  A  L  E
                       NotI
```

*Ec*LDC-L-3HAMP

```
CATATGATGAACATCATCGCTATCATGGGCCCTCACGGTGTTTTCTACAAGGATGAGCCAATCAAGGAGCTGGAA
 H  M  M  N  I  I  A  I  M  G  P  H  G  V  F  Y  K  D  E  P  I  K  E  L  E
  NdeI                                                                    EcLDC
TCCGCACTAGTTGCACAGGGCTTTCAGATCATCTGGCCCCAGAACTCCGTTGACCTTCTCAAATTCATCGAGCAC
 S  A  L  V  A  Q  G  F  Q  I  I  W  P  Q  N  S  V  D  L  L  K  F  I  E  H

AATCCTCGCATTTGTGGTGTGATTTTTGACTGGGACGAGTACTCTCTTGATTTATGCTCCGACATCAACCAGCTC
 N  P  R  I  C  G  V  I  F  D  W  D  E  Y  S  L  D  L  C  S  D  I  N  Q  L

AACGAGTACCTGCCACTCTACGCATTCATCAACACTCACTCCACCATGGACGTTTCCGTGCAGGACATGCGTATG
 N  E  Y  L  P  L  Y  A  F  I  N  T  H  S  T  M  D  V  S  V  Q  D  M  R  M

GCACTCTGGTTCTTTGAATACGCTCTGGGCCAGGCTGAGGACATCGCGATCCGCATGCGTCAGTACACCGACGAG
 A  L  W  F  F  E  Y  A  L  G  Q  A  E  D  I  A  I  R  M  R  Q  Y  T  D  E

TACCTGGACAACATCACCCCTCCATTCACCAAGGCTCTCTTCACCTACGTAAAGGAACGCAAGTACACTTTCTGC
 Y  L  D  N  I  T  P  P  F  T  K  A  L  F  T  Y  V  K  E  R  K  Y  T  F  C

ACCCCAGGCCACATGGGCGGCACCGCCTACCAGAAGTCCCCAGTCGGATGCCTCTTCTACGACTTCTTCGGCGGT
 T  P  G  H  M  G  G  T  A  Y  Q  K  S  P  V  G  C  L  F  Y  D  F  F  G  G

AACACTCTTAAGGCAGATGTCTCCATTTCCGTCACCGAGTTGGGCTCTCTGCTGGACCACACCGGCCCTCACCTG
 N  T  L  K  A  D  V  S  I  S  V  T  E  L  G  S  L  L  D  H  T  G  P  H  L

GAGGCAGAAGAGTACATCGCTCGTACCTTCGGTGCTGAACAGTCCTACATCGTCACCAACGGTACTTCCACCAGC
 E  A  E  E  Y  I  A  R  T  F  G  A  E  Q  S  Y  I  V  T  N  G  T  S  T  S

AACAAGATCGTTGGTATGTACGCAGCTCCTTCTGGCTCCACCCTGTTGATCGACCGCAACTGTCACAAGTCCCTC
 N  K  I  V  G  M  Y  A  A  P  S  G  S  T  L  L  I  D  R  N  C  H  K  S  L

GCGCATCTTCTTATGATGAACGATGTGGTCCCTGTATGGCTGAAGCCAACCCGTAACGCTCTGGGCATCCTTGGC
```

A H L L M M N D V V P V W L K P T R N A L G I L G

GGTATCCCCCGTCGCGAGTTCACCCGTGATTCCATCGAGGAAAAGGTTGCAGCCACTACCCAGGCACAGTGGCCT
G I P R R E F T R D S I E E K V A A T T Q A Q W P

GTCCACGCTGTCATTACCAACTCGACCTACGACGGCCTGCTCTACAACACCGATTGGATCAAGCAGACCCTAGAT
V H A V I T N S T Y D G L L Y N T D W I K Q T L D

GTTCCTTCCATTCACTTCGACAGCGCATGGGTTCCTTACACTCACTTCCACCCAATCTACCAGGGTAAGTCCGGA
V P S I H F D S A W V P Y T H F H P I Y Q G K S G

ATGTCCGGCGAGCGTGTCGCTGGCAAGGTTATCTTCGAAACCCAATCAACCCACAAGATGCTGGCTGCTCTCTCC
M S G E R V A G K V I F E T Q S T H K M L A A L S

CAGGCTTCTCTGATCCACATCAAGGGCGAGTACGACGAGGAAGCTTTCAACGAGGCTTTCATGATGCACACCACC
Q A S L I H I K G E Y D E E A F N E A F M M H T T

ACCTCCCCATCCTACCCTATCGTCGCGTCCGTCGAGACTGCTGCCGCAATGCTTCGCGGTAACCCAGGTAAGCGC
T S P S Y P I V A S V E T A A A M L R G N P G K R

CTCATCAACCGTTCCGTTGAGCGCGCTCTTCACTTCCGTAAGGAAGTGCAGCGCCTGCGTGAGGAATCTGACGGT
L I N R S V E R A L H F R K E V Q R L R E E S D G

TGGTTCTTCGACATTTGGCAGCCACCTCAGGTTGATGAGGCCGAGTGCTGGCCAGTTGCTCCAGGTGAACAGTGG
W F F D I W Q P P Q V D E A E C W P V A P G E Q W

CACGGATTCAACGATGCAGATGCTGACCACATGTTTTTGGACCCGGTCAAGGTCACCATTCTTACTCCTGGTATG
H G F N D A D A D H M F L D P V K V T I L T P G M

GATGAGCAGGGCAACATGTCTGAGGAGGGTATCCCAGCTGCTCTGGTTGCAAAGTTCCTCGACGAACGTGGCATC
D E Q G N M S E E G I P A A L V A K F L D E R G I

GTTGTTGAGAAGACCGGACCATACAACCTGCTGTTCCTGTTCAGCATCGGCATCGACAAAACCAAGGCAATGGGT
V V E K T G P Y N L L F L F S I G I D K T K A M G

CTGCTGCGCGGCCTTACCGAGTTCAAGCGCTCCTACGACCTGAACCTTCGCATCAAGAATATGCTGCCGGACCTG
L L R G L T E F K R S Y D L N L R I K N M L P D L

TACGCTGAAGATCCTGATTTCTACCGCAACATGCGCATCCAGGACCTCGCACAGGGCATCCACAAGCTCATTCGC
Y A E D P D F Y R N M R I Q D L A Q G I H K L I R

AAGCACGACCTGCCAGGCCTTATGCTCCGTGCATTCGATACCCTCCCAGAGATGATCATGACCCCTCACCAGGCT
K H D L P G L M L R A F D T L P E M I M T P H Q A

TGGCAGCGCCAGATCAAGGGCGAGGTGGAAACCATCGCACTGGAGCAGCTGGTTGGTCGTGTCTCCGCCAACATG
W Q R Q I K G E V E T I A L E Q L V G R V S A N M

ATCCTGCCATATCCACCTGGCGTTCCGCTGCTGATGCCAGGCGAGATGCTCACCAAGGAGTCCCGCACCGTATTG
I L P Y P P G V P L L M P G E M L T K E S R T V L

GACTTCCTTCTCATGTTGTGCTCTGTTGGCCAGCACTACCCAGGCTTCGAGACCGACATCCACGGCGCTAAGCAA
D F L L M L C S V G Q H Y P G F E T D I H G A K Q

GATGAAGACGGCGTTTACCGCGTTCGCGTCCTTAAGATGGCAGGCGCTAGCGGCGGTGGGTCTGGAGGCGGCTCA
D E D G V Y R V R V L K M A G **A S** G G G S G G G S
***Nhe*I** **3xGGGS-Linker**

GGTGGTGGGTCGGGATCCATGGGCCTGTTTAACGCCCATGCAGTTGCGCAGCAACGCGCGGATCGCATTGCGACT
G G G S **G S** M G L F N A H A V A Q Q R A D R I A T
***Bam*HI** 3HAMP

CTCCTGCAGTCCTTTGCGGATGGTCAGTTGGACACCGCCGTGGGTGAAGCGCCAGCACCTGGTTACGAACGCCTG
L L Q S F A D G Q L D T A V G E A P A P G Y E R L

TATGACTCGCTTCGCGCCCTTCAGCGCCAACTGCGCGAACAACGTGCGGAGTTACAACAGGTTGAGAGCCTGGAA

```
 Y  D  S  L  R  A  L  Q  R  Q  L  R  E  Q  R  A  E  L  Q  Q  V  E  S  L  E

GCAGGCTTGGCTGAAATGAGTCGGCAGCATGAAGCAGGGTGGATTGACCAGACGATTCCGGCTGAACGGTTAGAG
 A  G  L  A  E  M  S  R  Q  H  E  A  G  W  I  D  Q  T  I  P  A  E  R  L  E

GGCCGTGCAGCACGTATCGCCAAAGGCGTGAATGAGCTGGTTGCTGCGCACATTGCGGTGAAAATGAAAGTCGTG
 G  R  A  A  R  I  A  K  G  V  N  E  L  V  A  A  H  I  A  V  K  M  K  V  V

AGCGTAGTCACCGCGTATGGCCAAGGGAACTTCGAACCGCTCATGGATCGCCTGCCGGGTAAGAAAGCCCAGATC
 S  V  V  T  A  Y  G  Q  G  N  F  E  P  L  M  D  R  L  P  G  K  K  A  Q  I

ACGGAGGCCATTGATGGCGTACGTGAACGCCTGCGTGGAGCTGCTGAAGCGACCTCTGCGCAGCTGGCCACAGCC
 T  E  A  I  D  G  V  R  E  R  L  R  G  A  A  E  A  T  S  A  Q  L  A  T  A

GCCTACAATTAAGCGGCCGCACTCGAG
 A  Y  N  *  A  A  A  L  E
            NotI
```

References

1. Diener M, Kopka B, Pohl M, Jaeger KE, Krauss U. Fusion of a coiled-coil domain facilitates the high-level production of catalytically active enzyme inclusion bodies. Chemcatchem 2016; 8;142-152.
2. Bradford MM. A rapid and sensitive method for the quantitation of microgram quantities of protein utilizing the principle of protein-dye binding. Analytical Biochemistry 1976; 72;248-254.
3. Jäger VD, Lamm R, Kloss R, Kaganovitch E, Grünberger A, Pohl M, Büchs J, Jaeger K-E, Krauss U. A synthetic reaction cascade implemented by co-localization of two proteins within catalytically-active inclusion bodies. ACS Synthetic Biology 2018; [Epub ahead of print] doi: 10.1021/acssynbio.8b00274.
4. Kandiah E, Carriel D, Perard J, Malet H, Bacia M, Liu K, Chan SW, Houry WA, Ollagnier de Choudens S, Elsen S, Gutsche I. Structural insights into the *Escherichia coli* lysine decarboxylases and molecular determinants of interaction with the AAA+ ATPase RavA. Scientific Reports 2016; 6;24601.
5. Stetefeld J, Jenny M, Schulthess T, Landwehr R, Engel J, Kammerer RA. Crystal structure of a naturally occurring parallel right-handed coiled coil tetramer. Nature Structural Biology 2000; 7;772-776.
6. Airola MV, Watts KJ, Bilwes AM, Crane BR. Structure of concatenated HAMP domains provides a mechanism for signal transduction. Structure 2010; 18;436-448.
7. Conchillo-Sole O, de Groot NS, Aviles FX, Vendrell J, Daura X, Ventura S. AGGRESCAN: a server for the prediction and evaluation of "hot spots" of aggregation in polypeptides. BMC Bioinformatics 2007; 8;65.
8. Jacak R, Leaver-Fay A, Kuhlman B. Computational protein design with explicit consideration of surface hydrophobic patches. Proteins 2012; 80;825-838.
9. Kuhlman B, Baker D. Native protein sequences are close to optimal for their structures. Proceedings of the National Academy of Sciences 2000; 97;10383.
10. Rohl CA, Strauss CE, Misura KM, Baker D. Protein structure prediction using Rosetta. Methods Enzymol 2004; 383;66-93.
11. Diener M. Von gezielter Oligomerisierung zu katalytisch aktiven *inclusion bodies* – Eine alternative Strategie zur Stabilisierung und Immobilisierung von Biokatalysatoren. Heinrich-Heine University Düsseldorf, Institut für Molekulare Enzymtechnologie; 2014.
12. Kulig J, Simon RC, Rose CA, Husain SM, Hackh M, Ludeke S, Zeitler K, Kroutil W, Pohl M, Rother D. Stereoselective synthesis of bulky 1,2-diols with alcohol dehydrogenases. Catalysis Science & Technology 2012; 2;1580-1589.
13. Weckbecker A, Hummel W. Cloning, expression, and characterization of an (*R*)-specific alcohol dehydrogenase from *Lactobacillus kefir*. Biocatalysis and Biotransformation 2006; 24;380-389.
14. Janzen E, Muller M, Kolter-Jung D, Kneen MM, McLeish MJ, Pohl M. Characterization of benzaldehyde lyase from *Pseudomonas fluorescens*: A versatile enzyme for asymmetric C-C bond formation. Bioorganic Chemistry 2006; 34;345-361.
15. Kloss R, Karmainski T, Jäger VD, Hahn D, Grünberger A, Baumgart M, Krauss U, Jaeger K-E, Wiechert W, Pohl M. Catalytically active inclusion bodies of benzaldehyde lyase from *Pseudomonas fluorescens* for application in biocatalysis. ??? *to be submitted*.
16. Lingen B, Kolter-Jung D, Dünkelmann P, Feldmann R, Grötzinger J, Pohl M, Müller M. Alteration of the substrate specificity of benzoylformate decarboxylase from *Pseudomonas putida* by directed evolution. ChemBioChem 2003; 4;721-726.
17. Kloss R, Limberg MH, Mackfeld U, Hahn D, Grünberger A, Jäger VD, Krauss U, Oldiges M, Pohl M. Catalytically active inclusion bodies of L-lysine decarboxylase from *E. coli* for 1,5-diaminopentane production. In *Scientific reports*, vol. 8. pp. 58562018; 5856.
18. Studier FW, Moffatt BA. Use of bacteriophage T7 RNA polymerase to direct selective high-level expression of cloned genes. J Mol Biol 1986; 189;113-130.

19. Gasteiger E, Hoogland C, Gattiker A, Duvaud Se, Wilkins MR, Appel RD, Bairoch A. Protein identification and analysis tools on the ExPASy server. In *The Proteomics Protocols Handbook.* Edited by Walker JM. Totowa, NJ: Humana Press; 2005: 571-607.
20. Shaner NC, Steinbach PA, Tsien RY. A guide to choosing fluorescent proteins. Nature Methods 2005; 2;905-909.
21. Miyawaki A, Griesbeck O, Heim R, Tsien RY. Dynamic and quantitative Ca^{2+} measurements using improved cameleons. Proceedings of the National Academy of Sciences of the United States of America 1999; 96;2135-2140.
